# *xcms* in Peak Form: Now Anchoring a Complete Metabolomics Data Preprocessing and Analysis Software Ecosystem

**DOI:** 10.1021/acs.analchem.5c04338

**Published:** 2025-12-08

**Authors:** Philippine Louail, Carl Brunius, Mar Garcia-Aloy, William Kumler, Norman Storz, Jan Stanstrup, Hendrik Treutler, Pablo Vangeenderhuysen, Michael Witting, Steffen Neumann, Johannes Rainer

**Affiliations:** † Institute for Biomedicine, 198234 Eurac Research, 39100 Bolzano, Italy; ‡ Chair for Bioinformatics, Friedrich-Schiller-University Jena, 07743 Jena, Germany; § Department of Life Sciences, Food and Nutrition Science, Chalmers University of Technology, SE-412 96 Göteborg, Sweden; ∥ Metabolomics Unit, Research and Innovation Centre, Fondazione Edmund Mach, 38098 San Michele all’Adige (TN), Italy; ⊥ School of Oceanography, 7284 University of Washington, Seattle, Washington 98195, United States; # Leibniz Institute of Plant Biochemistry, MetaCom, Weinberg 3, 06120 Halle, Germany; ∇ Department of Nutrition, Exercise and Sports, University of Copenhagen, Rolighedsvej 26, 1958 Frederiksberg C, Denmark; ○ Laboratory of Integrative Metabolomics (LIMET), 26656 Ghent University, 9820 Merelbeke, Belgium; ◆ Metabolomics and Proteomics Core, Helmholtz Zentrum München, Ingolstädter Landstraße 1, 85764 Neuherberg, Germany; ¶ Chair of Analytical Food Chemistry, TUM School of Life Sciences, Technical University of Munich, Maximus-von-Imhof-Forum 2, 85354 Freising-Weihenstephan, Germany; ⋈ German Centre for Integrative Biodiversity Research (iDiv) Halle-Jena-Leipzig, Puschstraße 4, 04103 Leipzig, Germany

## Abstract

High-quality data preprocessing is essential for untargeted metabolomics experiments, where increasing data set scale and complexity demand adaptable, robust, and reproducible software solutions. Modern preprocessing tools must evolve to integrate seamlessly with downstream analysis platforms, ensuring efficient and streamlined workflows. Since its introduction in 2005, the *xcms* R package has become one of the most widely used tools for LC-MS data preprocessing. Developed through an open-source, community-driven approach, *xcms* maintains long-term stability while continuously expanding its capabilities and accessibility. We present recent advancements that position *xcms* as a central component of a modular and interoperable software ecosystem for metabolomics data analysis. Key improvements include enhanced scalability, enabling the processing of large-scale experiments with thousands of samples on standard computing hardware. These developments empower users to build comprehensive, customizable, and reproducible workflows tailored to diverse experimental designs and analytical needs. An expanding collection of tutorials, documentation, and teaching materials further supports both new and experienced users in leveraging broader R and Bioconductor ecosystems. These resources facilitate the integration of statistical modeling, visualization tools, and domain-specific packages, extending the reach and impact of *xcms* workflows. Together, these enhancements solidify *xcms* as a cornerstone of modern metabolomics research.

## Graphical abstract

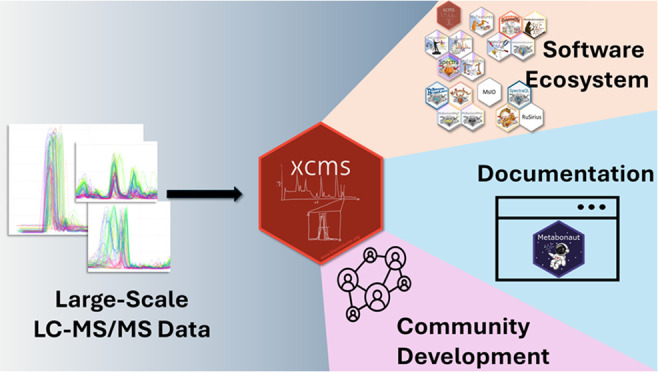

Preprocessing is the crucial first step in analyzing untargeted liquid chromatography–mass spectrometry (LC-MS) or gas chromatography–mass spectrometry (GC-MS) data. It involves quantifying ion signals in a sample, correcting potential retention time drifts within and across analytical runs, and grouping signals from the same ion species across all samples in an experiment. Preprocessing software must accommodate data from various column types, such as reversed-phase (RP) chromatography and hydrophilic interaction liquid chromatography (HILIC), which produce diverse metabolite separations and chromatographic peak structures. As high-resolution mass spectrometers like Quadrupole-Time-of-Flight (QTOF) and Orbitrap become increasingly common, and with growing demand for high-throughput metabolomics, data processing tools must keep pace with increasing data complexity and volume. Several key challenges have emerged: the need to handle large-scale data sets, ensure reproducibility and transparency, and provide flexibility for a wide range of experimental designs.

Originally introduced in 2005 ^1^ with chromatographic peak detection and retention time alignment algorithms, *xcms* has since been cited over 3800 times and was downloaded over 150,000 times by distinct IP addresses since the introduction of download statistics in Bioconductor in 2009. Over the two decades since its initial release, *xcms* has undergone significant transformations to address the aforementioned challenges while maintaining its core principles of flexibility, external collaboration, and open-source innovation. A key factor in its adaptability and longevity was its early integration into the Bioconductor project,^2,3^ making it one of the first metabolomics software packages in this ecosystem, now comprising over 2,200 packages (as of Bioconductor version 3.20). Beyond Bioconductor, *xcms* has also been incorporated into other platforms, such as *Galaxy Workflow4Metabolomics*,^4^ further extending its reach and usability.

Over the past two decades, innovations in LC-MS data acquisition have significantly reshaped the field of metabolomics, and data preprocessing has evolved in parallel. A wide array of software tools is now available to support various stages of analysis.^5^ In the context of preprocessing, *xcms* is accompanied by other well-established tools such as *mzmine* ^6^ and *MS-DIAL*,^7^ both of which have seen substantial development and feature expansion in recent years. Comparative descriptions of their respective strengths and limitations have been done before.^8–10^ *xcms* offers strengths in reproducibility, scalability, and expandability (with custom functions), although it requires some R/coding skills. Unlike vendor or Graphic User Interface (GUI)-based software, it therefore addresses a different target audience seeking flexibility and integrative workflows. Moreover, these tools operate outside the R and Bioconductor ecosystem, highlighting the growing importance of interoperability, an area that *xcms* developers have actively addressed. Recent efforts include the development of common export formats and supporting infrastructure designed to improve integration with external tools and programming languages beyond R.

It is important to clarify that the developments discussed in this article pertain specifically to the *xcms* R package and its evolution within the R/Bioconductor ecosystem. These updates do not apply to *XCMS Online* ^11^ or other web-based tools that use a different or older *xcms* code base and remain separate entities with distinct functionalities and development trajectories. This article summarizes the major technical and infrastructural advancements of the *xcms* R package over the past two decades. We present developments that have solidified *xcms* as one of the main tools for LC-MS data preprocessing. We discuss key advancements, including methodological improvements, expanded support for diverse mass spectrometry (MS) data formats, and integration with an evolving ecosystem of R packages for MS data analysis. Improvements to the underlying data structures and increased flexibility in the processing steps allow memory-saving data analysis even for very large data sets. Reproducible case studies are discussed throughout the article, offering concrete validation of the improvements described, providing practical guidance on workflow design, parameter selection, and downstream analysis.

As part of the Bioconductor ecosystem, *xcms* continues to foster a collaborative and adaptable environment for metabolomics research. Over the past 20 years, these innovations have transformed *xcms* into a comprehensive and scalable toolbox within the R environment, facilitating the development of versatile end-to-end workflows for LC-MS and LC-MS/MS data analysis.

## Data Analysis Capabilities

*xcms* has continuously evolved to meet the needs of scientists and data analysts, with several key implementations introduced to enhance its functionality. Central to these updates is the introduction of parameter classes, which form the foundation of a modular and flexible programming interface. Each major preprocessing step, such as peak detection, retention time alignment, and chromatographic peak grouping, is now handled by a dedicated function, with multiple algorithms available for each step. These algorithms are configured via dedicated parameter classes, allowing users to easily define and customize settings based on the specific requirements of their experiment. This structured approach simplifies workflow construction, enhances flexibility, and promotes reproducibility by embedding parameter settings directly into the processing history. As a result, *xcms* now provides an intuitive yet powerful interface that supports transparency and traceability throughout the data analysis process.

Key steps in *xcms* preprocessing have been significantly refined (Figure 1A), as detailed in the following section. For example, peak detection is now coupled with peak filtering and refinement, addressing artifacts that can arise in the initial steps by postprocessing detected chromatographic peaks and removing or merging them if necessary. Additionally, peak quality metrics, such as those presented in Kumler et al.,^15^ can be calculated either during or after peak detection, allowing users to filter the data and retain only high-confidence signals. These improved filtering approaches help minimize the inclusion of likely false-positive peaks, a common issue in earlier studies.^16–19^ The alignment process has also seen substantial improvements with the introduction of new algorithms and the option to base it on a subset of the data set or an external reference data set. The gap-filling step, designed to recover missing values for low-abundance features, has been improved by employing a more accurate estimation of the ions’ expected *m*/*z*–retention time region. Lastly, several methods for quality control of features have been developed, adhering to widely accepted standards in metabolomics.^20^ An end-to-end workflow using an example data set (Supporting File S1) illustrates these new preprocessing steps in detail.

**Figure 1:**
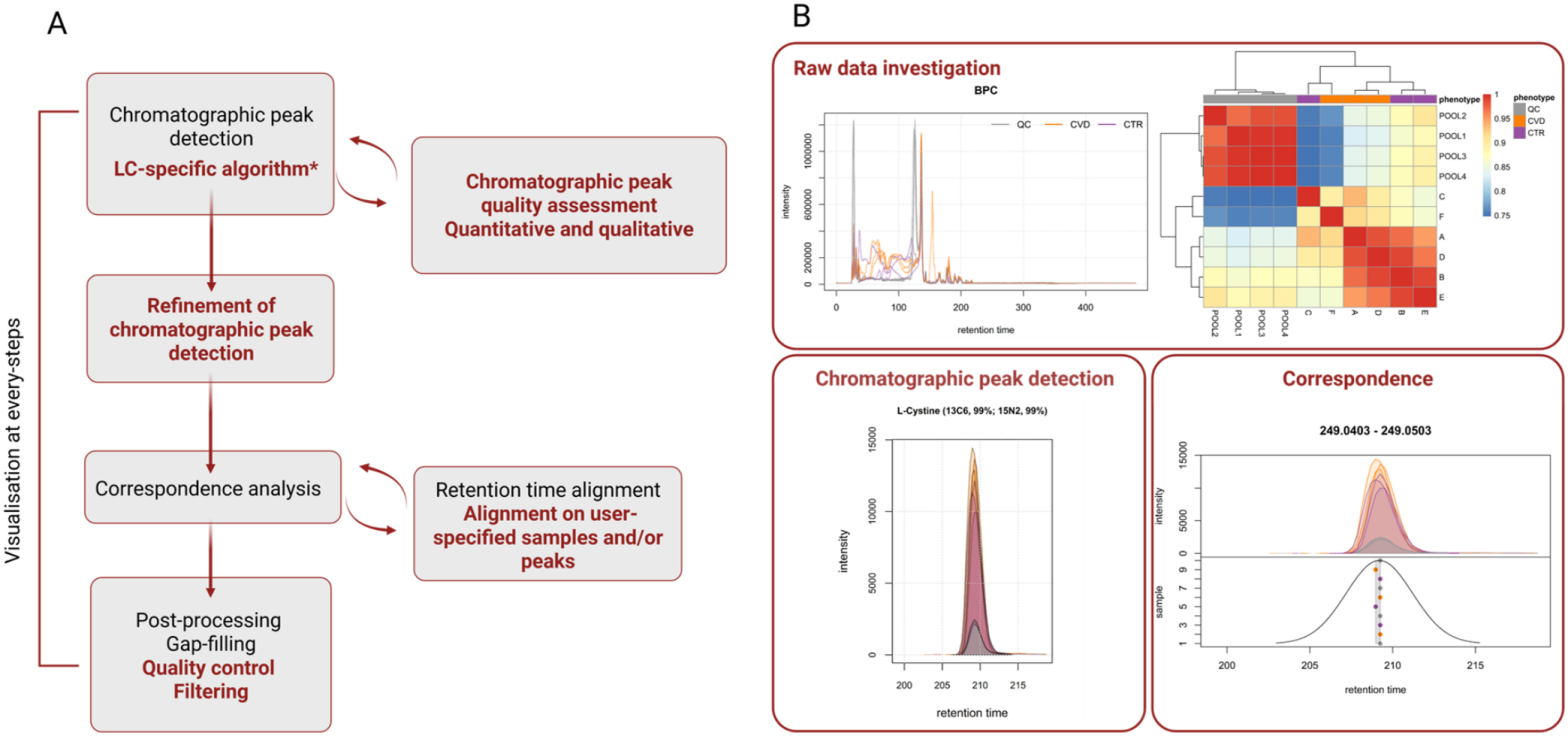
Graphical representation of key technical improvements in *xcms* over the past 20 years. (A) Preprocessing workflow in *xcms*: original steps from the 2006 paper are shown in black, while newly implemented improvements are highlighted in red, reflecting the continued development of *xcms* that preserves its core methodology while expanding its functionality and performance. * The new LC-specific algorithms are centWave^12^ and MassifQuant^13^. (B) Examples of possible visualizations of raw data (top) and data at different preprocessing stages (lower row), enabling evaluation at each step. The source code and data to produce these images are available in the tutorials on the *Metabonaut* Web site,^14^ which can be found: [Exploring and Analyzing LC-MS Data • Metabonaut]. Created in BioRender. Fuchsberger, C. (2025) https://BioRender.com/cuokujz.

The core data structures of *xcms* were completely rewritten to gain full support for LC-MS/MS data, as demonstrated in the Supporting File S2 and the *GNPS* molecular networking case study.^21^ These new data structures rely on the established MS data infrastructures of *MSnbase* ^22^ and packages from the *RforMassSpectrometry* initiative,^23–25^ in particular, the *Spectra* and *Chromatograms* R packages. Apart from a tighter integration with other R packages that will be discussed in the following section, this redesign significantly enhances *xcms*’ ability to handle, subset, and filter data while also ensuring compatibility with emerging data formats. To evaluate the performance improvements of the recent developments in *xcms*, we repeated the analysis of the large LC-MS/MS data set with 1039 samples from the aforementioned *GNPS* molecular networking study using different versions of *xcms* on the same computational setup (using 4 cores of a standard notebook computer with an Intel Core i7–1370P CPU and 64 GB total RAM). The total runtime of the analysis, which includes preprocessing, selection of features’ MS2 spectra, and export of the data for molecular networking with *GNPS*, took 860 min with the versions used in the original article^21^ (R 3.6.3, Bioconductor 3.10, *xcms* 3.8.3). In contrast, using the current versions (R 4.5.1, Bioconductor 3.22, *xcms* 4.7.3), the processing time was reduced to only 280 min, despite an additional peak refinement step being performed. In addition to this ∼3x reduction in total runtime, memory usage was also much lower. See Supporting Files S2 and S3 for the respective analysis reports, including runtime and memory usages of the individual analysis steps.

To further reduce memory demands, a new *xcms* result object with a very low memory footprint was implemented, allowing memory-saving data analysis even for very large data sets. In a second large-scale metabolomics analysis comprising ∼4000 samples from a public data set, preprocessing could be performed on a standard notebook computer (same hardware as described above), underscoring the scalability and memory efficiency of the redesigned system (Supporting File S4). Together, these results demonstrate that the enhanced *xcms* data structures and workflows enable high-throughput LC–MS/MS data processing on standard hardware, eliminating the need for specialized computing resources.

Beyond the improved data processing algorithms, significant emphasis has been placed on enhancing data visualization within *xcms.* The aforementioned redesigned architecture optimizes chromatographic data organization and accessibility, enabling users to generate and visualize extracted ion chromatograms (EICs) from large data sets throughout the entire analysis process. Combined with the robust plotting possibilities of R, this integration ensures an intuitive and powerful visualization experience. Users can test and fine-tune settings for each preprocessing step, leading to more informed and justifiable parameter choices. Tailored plotting functions have been developed for different stages of the analysis, whether working with the full, raw MS data or examining results at various preprocessing steps. Specifically, users can generate base peak chromatograms (BPCs) from raw data, EICs with highlighted chromatographic peaks during peak detection, and density plots for feature grouping, allowing for visual insights throughout the workflow (Figure 1B).

## Integration and Interoperability

The development of *xcms* and its surrounding ecosystem (Figure 2 and Supporting Table S1) is primarily driven by its integration within the Bioconductor^2,3^ community, which ensures adherence to high-standard software guidelines, including rigorous unit testing and documentation. This integration promotes open-source development, encourages community contributions, and supports direct user feedback through GitHub’s issue-tracking system as well as through the Bioconductor support site. Additionally, it enables *xcms* to leverage standardized data structures, such as *SummarizedExperiment*,^26^ the central Bioconductor data type for representing quantitative data from biological assays. This greatly simplifies interoperability with other Bioconductor packages, strengthening the ecosystem by improving data handling within *xcms* and enhancing downstream analyses, including data normalization, visualization, statistical data analysis, or annotation. The aforementioned end-to-end workflow demonstrates this integration of *xcms* within the broader R/Bioconductor mass spectrometry ecosystem (Supporting File S1).

**Figure 2:**
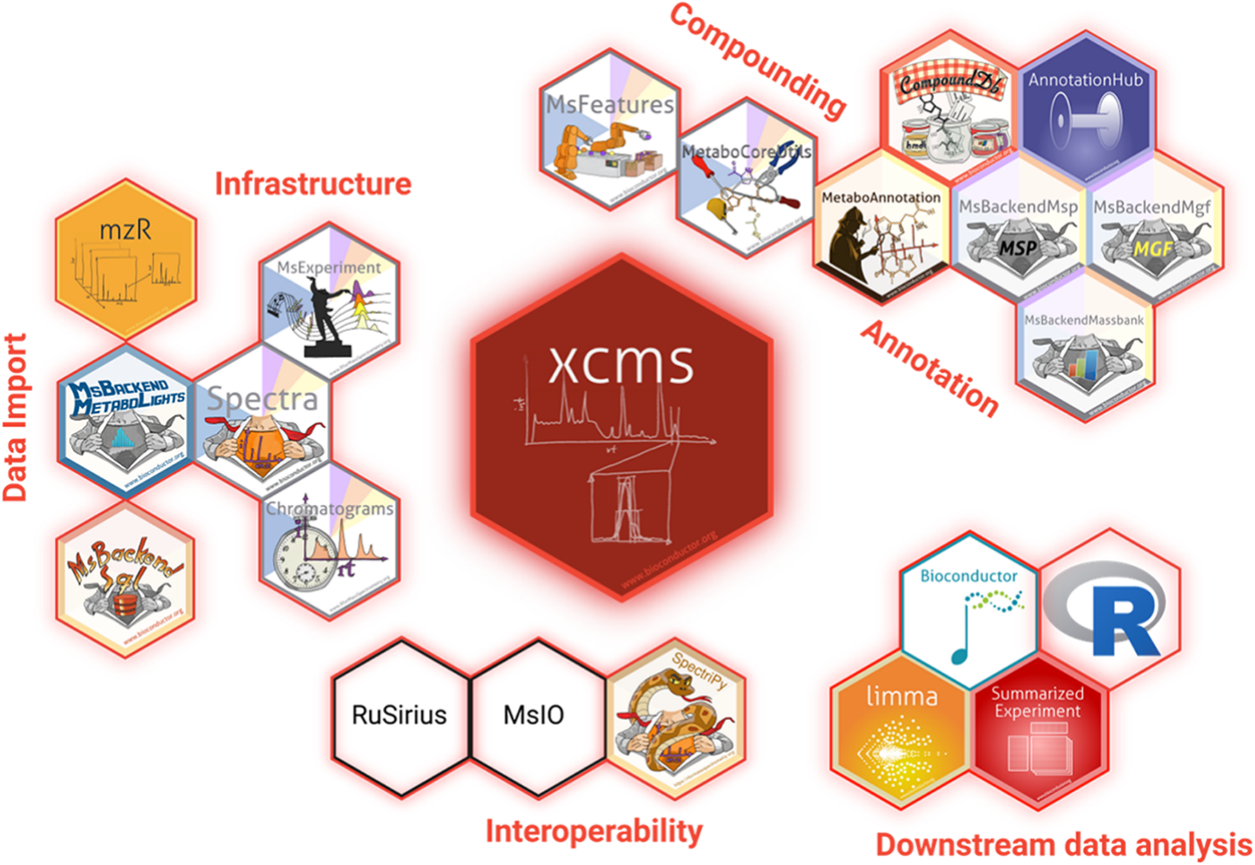
*xcms* is part of an ecosystem of packages, expanding the analysis possibilities. See Supporting Table S1 for a detailed listing and description of the packages in the figure and Stanstrup et al.^35^ for a complete overview of metabolomics R packages. Created in BioRender. Fuchsberger, C. (2025) https://BioRender.com/jxpxdq7.

Within the *RforMassSpectrometry* ^23,24^ initiative, various packages have been developed to serve as the basis for, or to complement, *xcms’* preprocessing possibilities. In this context, the previously mentioned *Spectra* and *Chromatograms* packages stand out as they efficiently manage the complexity of LC-MS data. The *MsFeatures* package adds yet another layer of versatility in postanalysis by grouping LC-MS *features* that represent the signal of ions and isotopes of the same compound. Meanwhile, the *MetaboCoreUtils* package provides researchers with a set of basic tools to extend and refine their analysis. The *MsExperiment* package provides a structured and user-oriented infrastructure that organizes experimental metadata and data files in a way that is directly compatible with *xcms*. Additionally, other packages in this initiative extend the utility of *xcms* beyond preprocessing, supporting postanalysis tasks. For instance, MS/MS spectra extracted from *xcms* objects can be used with the *MetaboAnnotation* ^27^ package for matching against reference fragment spectra. *xcms* integration within a rich ecosystem enables incorporating functionalities from diverse infrastructures into *xcms-*centered R workflows, reducing dependency on any single tool and thus enhancing both flexibility and scalability.

*xcms* has expanded its compatibility with external software workflows, extending its utility beyond R-based analyses. The objective is to foster a more inclusive and collaborative approach, enabling a broader user and developer base to collectively address the complex challenges inherent in LC-MS analysis. To achieve this level of interoperability, the *MsIO* package was introduced, supporting the import and export of *xcms* results in multiple file formats compatible with leading platforms. Examples include the support for the mzTab-M^28^ format, which will serve as the foundation for future integration with *mzmine* ^6^ and *MS-DIAL* ^7^ as well as JSON-based and HDF5-based formats for enhanced embedding within the Galaxy platform.^29^ Additionally, *xcms* now supports direct data exchange with community repositories like *MetaboLights*,^30^ facilitating streamlined data sharing and reuse. Further expanding its reach, *xcms* supports integration with tools and libraries outside the R ecosystem, such as Python libraries *matchms* ^31^ and *spectrum_utils* ^32^ (via *SpectriPy* ^33^) and applications like *Sirius* ^34^ (through ongoing development of *RuSirius*). These integrations establish *xcms* as a cornerstone in a broader ecosystem of software tools, enabling collaboration and analysis across diverse platforms and technologies.

## User Experience and Enabling Reproducibility

While the scripting use of *xcms* might be challenging for new users, it enables one to create reproducible and customizable analysis workflows, which can be difficult to achieve in GUI-based software solutions. In particular, using *xcms* with R Markdown, or the recently introduced R interface of the Quarto system,^36^ allows one to document and describe the analysis and its results transparently. The improved integration of *xcms* with R and Bioconductor greatly simplifies the implementation of such workflows by avoiding tedious conversion and copying of the data. As the flexibility, scalability, and functionalities of *xcms* have grown, so has its complexity. Consequently, significant emphasis has been placed on enhancing documentation by writing reference manuals for all available functions and providing a range of tutorials. These resources (see Supporting Table S1 for links) provide examples to tune and derive settings for *xcms* preprocessing^37^ as well as small use case analyses to exemplify how the functionalities of *xcms* and other packages from the *RforMassSpectrometry* initiative can be combined for in-depth data inspection^38^ and small compound annotation.^27^

The most recent and significant addition to the documentation is the creation of an end-to-end data analysis workflow that takes the user from importing raw data from *MetaboLights*,^30^ over LC-MS data preprocessing, quality assessment, data normalization, statistical data analysis, and finally the annotation of the identified significant features. This workflow is shared, as part of the *Metabonaut* resource, publicly on GitHub, where it is accompanied by targeted tutorials covering specific parts of analysis such as raw data investigation, integration with external tools such as Python libraries, *notame*,^39^ all within an R session. This workflow, along with accompanying vignettes, represents the culmination of nearly two decades of *xcms* evolution, transforming it from a standalone preprocessing tool into a component of a much more extensive, easily scalable, and customizable toolkit for LC-MS data analysis. It also signals the future direction of *xcms* and the related software ecosystem: greater integration, enhanced interoperability, and a stronger emphasis on community collaboration and education in the field.

## Conclusion

*xcms* has evolved far beyond its initial role as a preprocessing tool, becoming a core component of a flexible, scalable, and interoperable LC-MS/MS data analysis ecosystem. Its deep integration with Bioconductor has enabled advanced users to construct workflows that link preprocessing, quality control, statistical modeling, and annotation. Through GitHub discussions, community workshops, and initiatives such as *Metabonaut*, *xcms* has fostered an inclusive and collaborative user base, supporting novice users and experts. A key factor in *xcms’* long-term success is its adaptability to emerging analytical challenges. The implementation of dynamic parameter classes has introduced a more transparent and customizable workflow structure. In parallel, algorithmic refinements and enhanced visualization functions have increased both the accuracy and the interpretability of results.

Nevertheless, some limitations remain. For example, while support for ion mobility data exists at the backend level, current preprocessing algorithms do not yet exploit the ion mobility dimension. Also, although powerful, *xcms* can present a steep learning curve for new users due to its programmatic interface. However, ongoing efforts are underway to address these concerns. Graphical user interfaces such as *Metaboseek*,^40^ *patRoon*,^41^ and *Galaxy* workflows provide GUI and coding-free experience for interactive data exploration and preprocessing, making the ecosystem more accessible.

Interoperability remains a major strength of *xcms*. It aims to connect with external platforms like *GNPS*,^21,42^ *Sirius*, and *MetaboLights*,^30^ making it a valuable part of the global metabolomics infrastructure. Extensive tutorials, teaching resources, and community-contributed workflows have significantly improved the user experience and supported adoption across disciplines.

Through rigorous software development, open-source collaboration, and a strong community foundation, *xcms* has positioned itself as a modern, adaptable tool that continues to grow with the evolving needs of metabolomics research. As data sets increase in scale and complexity, *xcms* remains well equipped to support the next generation of scientific discoveries.

## Supplementary Material











## Data Availability

The *xcms* software is publicly available through Bioconductor at 10.18129/B9.bioc.xcms. The latest development version can be found on GitHub at https://github.com/sneumann/xcms. The software is licensed under the GPL (≥2) license. The full license information can be accessed here. *Metabonaut* v1.2.0 is publicly available at https://rformassspectrometry.github.io/Metabonaut/, where the complete end-to-end workflow, alignment based on external references, raw data investigation, a *SpectriPy* tutorial, and a demonstration of large-scale data set preprocessing can be found. The images used in Figure 1 are also part of the end-to-end workflow. The files for the reanalysis of the large LC-MS/MS data set from Nothias et al.^21^ are available on GitHub: https://github.com/jorainer/xcms-gnps-large-scale and Zenodo 10.5281/zenodo.17293665.

## Notes

File with a complete *xcms*-based end-to-end metabolomics data analysis workflow (Supporting File S2) (PDF)
Analysis report file (PDF format) of the preprocessing of a large LC-MS/MS data set for molecular networking with GNPS. Analysis performed with R version 3.6.3, Bioconductor 3.10, and *xcms* 3.8.3 (Supporting File S2) (PDF)
Analysis report file (PDF format) of the preprocessing of a large LC-MS/MS data set for molecular networking with GNPS. Analysis performed with R version 4.5.1, Bioconductor 3.22, and *xcms* 4.7.3 (Supporting File S3) (PDF)
Analysis report file (PDF format) for memory-saving *xcms*-based preprocessing of a large LC-MS data set (Supporting File S4) (PDF)
Table (PDF format) listing packages present in the *xcms* toolkit and their respective information (description, status of development, repository link), therefore expanding Figure 2 (Supporting Table S1) (PDF)
