# Supplementary material for: xcms in Peak Form: Now Anchoring a Complete Metabolomics Data Preprocessing and Analysis Software Ecosystem
Source: Anal Chem. 2025 Dec 8;97(50):27639–45. doi: 10.1021/acs.analchem.5c04338 (PMC12750407; doi:10.1021/acs.analchem.5c04338)
Supplement: Supplementary file 1 [file ac5c04338_si_001.pdf]

Complete end-to-end LC-MS/MS Metabolomic Data analysis

Supporting Information for: *xcms in peak form: Now anchoring a complete metabolomics data preprocessing and analysis software ecosystem*

Philippine Louail[1,2], Carl Brunius[3], Mar Garcia-Aloy[4], William Kumler[5], Norman Storz[6], Jan Stanstrup[7], Hendrik Treutler[6], Pablo Vangeenderhuysen[8], Michael Witting[9,10], Steffen Neumann[6,11,*], Johannes Rainer[1]

[1]Institute for Biomedicine, Eurac Research, 39100, Bolzano, Italy.
[2]Chair for Bioinformatics, Friedrich-Schiller-University Jena, 07743, Jena, Germany.
[3]Department of Life Sciences, Food and Nutrition Science, Chalmers University of Technology, SE-412 96 Göte-borg, Sweden.
[4]Metabolomics Unit, Research and Innovation Centre, Fondazione Edmund Mach, 38098, San Michele all'Adige (TN), Italy.
[5]School of Oceanography, University of Washington, Seattle, WA, 98195, USA.
[6]Leibniz Institute of Plant Biochemistry, MetaCom, Weinberg 3, 06120 Halle, Germany.
[7]Department of Nutrition, Exercise and Sports, University of Copenhagen, Rolighedsvej 26, 1958 Frederiksberg C, Denmark.
[8]Laboratory of Integrative Metabolomics (LIMET), Ghent University, 9820 Merelbeke, Belgium.
[9]Metabolomics and Proteomics Core, Helmholtz Zentrum München, Ingolstädter Landstraße 1, 85764 Neuherberg, Germany.
[10]Chair of Analytical Food Chemistry, TUM School of Life Sciences, Technical University of Munich, Maximus-von-Imhof-Forum 2, 85354 Freising-Weihenstephan, Germany.
[11]German Centre for Integrative Biodiversity Research (iDiv) Halle-Jena-Leipzig, Puschstraße 4, 04103 Leipzig, Germany.
[*]corresponding author: sneumann@ipb-halle.de

Abstract

This document describes a full *end-to-end* LC-MS/MS data analysis workflow including data preprocessing, quality assessment, normalization, statistical data analysis and annotation. It exemplifies the seamless integration of the *xcms* R package with other packages from the RforMassSpectrometry initiative and the Bioconductor project to create powerful, data set-specific, analysis workflows.

Introduction

The present workflow describes all steps for the analysis of an LC-MS/MS experiment, which includes the preprocessing of the raw data to generate the *abundance* matrix for the *features* in the various samples, followed by data normalization, differential abundance analysis and finally the annotation of features to metabolites. Note that also alternative analysis options and R packages could be used for different steps and some examples are mentioned throughout the workflow.

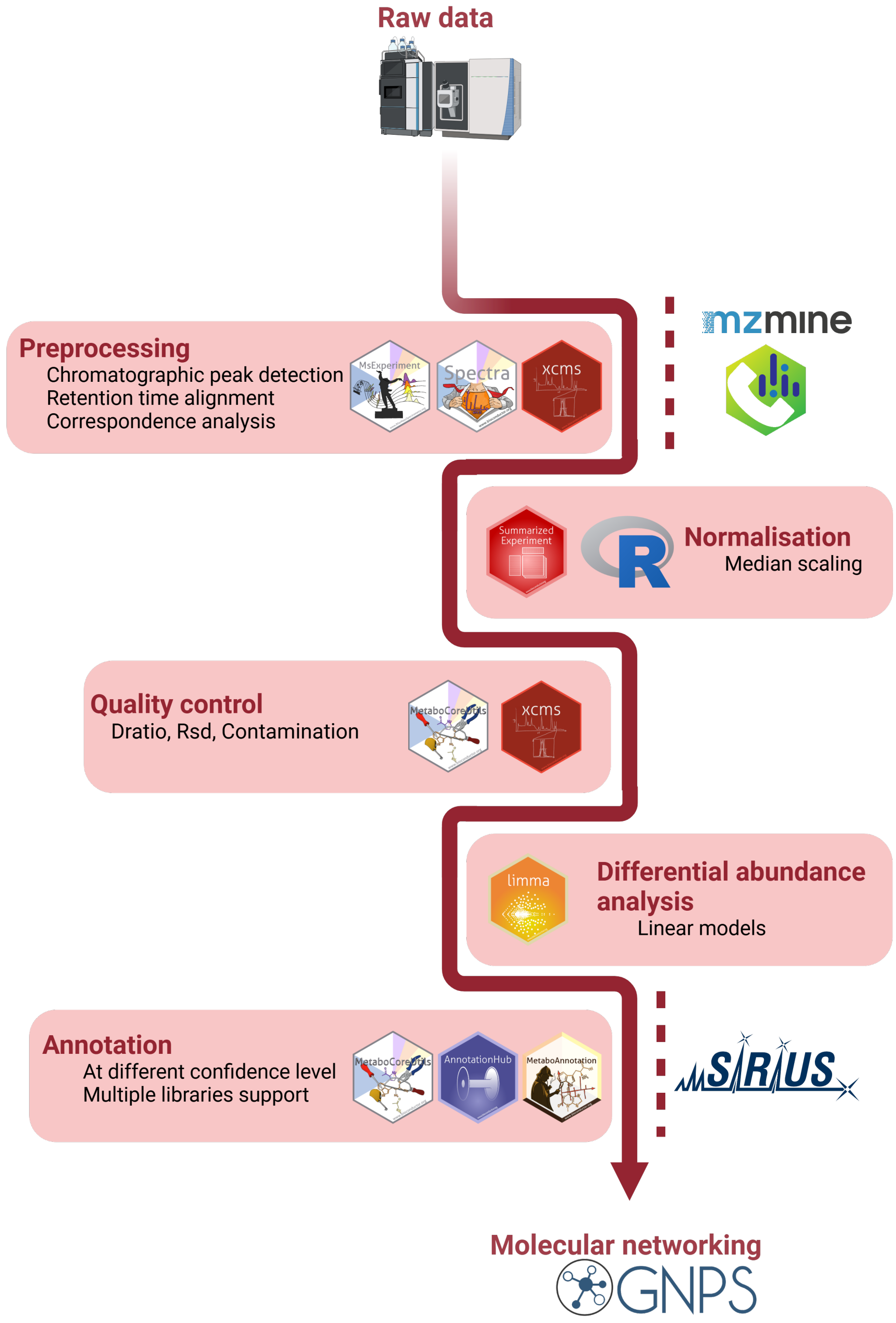

Figure 1: Steps of the end-to-end workflow and possible alternatives

Data description

See the data description vignette for detailed explanation of the dataset we go through in this workflow and general tips on what should be done when you first get your data.

Packages needed

All of the packages below can be installed through Bioconductor, using the `BiocManager::install()` function (*BiocManager*, if not available on the system, can be installed with `install.package("BiocManager")`) The exception is the *MsIO* package which is not yet available through Bioconductor and needs to be installed from GitHub using `BiocManager::install("RforMassSpectrometry/MsIO")`.

```
## Data Import and handling
library(knitr)
library(readxl)
library(MsExperiment)
library(MsIO)
library(alabaster.se)
library(MsBackendMetaboLights)
library(SummarizedExperiment)

## Preprocessing of LC-MS data
library(xcms)
library(Spectra)
library(MetaboCoreUtils)

## Statistical analysis
library(limma) # Differential abundance
library(matrixStats) # Summaries over matrices

## Visualisation
library(pander)
library(RColorBrewer)
library(pheatmap)
library(vioplot)
library(ggfortify)   # Plot PCA
library(gridExtra)   # To arrange multiple ggplots into single plots

## Annotation
library(AnnotationHub) # Annotation resources
```

```r
library(CompoundDb)    # Access small compound annotation data.
library(MetaboAnnotation) # Functionality for metabolite annotation.
```

Data import

Note that different equipment will generate various file extensions, so a conversion step might be needed beforehand, though it does not apply to this dataset. The Spectra package supports a variety of ways to store and retrieve MS data, including mzML, mzXML, CDF files, simple flat files, or database systems. If necessary, several tools, such as ProteoWizard's MSConvert, can be used to convert files to the .mzML format (Chambers et al. 2012).

Below we will show how to extract our dataset from the MetaboLigths database and load it as an `MsExperiment` object. For more information on how to load your data from the MetaboLights database, refer to the vignette. For other type of data loading, check out this xcms vignette A more specific vignette will be created for data import soon.

```r
param <- MetaboLightsParam(mtblsId = "MTBLS8735",
                           assayName = paste0("a_MTBLS8735_LC-MS_positive_",
                                              "hilic_metabolite_profiling.txt"),
                           filePattern = ".mzML")

lcms1 <- readMsObject(MsExperiment(),
                      param,
                      keepOntology = FALSE,
                      keepProtocol = FALSE,
                      simplify = TRUE)
```

Detailed information on the dataset is provided on MetaboLights MTBLS8735. In brief, the samples represent human blood samples, as quality control samples (QC) an external QC was used (pool of human serum samples). All samples were analyzed using ultra-high-performance liquid chromatography (UHPLC) (Agilent 1290; Agilent Technologies, Santa Clara, CA, USA) coupled to a Q-TOF mass spectrometer (TripleTOF 5600+; AB Sciex, Foster City, CA, USA). The chromatographic separation was based on hydrophilic interaction liquid chromatography (HILIC) and performed using an Acquity BEH amide, 100 x 2.1 mm column (Waters Corporation, Milford, MA, USA). The mass spectrometer was operated in full scan mode in the mass range from 50 to 1000 m/z and with an accumulation time of 250 ms. For LC-MS/MS data, the same settings were applied. Accumulation time for MS1 was set to 250 ms and for MS2 to 50 ms. Ions were selected for fragmentation based on an inclusion list and selecting the top 17 ions based on a data dependent acquisition (DDA) strategy.

We next configure the parallel processing setup. Most functions from the *xcms* package allow per-sample parallel processing, which can improve the performance of the analysis, especially for large data sets. *xcms* and all packages from the *RforMassSpectrometry* package ecosystem use the parallel processing setup configured through the *BiocParallel* Bioconductor package. With the code below we use a *fork-based* parallel processing on unix system, and a *socket-based* parallel processing on the Windows operating system.

```
#' Set up parallel processing using 2 cores
if (.Platform$OS.type == "unix") {
    register(MulticoreParam(2))
} else {
    register(SnowParam(2))
}
```

Data organisation

The experimental data is now represented by a `MsExperiment` object from the *MsExperiment* package. The `MsExperiment` object is a container for metadata and spectral data that provides and manages also the linkage between samples and spectra.

```
lcms1
```

```
Object of class MsExperiment
 Spectra: MS1 (17210)
 Experiment data: 10 sample(s)
 Sample data links:
  - spectra: 10 sample(s) to 17210 element(s).
```

Below we provide a brief overview of the data structure and content. The `sampleData()` function extracts sample information from the object. We next extract this data and use the *pander* package to render and show this information in Table 1 below. Throughout the document we use the R pipe operator (`|>`) to avoid nested function calls and hence improve code readability.

The `sampleData()` output from *MetaboLights* is not always ideal for direct and easy access to the data. We therefore rename it and transform it in a more user-friendly way. The user can add, transform and remove any column they want using base R functionalities.

```r
sampleData(lcms1)[, c("Derived_Spectral_Data_File",
                      "Characteristics[Sample type]",
                      "Factor Value[Phenotype]",
                      "Sample Name",
                      "Factor Value[Age]")] |>
    as.data.frame() |>
    pandoc.table(style = "rmarkdown")
```

Derived_Spectral_Data_File	Characteristics.Sample.type.
FILES/MS_QC_POOL_1_POS.mzML	pool
FILES/MS_A_POS.mzML	experimental sample
FILES/MS_B_POS.mzML	experimental sample
FILES/MS_QC_POOL_2_POS.mzML	pool
FILES/MS_C_POS.mzML	experimental sample
FILES/MS_D_POS.mzML	experimental sample
FILES/MS_QC_POOL_3_POS.mzML	pool
FILES/MS_E_POS.mzML	experimental sample
FILES/MS_F_POS.mzML	experimental sample
FILES/MS_QC_POOL_4_POS.mzML	pool

Table 1: Table continues below

Factor.Value.Phenotype.	Sample.Name	Factor.Value.Age.
	POOL	NA
CVD	A	53
CTR	B	30
	POOL	NA
CTR	C	66
CVD	D	36
	POOL	NA
CTR	E	66
CVD	F	44
	POOL	NA

Samples from the data set.

```r
# Let's rename the column for easier access
colnames(sampleData(lcms1)) <- c("sample_name", "derived_spectra_data_file",
                                 "metabolite_asssignment_file",
                                 "source_name",
                                 "organism",
                                 "blood_sample_type",
                                 "sample_type", "age", "unit", "phenotype")

# Add "QC" to the phenotype of the QC samples
qc_idx <- which(sampleData(lcms1)$sample_name == "POOL")
sampleData(lcms1)$phenotype[qc_idx] <- "QC"
sampleData(lcms1)$sample_name[qc_idx] <- c("POOL1", "POOL2",
                                           "POOL3", "POOL4")

#  Add injection index column
sampleData(lcms1)$injection_index <- seq_len(nrow(sampleData(lcms1)))

# let's look at the updated sample data
sampleData(lcms1)[, c("derived_spectra_data_file",
                      "phenotype", "sample_name", "age",
                      "injection_index")] |>
    as.data.frame() |>
    pandoc.table(style = "rmarkdown")
```

derived_spectra_data_file	phenotype	sample_name	age
FILES/MS_QC_POOL_1_POS.mzML	QC	POOL1	NA
FILES/MS_A_POS.mzML	CVD	A	53
FILES/MS_B_POS.mzML	CTR	B	30
FILES/MS_QC_POOL_2_POS.mzML	QC	POOL2	NA
FILES/MS_C_POS.mzML	CTR	C	66
FILES/MS_D_POS.mzML	CVD	D	36
FILES/MS_QC_POOL_3_POS.mzML	QC	POOL3	NA
FILES/MS_E_POS.mzML	CTR	E	66
FILES/MS_F_POS.mzML	CVD	F	44
FILES/MS_QC_POOL_4_POS.mzML	QC	POOL4	NA

Table 3: Table continues below

injection_index
1
2
3
4
5
6
7
8
9
10

Simplified sample data.

There are 10 samples in this data set. Below are abbreviations essential for proper interpretation of this metadata information:

- *phenotype*: The sample groups of the experiment:
  - `"QC"`: Quality control sample (pool of serum samples from an external, large cohort).
  - `"CVD"`: Sample from an individual with a cardiovascular disease.
  - `"CTR"`: Sample from a presumably healthy control.
- *sample_name*: An arbitrary name/identifier of the sample.
- *age*: The (rounded) age of the individuals.
- *injection_index*: An index representing the order (position) in which an individual sample was measured (injected) within the LC-MS measurement run of the experiment.

We will define colors for each of the sample groups based on their sample group using the *RColorBrewer* package:

```
#' Define colors for the different phenotypes
col_phenotype <- brewer.pal(9, name = "Set1")[c(9, 5, 4)]
names(col_phenotype) <- c("QC", # grey
                          "CVD", # orange
                          "CTR") # purple
col_sample <- col_phenotype[sampleData(lcms1)$phenotype]
```

The MS data of this experiment is stored as a `Spectra` object (from the *Spectra* Bioconductor package) within the `MsExperiment` object and can be accessed using `spectra()` function. Each element in this object is a spectrum - they are organised linearly and are all combined in the same `Spectra` object one after the other (ordered by retention time and samples).

Below we access the dataset's `Spectra` object which will summarize its available information and provide, among other things, the total number of spectra of the data set.

```
#' Access Spectra Object
spectra(lcms1)
```

```
MSn data (Spectra) with 17210 spectra in a MsBackendMetaboLights backend:
        msLevel     rtime scanIndex
      <integer> <numeric> <integer>
1             1     0.274         1
2             1     0.553         2
...         ...       ...       ...
17209         1   479.889      1720
17210         1   480.168      1721
 ... 37 more variables/columns.

file(s):
MS_QC_POOL_1_POS.mzML
MS_A_POS.mzML
MS_B_POS.mzML
 ... 7 more files
```

Data visualization and general quality assessment

Effective visualization is paramount for inspecting and assessing the quality of MS data. For a general overview of our LC-MS data, we can:

- Combine all mass peaks from all (MS1) spectra of a sample into a single spectrum in which each mass peak then represents the maximum signal of all mass peaks with a similar *m/z*. This spectrum might then be called Base Peak Spectrum (BPS), providing information on the most abundant ions of a sample.
- Aggregate mass peak intensities for each spectrum, resulting in the Base Peak Chromatogram (BPC). The BPC shows the highest measured intensity for each distinct retention time (hence spectrum) and is thus orthogonal to the BPS.
- Sum the mass peak intensities for each spectrum to create a Total Ion Chromatogram (TIC).
- Compare the BPS of all samples in an experiment to evaluate similarity of their ion content.
- Compare the BPC of all samples in an experiment to identify samples with similar or dissimilar chromatographic signal.

Note on the Base Peak Spectrum: evaluating and comparing the BPS within an experiment can help identifying potential problematic or contaminated samples. Also, it provides a general information on the *ion* content of a sample, i.e., whether ions with low or high m/z are present.

In addition to such general data evaluation and visualization, it is also crucial to investigate specific signal of e.g. internal standards or compounds/ions known to be present in the samples. By providing a reliable reference, internal standards help achieve consistent and accurate analytical results.

As LC signal is the most variable because of the unstable nature of the LC, we will only evaluate this below. More in depth inspection and discussion of spectra and chromatrograhic data can be found in the data exploration vignette.

Chromatographic Data Visualization: BPC and TIC

The `chromatogram()` function facilitates the extraction of intensities along the retention time. However, access to chromatographic information is currently not as efficient and seamless as it is for spectral information. Work is underway to develop/improve the infrastructure for chromatographic data through a new `Chromatograms` object aimed to be as flexible and user-friendly as the `Spectra` object.

For visualizing LC-MS data, a BPC or TIC serves as a valuable tool to assess the performance of liquid chromatography across various samples in an experiment. In our case, we extract the BPC from our data to create such a plot. The BPC captures the maximum peak signal from each spectrum in a data file and plots this information against the retention time for that spectrum on the y-axis. The BPC can be extracted using the `chromatogram` function.

By setting the parameter `aggregationFun = "max"`, we instruct the function to report the maximum signal per spectrum. Conversely, when setting `aggregationFun = "sum"`, it sums up all intensities of a spectrum, thereby creating a TIC.

```
#' Extract and plot BPC for full data
bpc <- chromatogram(lcms1, aggregationFun = "max")

plot(bpc, col = paste0(col_sample, 80), main = "BPC", lwd = 1.5)
grid()
legend("topright", col = col_phenotype,
       legend = names(col_phenotype), lty = 1, lwd = 2, horiz = TRUE,
       bty = "n")
```

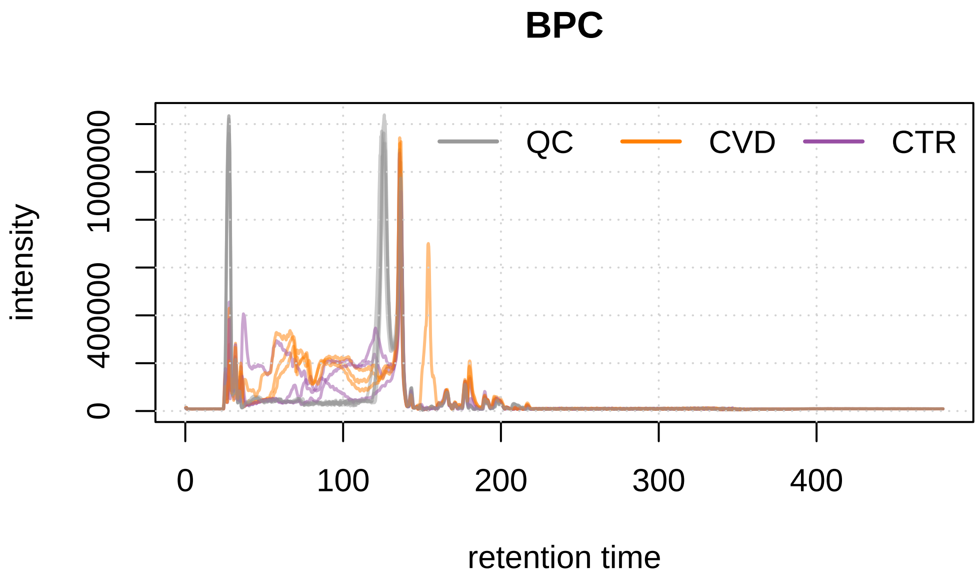

Figure 2: BPC of all samples colored by phenotype.

After about 240 seconds no signal seems to be measured. Thus, we filter the data removing that part as well as the first 10 seconds measured in the LC run.

```
#' Filter the data based on retention time
lcms1 <- filterRt(lcms1, c(10, 240))
```

```
Filter spectra
```

```
bpc <- chromatogram(lcms1, aggregationFun = "max")
```

```
#' Plot after filtering
plot(bpc, col = paste0(col_sample, 80),
     main = "BPC after filtering retention time", lwd = 1.5)
grid()
legend("topright", col = col_phenotype,
       legend = names(col_phenotype), lty = 1, lwd = 2,
       horiz = TRUE, bty = "n")
```

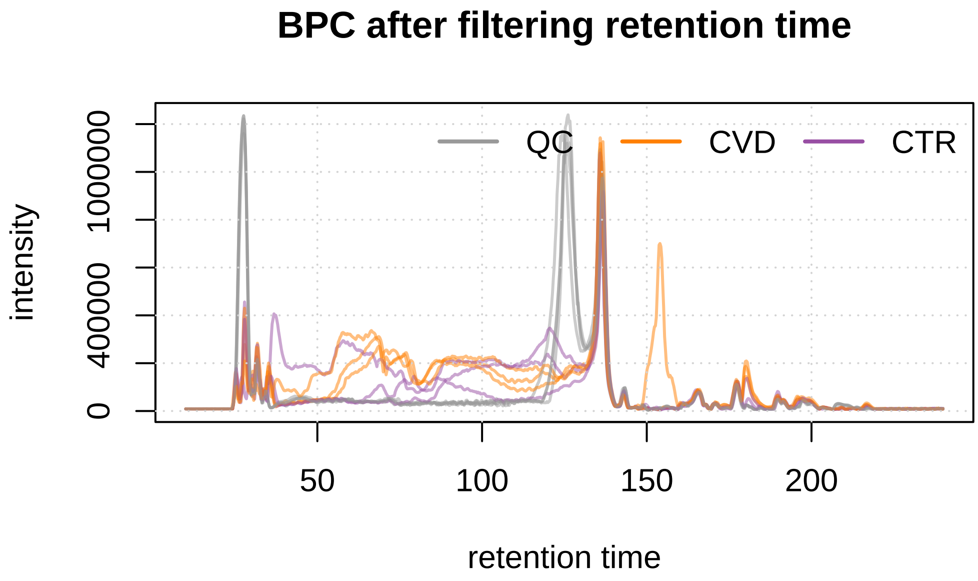

Figure 3: BPC after filtering retention time.

Initially, we examined the entire BPC and subsequently filtered it based on the desired retention times. This not only results in a smaller file size but also facilitates a more straightforward interpretation of the BPC.

The final plot illustrates the BPC for each sample colored by phenotype, providing insights on the signal measured along the retention times of each sample. It reveals the points at which compounds eluted from the LC column. In essence, a BPC condenses the three-dimensional LC-MS data (m/z by retention time by intensity) into two dimensions (retention time by intensity).

We can also here compare similarities of the TICs in a heatmap. The retention times will however not be identical between different samples. Thus we *bin()* the chromatographic signal per sample along the retention time axis into bins of two seconds resulting in data with the same number of bins/data points. We can then calculate pairwise similarities between these data vectors using the `cor()` function and visualize the result using `pheatmap()`.

```
#' Total ion chromatogram
tic <- chromatogram(lcms1, aggregationFun = "sum") |>
  bin(binSize = 2)

#' Calculate similarity (Pearson correlation) between BPCs
ticmap <- do.call(cbind, lapply(tic, intensity)) |>
```

```
  cor()

rownames(ticmap) <- colnames(ticmap) <- sampleData(lcms1)$sample_name
ann <- data.frame(phenotype = sampleData(lcms1)[, "phenotype"])
rownames(ann) <- rownames(ticmap)

#' Plot heatmap
pheatmap(ticmap, annotation_col = ann,
         annotation_colors = list(phenotype = col_phenotype))
```

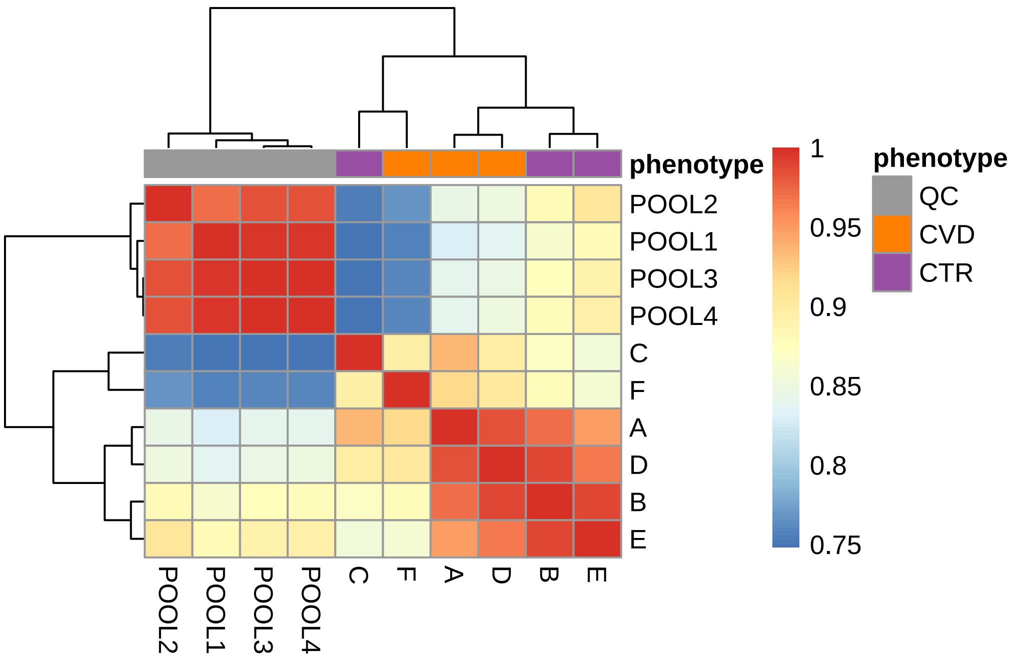

Figure 4: Heatmap of TIC similarities.

The heatmap above reinforces what our exploration of spectra data showed, which is a strong separation between the QC and study samples. This is important to bear in mind for later analyses.

Chromatographic Data Visualization: EICs

Throughout the entire process, it is crucial to have reference points within the dataset, such as well-known ions. Most experiments nowadays include internal standards (IS), and it was the case here. We strongly recommend using them for visualization throughout the entire analysis.

For this experiment, a set of 15 IS was spiked to all samples. After reviewing their respective chromatoraphic peaks, we selected two to guide this analysis process. However, we advise to plot and evaluate all the ions after each steps.

To illustrate this, we generate Extracted Ion Chromatograms (EIC) for these selected *test ions*. By restricting the MS data to intensities within a restricted, m/z range and a selected retention time window, EICs are expected to contain only signal from a single type of ion. The expected m/z and retention times for our set of IS was determined in a different experiment. Additionally, in cases where internal standards are not available, commonly present ions in the sample matrix can serve as suitable alternatives. Ideally, these compounds should be distributed across the entire retention time range of the experiment.

```r
#' Load our list of standard
intern_standard <- read.delim(
    paste0("https://github.com/rformassspectrometry/Metabonaut/raw/",
           "refs/heads/devel/vignettes/intern_standard_list.txt"))

# Extract EICs for the list
eic_is <- chromatogram(
    lcms1,
    rt = as.matrix(intern_standard[, c("rtmin", "rtmax")]),
    mz = as.matrix(intern_standard[, c("mzmin", "mzmax")]))

#' Add internal standard metadata
fData(eic_is)$mz <- intern_standard$mz
fData(eic_is)$rt <- intern_standard$RT
fData(eic_is)$name <- intern_standard$name
fData(eic_is)$abbreviation <- intern_standard$abbreviation
rownames(fData(eic_is)) <- intern_standard$abbreviation

fdata <- fData(eic_is)

#' Summary of IS information
fData(eic_is)[c("cystine_13C_15N", "methionine_13C_15N"),
              c("name", "mz", "rt")] |>
    as.data.frame() |>
    pandoc.table(style = "rmarkdown")
```

	name	mz	rt
**cystine__13C__15N**	L-Cystine (13C6, 99%; 15N2, 99%)	249	209

	name	mz	rt
**methionine_13C_15N**	L-Methionine (13C5, 99%; 15N, 99%)	156.1	161

Table 5: Internal standard list with respective m/z and expected retention time [s].

We below plot the EICs for isotope labeled cystine and methionine.

```
#' Extract the two IS from the chromatogram object.
eic_cystine <- eic_is["cystine_13C_15N"]
eic_met <- eic_is["methionine_13C_15N"]

#' plot both EIC
par(mfrow = c(1, 2), mar = c(4, 2, 2, 0.5))
plot(eic_cystine, main = fData(eic_cystine)$name, cex.axis = 0.8,
     cex.main = 0.8,
     col = paste0(col_sample, 80))
grid()
abline(v = fData(eic_cystine)$rt, col = "red", lty = 3)

plot(eic_met, main = fData(eic_met)$name, cex.axis = 0.8, cex.main = 0.8,
     col = paste0(col_sample, 80))
grid()
abline(v = fData(eic_met)$rt, col = "red", lty = 3)
legend("topright", col = col_phenotype, legend = names(col_phenotype),
       lty = 1, bty = "n")
```

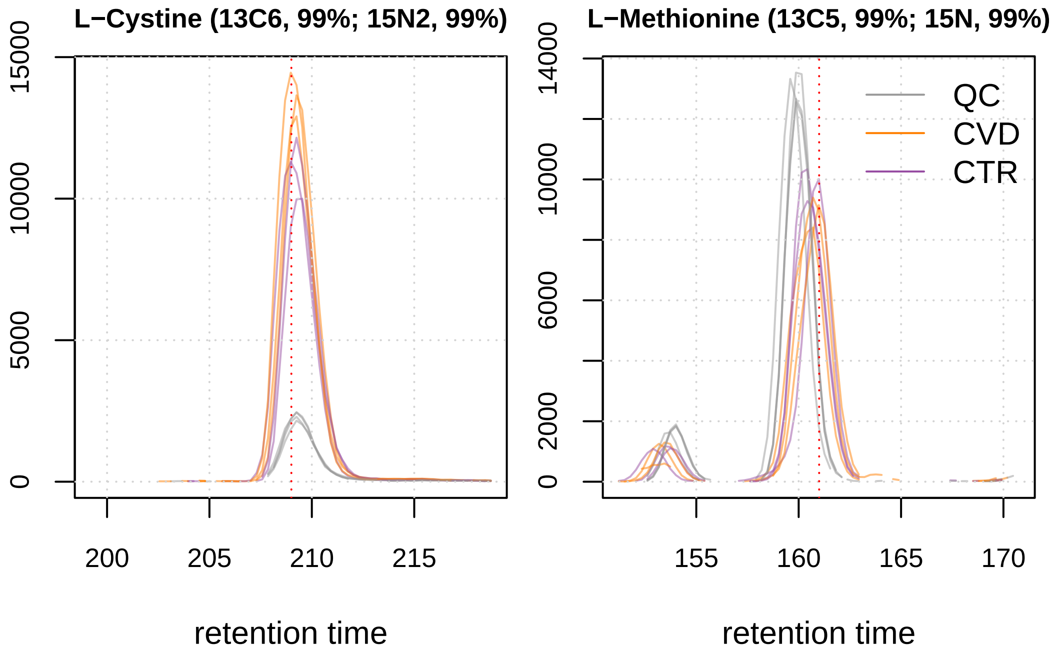

Figure 5: EIC of cystine and methionine.

We can observe a clear concentration difference between QCs and study samples for the isotope labeled cystine ion. Meanwhile, the labeled methionine internal standard exhibits a discernible signal amidst some noise and a noticeable retention time shift between samples.

Data preprocessing

Preprocessing stands as the inaugural step in the analysis of untargeted LC-MS. It is characterized by 3 main stages: **chromatographic peak detection**, **retention time shift correction** (alignment) and **correspondence** which results in features defined. The primary objective of preprocessing is the quantification of signals from ions measured in a sample, addressing any potential retention time drifts between samples, and ensuring alignment of quantified signals across samples within an experiment. The final result of LC-MS data preprocessing is a numeric matrix with abundances of quantified entities in the samples of the experiment.

Chromatographic peak detection

The initial preprocessing step involves detecting intensity peaks along the retention time axis, the so called *chromatographic peaks*. To achieve this, we employ the `findChromPeaks()` func-

tion within *xcms*. This function supports various algorithms for peak detection, which can be selected and configured with their respective parameter objects.

The preferred algorithm in this case, *CentWave*, utilizes continuous wavelet transformation (CWT)-based peak detection (Tautenhahn, Böttcher, and Neumann 2008). This method is known for its effectiveness in handling non-Gaussian shaped chromatographic peaks or peaks with varying retention time widths, which are commonly encountered in HILIC separations.

Below we apply the CentWave algorithm with its default settings on the EICs of cystine and methionine ions and evaluate the results: no chromatographic peaks are detected for the EICs with the default settings.

```
#' Use default Centwave parameter
param <- CentWaveParam()

#' Look at the default parameters
param
```

```
Object of class:  CentWaveParam
 Parameters:
 - ppm: [1] 25
 - peakwidth: [1] 20 50
 - snthresh: [1] 10
 - prefilter: [1]   3 100
 - mzCenterFun: [1] "wMean"
 - integrate: [1] 1
 - mzdiff: [1] -0.001
 - fitgauss: [1] FALSE
 - noise: [1] 0
 - verboseColumns: [1] FALSE
 - roiList: list()
 - firstBaselineCheck: [1] TRUE
 - roiScales: numeric(0)
 - extendLengthMSW: [1] FALSE
 - verboseBetaColumns: [1] FALSE
```

```
#' Evaluate for Cystine
cystine_test <- findChromPeaks(eic_cystine, param = param)
chromPeaks(cystine_test)
```

```
     mz mzmin mzmax rt rtmin rtmax into intb maxo sn row column
```

```
#' Evaluate for Methionine
met_test <- findChromPeaks(eic_met, param = param)
chromPeaks(met_test)
```

```
     mz mzmin mzmax rt rtmin rtmax into intb maxo sn row column
```

While *CentWave* is a highly performant algorithm, it requires to be customized to each dataset. This implies that the parameters should be fine-tuned based on the user's data. The example above serves as a clear motivation for users to familiarize themselves with the various parameters and the need to adapt them to a data set. We will discuss the main parameters that can be easily adjusted to suit the user's dataset:

- `peakwidth`: Specifies the minimal and maximal expected width of the peaks in the retention time dimension. Highly dependent on the chromatographic settings used.
- `ppm`: The maximal allowed difference of mass peaks' m/z values (in parts-per-million) in consecutive scans to consider them representing signal from the same ion.
- `integrate`: This parameter defines the integration method. Here, we primarily use `integrate = 2` because it integrates also signal of a chromatographic peak's tail and is considered more accurate by the developers.

To determine `peakwidth`, we recommend that users refer to previous EICs and estimate the range of peak widths they observe in their dataset. Ideally, examining multiple EICs should be the goal. For this dataset, the peak widths appear to be around 2 to 10 seconds. We do not advise choosing a range that is too wide or too narrow with the `peakwidth` parameter as it can lead to false positives or negatives.

To determine the `ppm`, a deeper analysis of the dataset is needed. It is clarified above that `ppm` depends on the instrument, but users should not necessarily input the vendor-advertised ppm. Here's how to determine it as accurately as possible:

The following steps involve generating a highly restricted MS area with a single mass peak per spectrum, representing the cystine ion. The m/z of these peaks is then extracted, their absolute difference calculated and finally expressed in ppm.

```
#' Restrict the data to signal from cystine in the first sample
cst <- lcms1[1L] |>
    spectra() |>
    filterRt(rt = c(208, 218)) |>
    filterMzRange(mz = fData(eic_cystine)["cystine_13C_15N",
                                          c("mzmin", "mzmax")])
```

```r
#' Show the number of peaks per m/z filtered spectra
lengths(cst)
```

```
 [1] 1 1 1 1 1 1 1 1 1 1 1 1 1 1 1 1 1 0 0 0 1 1 1 1 1 1 1 1 1 0 1 1 1 1 1 1
```

```r
#' Calculate the difference in m/z values between scans
mz_diff <- cst |>
    mz() |>
    unlist() |>
    diff() |>
    abs()

#' Express differences in ppm
range(mz_diff * 1e6 / mean(unlist(mz(cst))))
```

```
[1]  0.08829605 14.82188728
```

We therefore, choose a value close to the maximum within this range for parameter `ppm`, i.e., 15 ppm.

We can now perform the chromatographic peak detection with the adapted settings on our EICs. It is important to note that, to properly estimate background noise, sufficient data points outside the chromatographic peak need to be present. This is generally no problem if peak detection is performed on the full LC-MS data set, but for peak detection on EICs the retention time range of the EIC needs to be sufficiently wide. If the function fails to find a peak in an EIC, the initial troubleshooting step should be to increase this range. Additionally, the signal-to-noise threshold `snthresh` should be reduced for peak detection in EICs, because within the small retention time range, not enough signal is present to properly estimate the background noise. Finally, in case of too few MS1 data points per peaks, setting CentWave's advanced parameter `extendLengthMSW` to `TRUE` can help with peak detection.

```r
#' Parameters adapted for chromatographic peak detection on EICs.
param <- CentWaveParam(peakwidth = c(1, 8), ppm = 15, integrate = 2,
                       snthresh = 2)

#' Evaluate on the cystine ion
cystine_test <- findChromPeaks(eic_cystine, param = param)
chromPeaks(cystine_test)
```

```
            mz    mzmin    mzmax      rt   rtmin   rtmax      into      intb
mzmin 249.0453 249.0403 249.0503 209.251 207.577 212.878  4085.675  2911.376
mzmin 249.0453 249.0403 249.0503 209.251 206.182 213.995 24625.728 19074.407
mzmin 249.0453 249.0403 249.0503 209.252 207.020 214.274 19467.836 14594.041
mzmin 249.0453 249.0403 249.0503 209.251 207.577 212.041  4648.229  3202.617
mzmin 249.0453 249.0403 249.0503 208.974 206.184 213.159 23801.825 18126.978
mzmin 249.0453 249.0403 249.0503 209.250 207.018 213.714 25990.327 21036.768
mzmin 249.0453 249.0403 249.0503 209.252 207.857 212.879  4528.767  3259.039
mzmin 249.0453 249.0403 249.0503 209.252 207.299 213.995 23119.449 17274.140
mzmin 249.0453 249.0403 249.0503 208.972 206.740 212.878 28943.188 23436.119
mzmin 249.0453 249.0403 249.0503 209.252 207.578 213.437  4470.552  3065.402
           maxo sn row column
mzmin  2157.459  4   1      1
mzmin 12907.487  4   1      2
mzmin  9996.466  4   1      3
mzmin  2458.485  3   1      4
mzmin 11300.289  3   1      5
mzmin 13650.329  5   1      6
mzmin  2445.841  4   1      7
mzmin 12153.410  4   1      8
mzmin 14451.023  4   1      9
mzmin  2292.881  4   1     10
```

```
#' Evaluate on the methionine ion
met_test <- findChromPeaks(eic_met, param = param)
chromPeaks(met_test)
```

```
            mz    mzmin    mzmax      rt   rtmin   rtmax     into     intb
mzmin 156.0722 156.0672 156.0772 159.867 157.913 162.378 20026.61 14715.42
mzmin 156.0722 156.0672 156.0772 160.425 157.077 163.215 16827.76 11843.39
mzmin 156.0722 156.0672 156.0772 160.425 157.356 163.215 18262.45 12881.67
mzmin 156.0722 156.0672 156.0772 159.588 157.635 161.820 20987.72 15424.25
mzmin 156.0722 156.0672 156.0772 160.985 156.799 163.217 16601.72 11968.46
mzmin 156.0722 156.0672 156.0772 160.982 157.634 163.214 17243.24 12389.94
mzmin 156.0722 156.0672 156.0772 159.867 158.193 162.099 21120.10 16202.05
mzmin 156.0722 156.0672 156.0772 160.426 157.356 162.937 18937.40 13739.73
mzmin 156.0722 156.0672 156.0772 160.704 158.472 163.215 17882.21 12299.43
mzmin 156.0722 156.0672 156.0772 160.146 157.914 162.379 20275.80 14279.50
           maxo sn row column
mzmin 12555.601  4   1      1
mzmin  8407.699  3   1      2
mzmin  9283.375  3   1      3
```

```
mzmin 13327.811  4   1       4
mzmin 10012.396  4   1       5
mzmin  9150.079  4   1       6
mzmin 13531.844  3   1       7
mzmin 10336.000  3   1       8
mzmin  9395.548  3   1       9
mzmin 12669.821  3   1      10
```

With the customized parameters, a chromatographic peak was detected in each sample. Below, we use the `plot()` function on the EICs to visualize these results.

```
#' Plot test chromatogram
par(mfrow = c(1, 2))
plot(cystine_test, main = fData(cystine_test)$name,
     col = paste0(col_sample, 80),
     peakBg = paste0(col_sample, 40)[chromPeaks(cystine_test)[, "column"]],
     cex.main = 0.8, cex.axis = 0.8)
grid()

plot(met_test, main = fData(met_test)$name,
     col = paste0(col_sample, 80),
     peakBg = paste0(col_sample, 40)[chromPeaks(met_test)[, "column"]],
     cex.main = 0.8, cex.axis = 0.8)
grid()
legend("topright", col = col_phenotype,
       legend = names(col_phenotype), lty = 1, bty = "n")
```

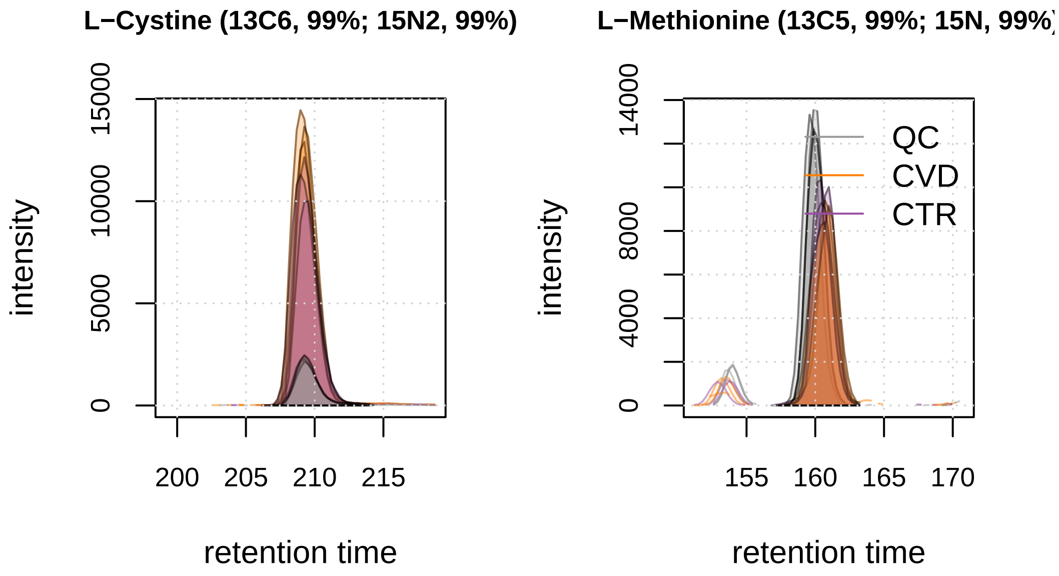

Figure 6: Chromatographic peak detection on EICs.

We can see a peak seems to be detected in each sample for both ions. This indicates that our custom settings are suitable for our dataset. We now proceed and apply them to the entire dataset, extracting EICs again for the same ions to evaluate and confirm that chromatographic peak detection worked as expected. Note:

- We revert the value for parameter `snthresh` to its default, because, as mentioned above, background noise estimation is more reliable when performed on the full data set.
- Parameter `chunkSize` of `findChromPeaks()` defines the number of data files that are loaded into memory and processed simultaneously. This parameter thus allows to fine-tune the memory demand as well as performance of the chromatographic peak detection step. This should correspond to our initial set up of parallel processing above.

A note on parameter `snthresh`: In general, using low values (`snthresh` 1 to 5) will result in a considerably higher number of detected chromatographic peaks, will however also pick noisy signal. Higher values (above 10) will result in higher quality peaks, but might miss low abundance signals. The selection of `snthresh` thus bases on a trade-off between restricting to detect only reliable signal or to discover also low abundant, but noisy data.

```
#' Using the same settings, but with default snthresh
param <- CentWaveParam(peakwidth = c(1, 8), ppm = 15, integrate = 2)
lcms1 <- findChromPeaks(lcms1, param = param, chunkSize = 2)
```

```r
#' Update EIC internal standard object
eics_is_noprocess <- eic_is
eic_is <- chromatogram(lcms1,,
                       rt = as.matrix(intern_standard[, c("rtmin", "rtmax")]),
                       mz = as.matrix(intern_standard[, c("mzmin", "mzmax")]))
```

```
Extracting chromatographic data
```

```
Processing chromatographic peaks
```

```r
fData(eic_is) <- fData(eics_is_noprocess)
```

Below we plot the EICs of the two selected internal standards to evaluate the chromatographic peak detection results.

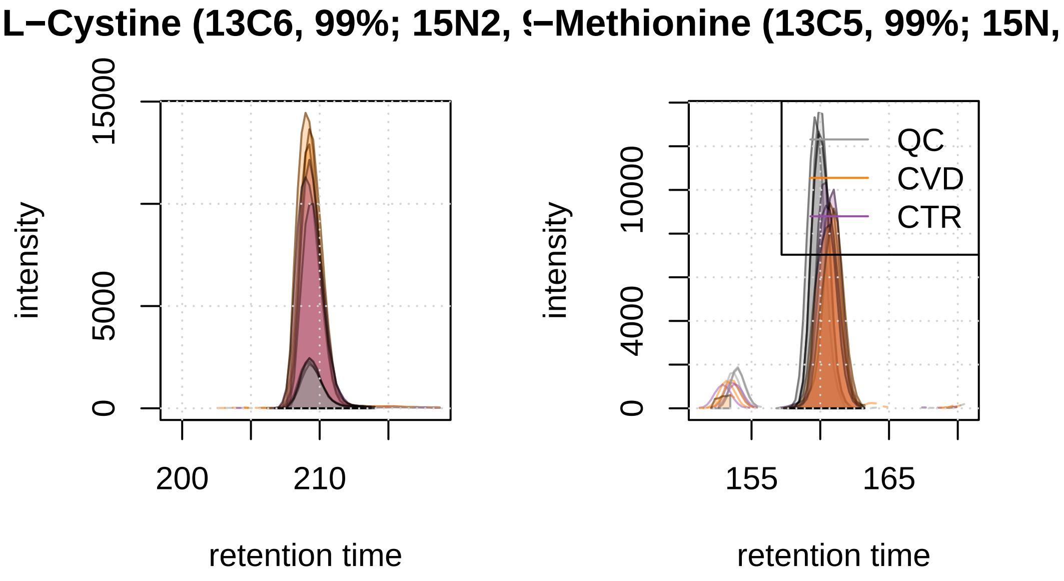

Figure 7: Chromatographic peak detection on EICs after processing.

Peaks seem to have been detected properly in all samples for both ions. This indicates that the peak detection process over the entire dataset was successful.

Refine identified chromatographic peaks

The identification of chromatographic peaks using the *CentWave* algorithm can sometimes result in artifacts, such as overlapping or split peaks. To address this issue, the `refineChromPeaks()` function is utilized, in conjunction with `MergeNeighboringPeaksParam`, which aims at merging such split peaks.

Below we show some examples of *CentWave* peak detection artifacts. These examples are pre-selected to illustrate the necessity of the next step:

```
#' Extract m/z-rt regions for selected peaks
mz_rt <- cbind(rtmin = c(155.2120, 181.71800),
               rtmax = c(201.0710, 228.13500),
               mzmin = c(191.0288,  53.50964),
               mzmax = c(191.0527, 53.53183))

#' Extract the EICs
eics <- chromatogram(lcms1[3], rt = mz_rt[, c("rtmin", "rtmax")],
                     mz = mz_rt[, c("mzmin", "mzmax")])
#' Plot the EICs
plot(eics)
```

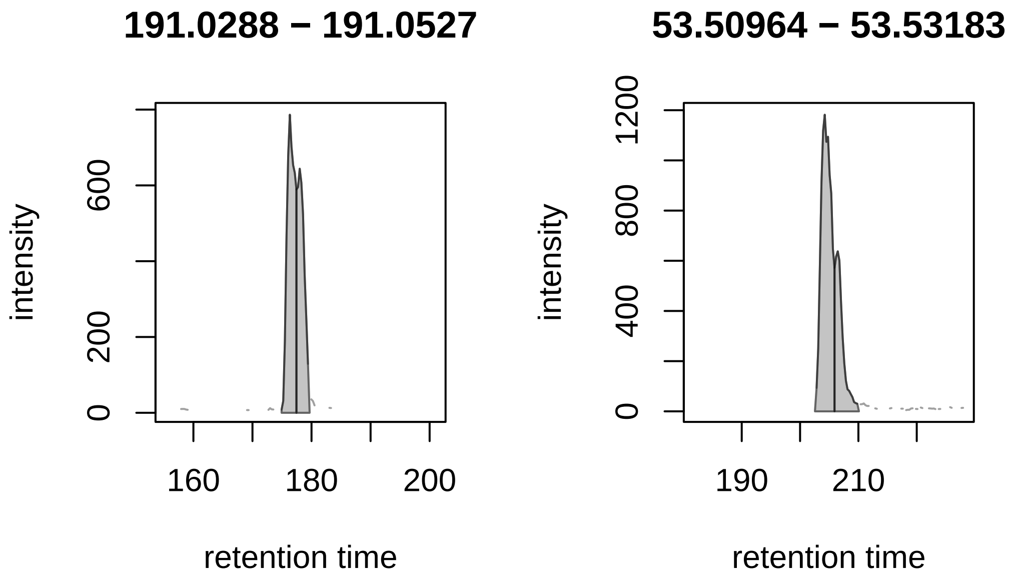

Figure 8: Examples of CentWave peak detection artifacts.

In both cases the signal presumably from a single type of ion was split into two separate chromatographic peaks (indicated by the vertical line). The `MergeNeigboringPeaksParam` allows to combine such split peaks. The parameters for this algorithm are defined below:

- `expandMz`and `expandRt`: Define which chromatographic peaks should be evaluated for merging.
  - `expandMz`: Suggested to be kept relatively small (here at 0.0015) to prevent the merging of isotopes.
  - `expandRt`: Usually set to approximately half the size of the average retention time width used for chromatographic peak detection (in this case, 2.5 seconds).
- `minProp`: Used to determine whether candidates will be merged. Chromatographic peaks with overlapping *m/z* ranges (expanded on each side by `expandMz`) and with a tail-to-head distance in the retention time dimension that is less than `2 * expandRt`, and for which the signal between them is higher than `minProp` of the apex intensity of the chromatographic peak with the lower intensity, are merged. Values for this parameter should not be too small to avoid merging closely co-eluting ions, such as isomers.

We test these settings below on the EICs with the split peaks.

```
#' set up the parameter
param <- MergeNeighboringPeaksParam(expandRt = 2.5, expandMz = 0.0015,
                                    minProp = 0.75)

#' Perform the peak refinement on the EICs
eics <- refineChromPeaks(eics, param = param)
plot(eics)
```

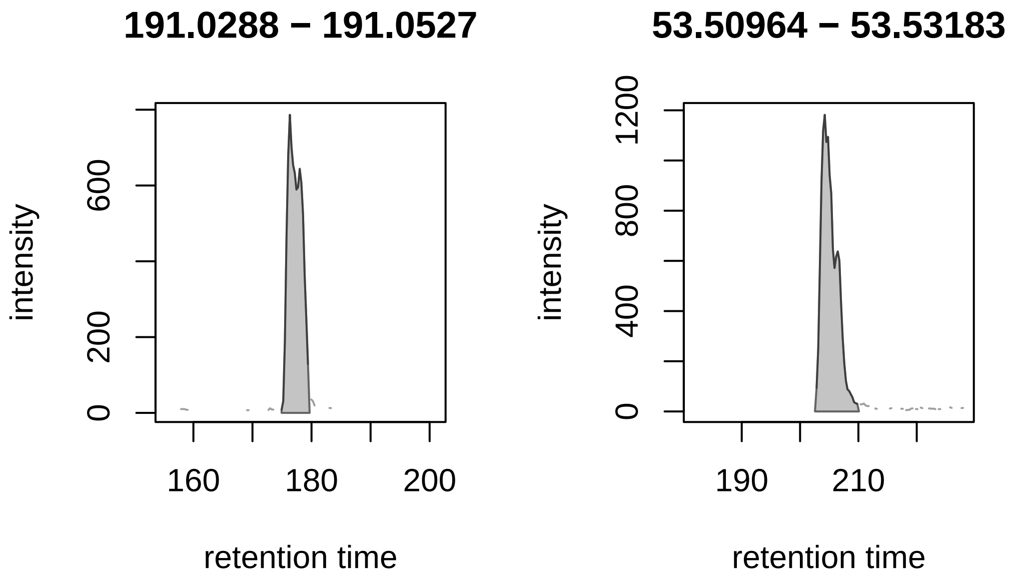

Figure 9: Examples of CentWave peak detection artifacts after merging.

We can observe that the artificially split peaks have been appropriately merged. Therefore, we next apply these settings to the entire dataset. After peak merging, column `"merged"` in the result object's `chromPeakData()` data frame can be used to evaluate which chromatographic peaks in the result represent signal from merged, or from the originally identified chromatographic peaks.

```
#' Apply on whole dataset
lcms1 <- refineChromPeaks(lcms1, param = param, chunkSize = 5)
```

```
Reduced from 106714 to 89182 chromatographic peaks.
```

```
chromPeakData(lcms1)$merged |>
                        table()
```

```
FALSE  TRUE
79908  9274
```

Before proceeding with the next preprocessing step it is generally suggested to evaluate the results of the chromatographic peak detection on EICs of e.g. internal standards or other

compounds/ions known to be present in the samples. Here we update our EICs objects for the internal standards with the results of the chromatographic peak detection and refinement.

```
eics_is_chrompeaks <- eic_is

eic_is <- chromatogram(lcms1,
                       rt = as.matrix(intern_standard[, c("rtmin", "rtmax")]),
                       mz = as.matrix(intern_standard[, c("mzmin", "mzmax")]))
fData(eic_is) <- fData(eics_is_chrompeaks)

eic_cystine <- eic_is["cystine_13C_15N", ]
eic_met <- eic_is["methionine_13C_15N", ]
```

Additionally, evaluating and comparing the number of identified chromatographic peaks in all samples of a data set can help spotting potentially problematic samples. Below we count the number of chromatographic peaks per sample and show these numbers in a table.

```
#' Count the number of peaks per sample and summarize them in a table.
data.frame(sample_name = sampleData(lcms1)$sample_name,
           peak_count = as.integer(table(chromPeaks(lcms1)[, "sample"]))) |>
  t() |>
  pandoc.table(style = "rmarkdown")
```

**sample_name**	POOL1	A	B	POOL2	C	D	POOL3
**peak_count**	9287	8986	8738	9193	8351	8778	9211

Table 6: Table continues below

**sample_name**	E	F	POOL4
**peak_count**	8787	8515	9336

Samples and number of identified chromatographic peaks.

A similar number of chromatographic peaks was identified within the various samples of the data set.

Additional options to evaluate the results of the chromatographic peak detection can be implemented using the `plotChromPeaks()` function or by summarizing the results using base R commands.

Retention time alignment

Despite using the same chromatographic settings and conditions retention time shifts are unavoidable. Indeed, the performance of the instrument can change over time, for example due to small variations in environmental conditions, such as temperature and pressure. These shifts will be generally small if samples are measured within the same batch/measurement run, but can be considerable if data of an experiment was acquired across a longer time period.

To evaluate the presence of a shift we extract and plot below the BPC from the QC samples.

```
#' Get QC samples
QC_samples <- sampleData(lcms1)$phenotype == "QC"

#' extract BPC
lcms1[QC_samples] |>
    chromatogram(aggregationFun = "max", chromPeaks = "none") |>
    plot(col = col_phenotype["QC"], main = "BPC of QC samples") |>
    grid()
```

```
Extracting chromatographic data
```

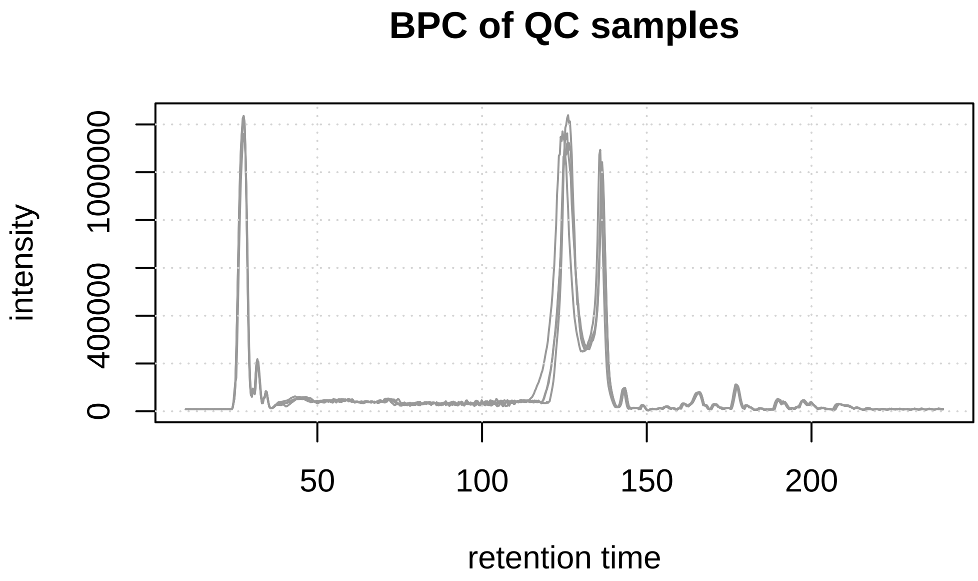

Figure 10: BPC of QC samples.

These QC samples representing the same sample (pool) were measured at regular intervals during the measurement run of the experiment and were all measured on the same day. Still, small shifts can be observed, especially in the region between 100 and 150 seconds. To facilitate proper correspondence of signals across samples (and hence definition of the LC-MS features), it is essential to minimize these differences in retention times.

Theoretically, we proceed in two steps: first we select only the QC samples of our dataset and do a first alignment on these, using the so-called *anchor peaks*. In this way we can assume a linear shift in time, since we are always measuring the same sample in different regular time intervals. Despite having external QCs in our data set, we still use the subset-based alignment assuming retention time shifts to be independent of the different sample matrix (human serum or plasma) and instead are mostly instrument-dependent. Note that it would also be possible to manually specify anchor peaks, respectively their retention times or to align a data set against an external, reference, data set. More information is provided in the vignettes of the *xcms* package.

After calculating how much to adjust the retention time in these samples, we apply this shift also on the study samples.

In *xcms*, retention time alignment can be performed using the `adjustRtime()` function with an alignment algorithm. For this example we use the *PeakGroups* method (Smith et al. 2006) that performs the alignment by minimizing differences in retention times of a set of *anchor peaks* in the different samples. This method requires an initial correspondence analysis to match/group chromatographic peaks across samples from which the algorithm then selects the anchor peaks for the alignment.

For the initial correspondence, we use the *PeakDensity* approach (Smith et al. 2006) that groups chromatographic peaks with similar *m/z* and retention time into LC-MS features. The parameters for this algorithm, that can be configured using the `PeakDensityParam` object, are `sampleGroups`, `minFraction`, `binSize`, `ppm` and `bw`.

`binSize`, `ppm` and `bw` allow to specify how similar the chromatographic peaks' *m/z* and retention time values need to be to consider them for grouping into a feature.

- `binSize` and `ppm` define the required similarity of *m/z* values. Within each *m/z* bin (defined by `binSize` and `ppm`) areas along the retention time axis with a high chromatographic peak density (considering all peaks in all samples) are identified, and chromatographic peaks within these regions are considered for grouping into a feature.
- High density areas are identified using the base R `density()` function, for which `bw` is a parameter: higher values define wider retention time areas, lower values require chromatographic peaks to have more similar retention times. This parameter can be seen as the black line on the plot below, corresponding to the smoothness of the density curve.

Whether such candidate peaks get grouped into a feature depends also on parameters `sampleGroups` and `minFraction`:

- `sampleGroups` should provide, for each sample, the sample group it belongs to.
- `minFraction` is expected to be a value between 0 and 1 defining the proportion of samples within at least one of the sample groups (defined with `sampleGroups`) in which a chromatographic peaks was detected to group them into a feature.

For the initial correspondence, parameters don't need to be fully optimized. Selection of dataset-specific parameter values is described in more detail in the next section. For our dataset, we use small values for `binSize` and `ppm` and, importantly, also for parameter `bw`, since for our data set an ultra high performance (UHP) LC setup was used. For `minFraction` we use a high value (0.9) to ensure only features are defined for chromatographic peaks present in almost all samples of one sample group (which can then be used as anchor peaks for the actual alignment). We will base the alignment later on QC samples only and hence define for `sampleGroups` a binary variable grouping samples either into a study, or QC group.

```
# Initial correspondence analysis
param <- PeakDensityParam(sampleGroups = sampleData(lcms1)$phenotype == "QC",
                          minFraction = 0.9,
                          binSize = 0.01, ppm = 10,
                          bw = 2)
lcms1 <- groupChromPeaks(lcms1, param = param)

plotChromPeakDensity(
    eic_cystine, param = param,
    col = paste0(col_sample, "80"),
    peakCol = col_sample[chromPeaks(eic_cystine)[, "sample"]],
    peakBg = paste0(col_sample[chromPeaks(eic_cystine)[, "sample"]], 20),
    peakPch = 16)
```

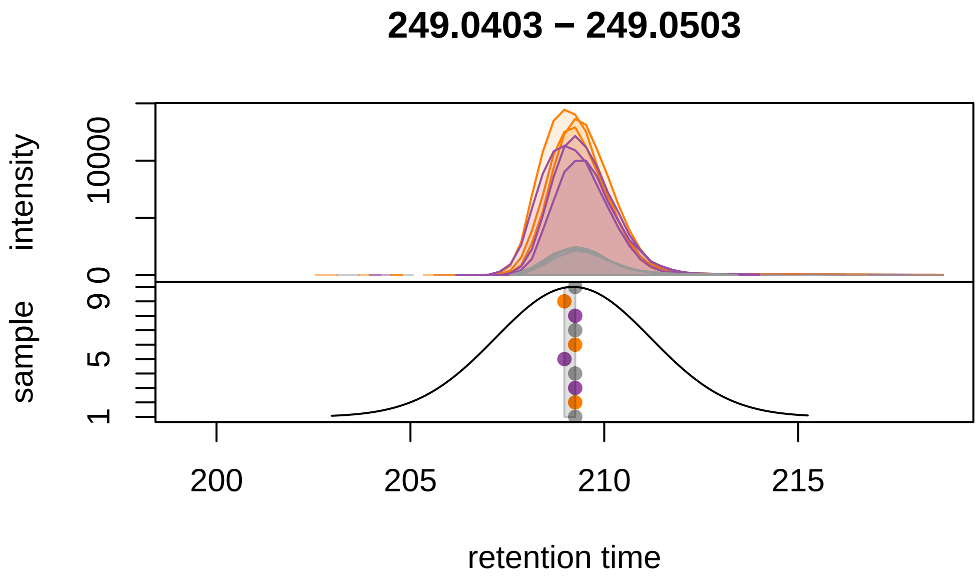

Figure 11: Initial correspondence analysis.

PeakGroups-based alignment can next be performed using the `adjustRtime()` function with a `PeakGroupsParam` parameter object. The parameters for this algorithm are:

- `subsetAdjust` and `subset`: Allows for subset alignment. Here we base the retention time alignment on the QC samples, i.e., retention time shifts will be estimated based on these repeatedly measured samples. The resulting adjustment is then applied to the entire data. For data sets in which QC samples (e.g. sample pools) are measured repeatedly, we strongly suggest to use this method. Note also that for subset-based alignment the samples should be ordered by injection index (i.e., in the order in which they were measured during the measurement run).
- `minFraction`: A value between 0 and 1 defining the proportion of samples (of the full data set, or the data subset defined with `subset`) in which a chromatographic peak has to be identified to use it as *anchor peak*. This is in contrast to the `PeakDensityParam` where this parameter was used to define a proportion within a sample group.
- `span`: The *PeakGroups* method allows, depending on the data, to adjust regions along the retention time axis differently. To enable such local alignments the *LOESS* function is used and this parameter defines the degree of smoothing of this function. Generally, values between 0.4 and 0.6 are used, however, it is suggested to evaluate alignment results and eventually adapt parameters if the result was not satisfactory.

Below we perform the alignment of our data set based on retention times of anchor peaks defined in the subset of QC samples.

```
#' Define parameters of choice
subset <- which(sampleData(lcms1)$phenotype == "QC")
param <- PeakGroupsParam(minFraction = 0.9, extraPeaks = 50, span = 0.5,
                         subsetAdjust = "average",
                         subset = subset)

#' Perform the alignment
lcms1 <- adjustRtime(lcms1, param = param)
```

Alignment adjusted the retention times of all spectra in the data set, as well as the retention times of all identified chromatographic peaks.

Once the alignment has been performed, the user should evaluate the results using the `plotAdjustedRtime()` function. This function visualizes the difference between adjusted and raw retention time for each sample on the y-axis along the adjusted retention time on the x-axis. Dot points represent the position of the used anchor peak along the retention time axis. For optimal alignment in all areas along the retention time axis, these anchor peaks should be scattered all over the retention time dimension.

```
#' Visualize alignment results
plotAdjustedRtime(lcms1, col = paste0(col_sample, 80), peakGroupsPch = 1)
grid()
legend("topright", col = col_phenotype,
       legend = names(col_phenotype), lty = 1, bty = "n")
```

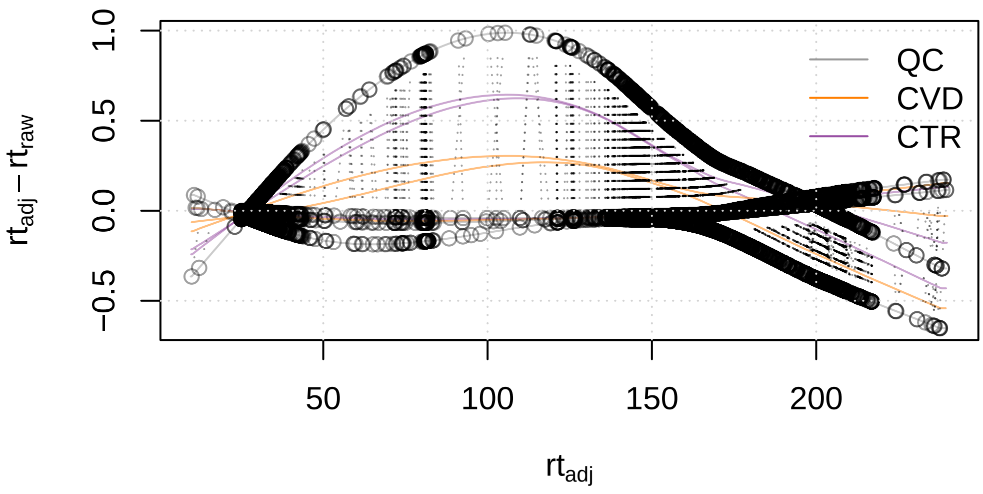

Figure 12: Retention time alignment results.

All samples from the present data set were measured within the same measurement run, resulting in small retention time shifts. Therefore, only little adjustments needed to be performed (shifts of at maximum 1 second as can be seen in the plot above). Generally, the magnitude of adjustment seen in such plots should match the expectation from the analyst.

We can also compare the BPC before and after alignment. To get the original data, i.e. the raw retention times, we can use the `dropAdjustedRtime()` function:

```
#' Get data before alignment
lcms1_raw <- dropAdjustedRtime(lcms1)

#' Apply the adjusted retention time to our dataset
lcms1 <- applyAdjustedRtime(lcms1)
```

```
#' Plot the BPC before and after alignment
par(mfrow = c(2, 1), mar = c(2, 1, 1, 0.5))
chromatogram(lcms1_raw, aggregationFun = "max", chromPeaks = "none") |>
    plot(main = "BPC before alignment", col = paste0(col_sample, 80))
```

`Extracting chromatographic data`

```
grid()
legend("topright", col = col_phenotype,
       legend = names(col_phenotype), lty = 1, bty = "n", horiz = TRUE)

chromatogram(lcms1, aggregationFun = "max", chromPeaks = "none") |>
    plot(main = "BPC after alignment",
         col = paste0(col_sample, 80))
```

```
Extracting chromatographic data
```

```
grid()
legend("topright", col = col_phenotype,
       legend = names(col_phenotype), lty = 1, bty = "n", horiz = TRUE)
```

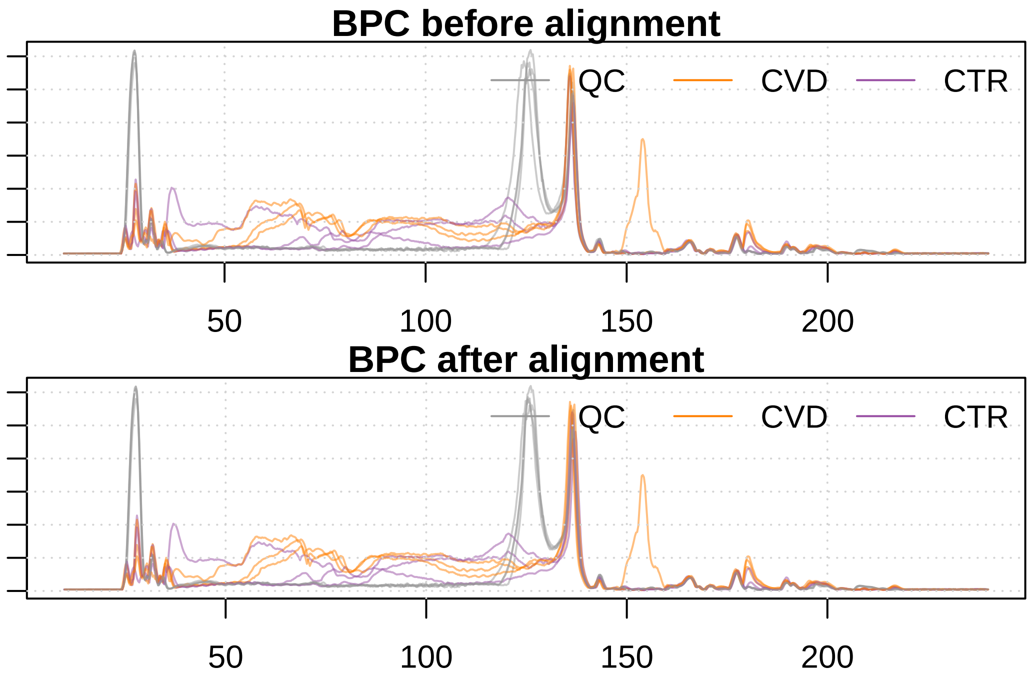

Figure 13: BPC before and after alignment.

The largest shift can be observed in the retention time range from 120 to 130s. Apart from that retention time range, only little changes can be observed.

We next evaluate the impact of the alignment on the EICs of the selected internal standards. We thus below first extract the ion chromatograms after alignment, and subsequently plot them.

```r
#' Extract the EICs for the test ions before alignment
old_eic_cystine <- eic_is["cystine_13C_15N"]
old_eic_met <- eic_is["methionine_13C_15N"]

#' Update the EICs
eic_is <- chromatogram(lcms1,
                       rt = as.matrix(intern_standard[, c("rtmin", "rtmax")]),
                       mz = as.matrix(intern_standard[, c("mzmin", "mzmax")]))
fData(eic_is) <- fdata

#' Extract the EICs for the test ions
eic_cystine <- eic_is["cystine_13C_15N"]
eic_met <- eic_is["methionine_13C_15N"]
```

```r
par(mfrow = c(2, 2), mar = c(4, 4.5, 2, 1))

plot(old_eic_cystine, main = "Cystine before alignment", peakType = "none",
     col = paste0(col_sample, 80))
grid()
abline(v = intern_standard["cystine_13C_15N", "RT"],
       col = "red", lty = 3)

plot(old_eic_met, main = "Methionine before alignment",
     peakType = "none", col = paste0(col_sample, 80))
grid()
abline(v = intern_standard["methionine_13C_15N", "RT"],
       col = "red", lty = 3)

plot(eic_cystine, main = "Cystine after alignment", peakType = "none",
     col = paste0(col_sample, 80))
grid()
abline(v = intern_standard["cystine_13C_15N", "RT"],
       col = "red", lty = 3)
legend("topright", col = col_phenotype,
       legend = names(col_phenotype), lty = 1, bty = "n")

plot(eic_met, main = "Methionine after alignment",
     peakType = "none", col = paste0(col_sample, 80))
grid()
abline(v = intern_standard["methionine_13C_15N", "RT"],
       col = "red", lty = 3)
```

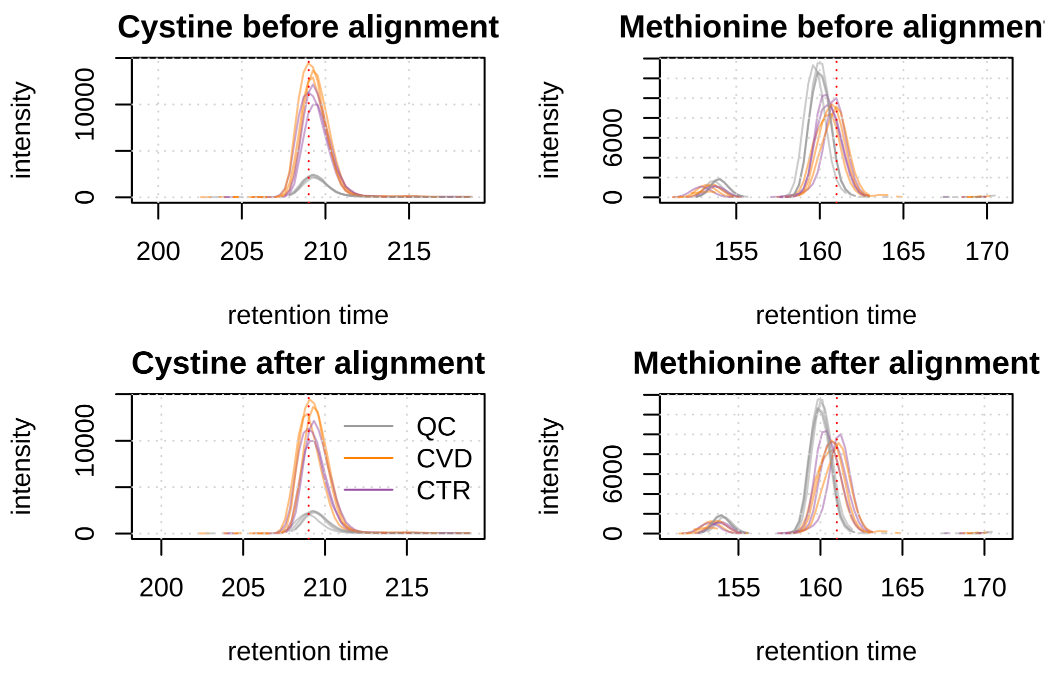

Figure 14: EICs of cystine and methionine before and after alignment.

The non-endogenous cystine ion was already well aligned so the difference is minimal. The methionine ion, however, shows a slight improvement in alignment.

In addition to a visual inspection of the results, we also evaluate the impact of the alignment by comparing the variance in retention times for internal standards before and after alignment. To this end, we first need to identify chromatographic peaks in each sample with an m/z and retention time close to the expected values for each internal standard. For this we use the `matchValues()` function from the *MetaboAnnotation* package (Rainer et al. 2022) using the `MzRtParam` method to identify all chromatographic peaks with similar m/z (+/- 50 ppm) and retention time (+/- 10 seconds) to the internal standard's values. With parameters `mzColname` and `rtColname` we specify the column names in the query (our IS) and target (chromatographic peaks) that contain the m/z and retention time values on which to match entities. We perform this matching separately for each sample. For each internal standard in every sample, we use the `filterMatches()` function and the `SingleMatchParam()` parameter to select the chromatographic peak with the highest intensity.

```
#' Extract the matrix with all chromatographic peaks and add a column
#' with the ID of the chromatographic peak
chrom_peaks <- chromPeaks(lcms1) |> as.data.frame()
chrom_peaks$peak_id <- rownames(chrom_peaks)
```

```r
#' Define the parameters for the matching and filtering of the matches
p_1 <- MzRtParam(ppm = 50, toleranceRt = 10)
p_2 <- SingleMatchParam(duplicates = "top_ranked", column = "target_maxo",
                        decreasing = TRUE)

#' Iterate over samples and identify for each the chromatographic peaks
#' with similar m/z and retention time than the onse from the internal
#' standard, and extract among them the ID of the peaks with the
#' highest intensity.
intern_standard_peaks <- lapply(seq_along(lcms1), function(i) {
    tmp <- chrom_peaks[chrom_peaks[, "sample"] == i, , drop = FALSE]
    mtch <- matchValues(intern_standard, tmp,
                        mzColname = c("mz", "mz"),
                        rtColname = c("RT", "rt"),
                        param = p_1)
    mtch <- filterMatches(mtch, p_2)
    mtch$target_peak_id
}) |>
    do.call(what = cbind)
```

We have now for each internal standard the ID of the chromatographic peak in each sample that most likely represents signal from that ion. We can now extract the retention times for these chromatographic peaks before and after alignment.

```r
#' Define the index of the selected chromatographic peaks in the
#' full chromPeaks matrix
idx <- match(intern_standard_peaks, rownames(chromPeaks(lcms1)))

#' Extract the raw retention times for these
rt_raw <- chromPeaks(lcms1_raw)[idx, "rt"] |>
    matrix(ncol = length(lcms1_raw))

#' Extract the adjusted retention times for these
rt_adj <- chromPeaks(lcms1)[idx, "rt"] |>
    matrix(ncol = length(lcms1_raw))
```

We can now evaluate the impact of the alignment on the retention times of internal standards across the full data set:

```r
list(all_raw = rowSds(rt_raw, na.rm = TRUE),
     all_adj = rowSds(rt_adj, na.rm = TRUE)
```

```
    ) |>
    vioplot()
grid()
```

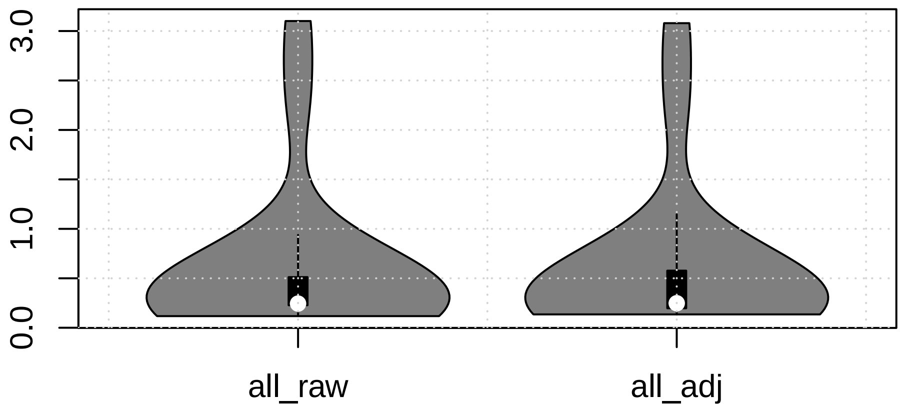

Figure 15: Retention time variation of internal standards before and after alignment.

On average, the variation in retention times of internal standards across samples was *very* slightly reduced by the alignment.

Correspondence

We briefly touched on the subject of correspondence before to determine anchor peaks for alignment. Generally, the goal of the correspondence analysis is to identify chromatographic peaks that originate from the same types of ions in all samples of an experiment and to group them into LC-MS *features*. At this point, proper configuration of parameter `bw` is crucial. Here we illustrate how sensible choices for this parameter's value can be made. We use below the `plotChromPeakDensity()` function to *simulate* a correspondence analysis with the default values for *PeakGroups* on the extracted ion chromatograms of our two selected isotope labeled ions. This plot shows the EIC in the top panel, and the apex position of chromatographic peaks in the different samples (y-axis), along retention time (x-axis) in the lower panel.

```r
#' Default parameter for the grouping and apply them to the test ions BPC
param <- PeakDensityParam(sampleGroups = sampleData(lcms1)$phenotype, bw = 30)

param
```

```
Object of class:  PeakDensityParam
 Parameters:
 - sampleGroups:  [1] "QC"  "CVD" "CTR" "QC"  "CTR" "CVD" "QC"  "CTR" "CVD" "QC"
 - bw: [1] 30
 - minFraction: [1] 0.5
 - minSamples: [1] 1
 - binSize: [1] 0.25
 - maxFeatures: [1] 50
 - ppm: [1] 0
```

```r
plotChromPeakDensity(
    eic_cystine, param = param,
    col = paste0(col_sample, "80"),
    peakCol = col_sample[chromPeaks(eic_cystine)[, "sample"]],
    peakBg = paste0(col_sample[chromPeaks(eic_cystine)[, "sample"]], 20),
    peakPch = 16)
```

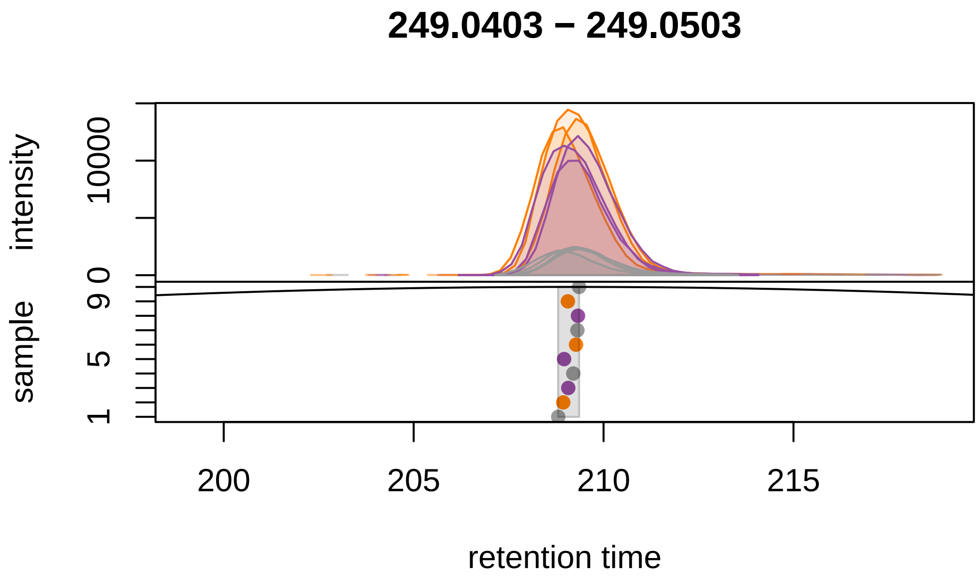

Figure 16: Initial correspondence analysis, Cystine.

```
plotChromPeakDensity(eic_met, param = param,
    col = paste0(col_sample, "80"),
    peakCol = col_sample[chromPeaks(eic_met)[, "sample"]],
    peakBg = paste0(col_sample[chromPeaks(eic_met)[, "sample"]], 20),
    peakPch = 16)
```

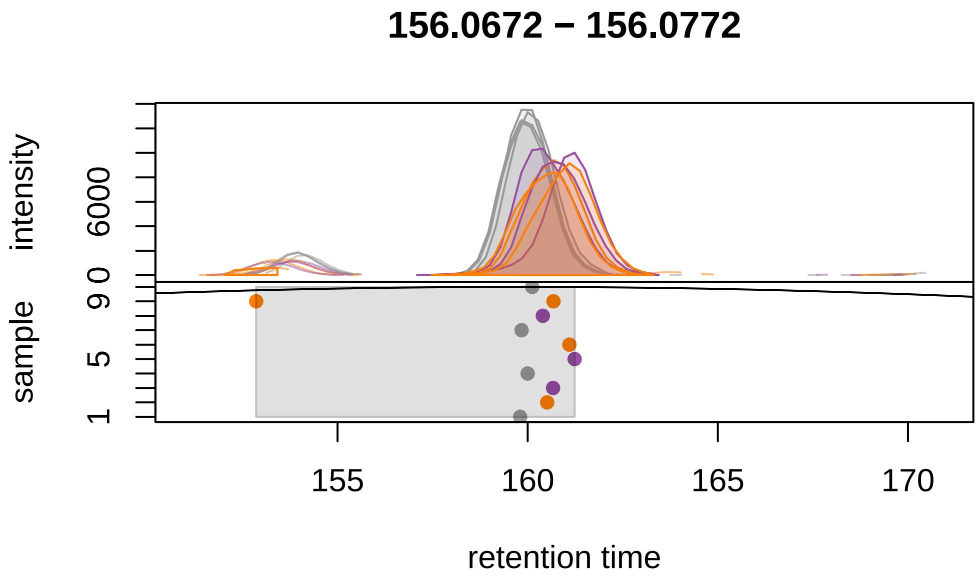

Figure 17: Initial correspondence analysis, Methionine.

Grouping of peaks depends on the smoothness of the previousl mentionned density curve that can be configured with the parameter `bw`. As seen above, the smoothness is too high to properly group our features. When looking at the default parameters, we can observe that indeed, the `bw` parameter is set to `bw = 30`, which is too high for modern UHPLC-MS setups. We reduce the value of this parameter to 1.8 and evaluate its impact.

```
#' Updating parameters
param <- PeakDensityParam(sampleGroups = sampleData(lcms1)$phenotype, bw = 1.8)

plotChromPeakDensity(
    eic_cystine, param = param,
    col = paste0(col_sample, "80"),
    peakCol = col_sample[chromPeaks(eic_cystine)[, "sample"]],
    peakBg = paste0(col_sample[chromPeaks(eic_cystine)[, "sample"]], 20),
    peakPch = 16)
```

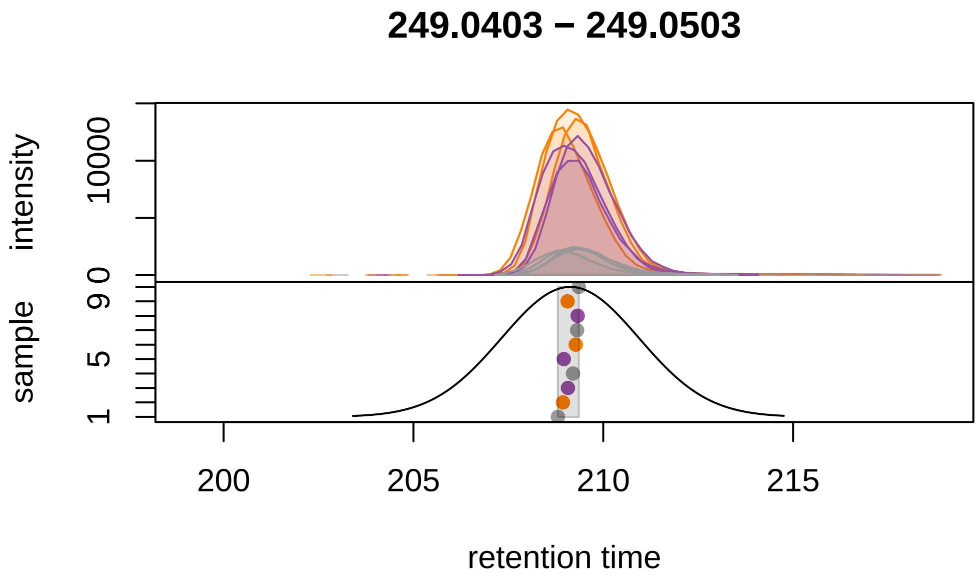

Figure 18: Correspondence analysis with optimized parameters, Cystine.

```
plotChromPeakDensity(eic_met, param = param,
    col = paste0(col_sample, "80"),
    peakCol = col_sample[chromPeaks(eic_met)[, "sample"]],
    peakBg = paste0(col_sample[chromPeaks(eic_met)[, "sample"]], 20),
    peakPch = 16)
```

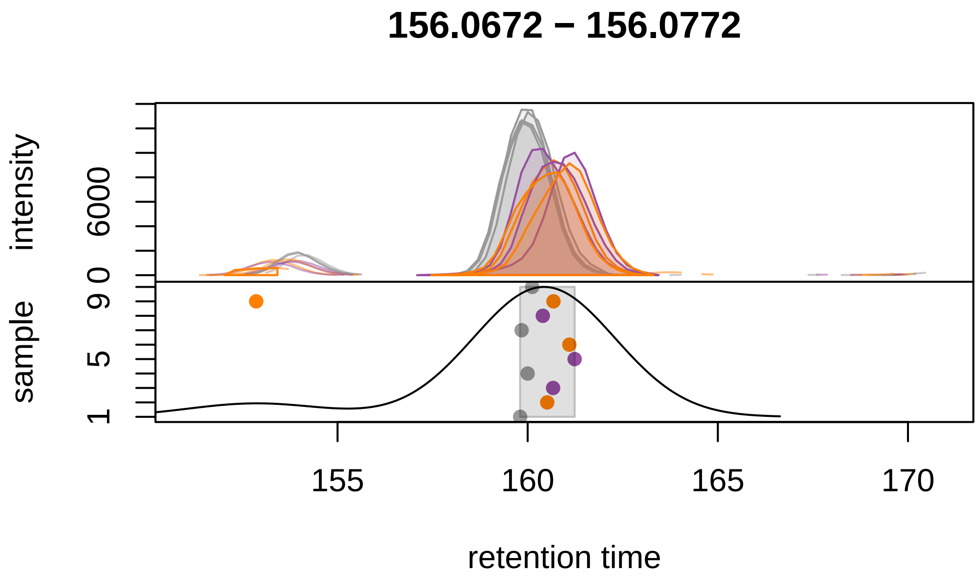

Figure 19: Correspondence analysis with optimized parameters, Methionine.

We can observe that the peaks are now grouped more accurately into a single feature for each test ion. The other important parameters optimized here are:

- `binsize`: Our data was generated on a high resolution MS instrument, thus we select a low value for this paramete.
- `ppm`: For TOF instruments, it is suggested to use a value for `ppm` larger than 0 to accommodate the higher measurement error of the instrument for larger m/z values.
- `minFraction`: We set it to `minFraction = 0.75`, hence defining features only if a chromatographic peak was identified in at least 75% of the samples of one of the sample groups.
- `sampleGroups`: We use the information available in our `sampleData`'s `"phenotype"` column. Note that this parameter also accept factors, and therefore more complicated groupings can be performed if wanted.

```
#' Define the settings for the param
param <- PeakDensityParam(sampleGroups = sampleData(lcms1)$phenotype,
                          minFraction = 0.75, binSize = 0.01, ppm = 10,
                          bw = 1.8)
```

```r
#' Apply to whole data
lcms1 <- groupChromPeaks(lcms1, param = param)
```

After correspondence analysis it is suggested to evaluate the results again for selected EICs. Below we extract signal for an m/z similar to that of the isotope labeled methionine for a larger retention time range. Importantly, to show the actual correspondence results, we set `simulate = FALSE` for the `plotChromPeakDensity()` function.

```r
#' Extract chromatogram for an m/z similar to the one of the labeled methionine
chr_test <- chromatogram(lcms1,
                         mz = as.matrix(intern_standard["methionine_13C_15N",
                                                       c("mzmin", "mzmax")]),
                         rt = c(145, 200),
                         aggregationFun = "max")
plotChromPeakDensity(
    chr_test, simulate = FALSE,
    col = paste0(col_sample, "80"),
    peakCol = col_sample[chromPeaks(chr_test)[, "sample"]],
    peakBg = paste0(col_sample[chromPeaks(chr_test)[, "sample"]], 20),
    peakPch = 16)
```

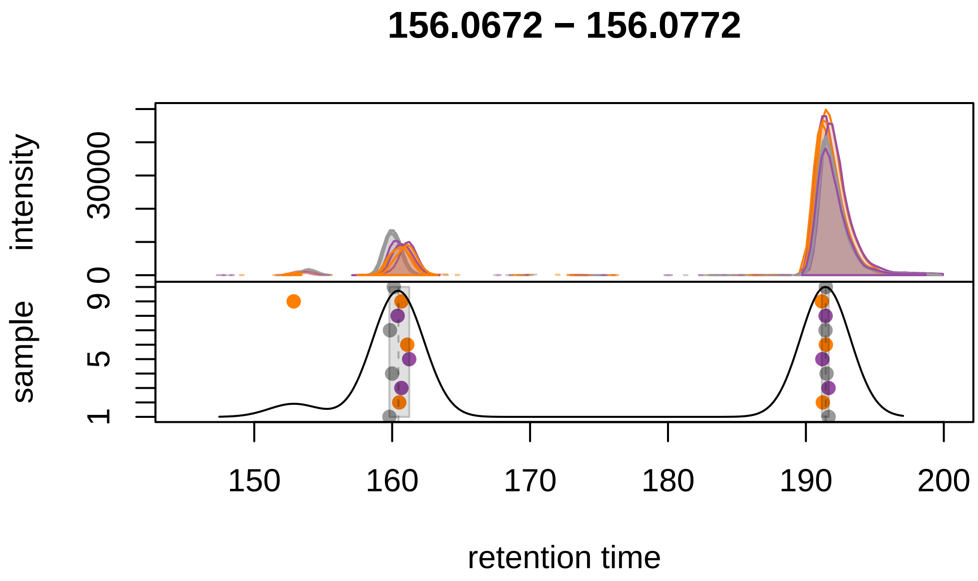

Figure 20: Correspondence analysis results, Methionine.

As hoped, signal from the two different ions are now grouped into separate features. Generally, correspondence results should be evaluated on more such extracted chromatograms.

Another interesting information to look a the the distribution of these features along the retention time axis.

```
# Bin features per RT slices
vc <- featureDefinitions(lcms1)$rtmed
breaks <- seq(0, max(vc, na.rm = TRUE) + 1, length.out = 15) |>
    round(0)
cuts <- cut(vc, breaks = breaks, include.lowest = TRUE)

table(cuts)
```

```
cuts
   [0,17]   (17,34]   (34,51]   (51,68]   (68,86]  (86,103] (103,120] (120,137]
       15      2608       649        24       132        11        15       105
(137,154] (154,171] (171,188] (188,205] (205,222] (222,240]
     1441      2202       410       869       554        33
```

The results from the correspondence analysis are now stored, along with the results from the other preprocessing steps, within our `XcmsExperiment` result object. The correspondence results, i.e., the definition of the LC-MS features, can be extracted using the `featureDefinitions()` function.

```
#' Definition of the features
featureDefinitions(lcms1) |>
  head()
```

```
          mzmed    mzmin    mzmax    rtmed    rtmin    rtmax npeaks CTR CVD QC
FT0001 50.98979 50.98949 50.99038 203.6001 203.1181 204.2331      8   1   3  4
FT0002 51.05904 51.05880 51.05941 191.1675 190.8787 191.5050      9   2   3  4
FT0003 51.98657 51.98631 51.98699 203.1467 202.6406 203.6710      7   0   3  4
FT0004 53.02036 53.02009 53.02043 203.2343 202.5652 204.0901     10   3   3  4
FT0005 53.52080 53.52051 53.52102 203.1936 202.8490 204.0901     10   3   3  4
FT0006 54.01007 54.00988 54.01015 159.2816 158.8499 159.4484      6   1   3  2
            peakidx ms_level
FT0001 7702, 16....        1
FT0002 7176, 16....        1
FT0003 7680, 17....        1
FT0004 7763, 17....        1
FT0005 8353, 17....        1
FT0006 5800, 15....        1
```

This data frame provides the average *m/z* and retention time (in columns `"mzmed"` and `"rtmed"`) that characterize a LC-MS feature. Column, `"peakidx"` contains the indices of all chromatographic peaks that were assigned to that feature. The actual abundances for these features, which represent also the final preprocessing results, can be extracted with the `featureValues()` function:

```
#' Extract feature abundances
featureValues(lcms1, method = "sum") |>
    head()
```

```
       MS_QC_POOL_1_POS.mzML MS_A_POS.mzML MS_B_POS.mzML MS_QC_POOL_2_POS.mzML
FT0001              421.6162      689.2422            NA              481.7436
FT0002              710.8078      875.9192            NA              693.6997
FT0003              445.5711      613.4410            NA              497.8866
FT0004            16994.5260    24605.7340     19766.707            17808.0933
FT0005             3284.2664     4526.0531      3521.822             3379.8909
FT0006            10681.7476    10009.6602            NA            10800.5449
       MS_C_POS.mzML MS_D_POS.mzML MS_QC_POOL_3_POS.mzML MS_E_POS.mzML
FT0001            NA      635.2732              439.6086      570.5849
FT0002      781.2416      648.4344              700.9716     1054.0207
FT0003            NA      634.9370              449.0933            NA
FT0004    22780.6683    22873.1061            16965.7762    23432.1252
FT0005     4396.0762     4317.7734             3270.5290     4533.8667
FT0006            NA     7296.4262                    NA     9236.9799
       MS_F_POS.mzML MS_QC_POOL_4_POS.mzML
FT0001      579.9360              437.0340
FT0002      534.4577              711.0361
FT0003      461.0465              232.1075
FT0004    22198.4607            16796.4497
FT0005     4161.0132             3142.2268
FT0006     6817.8785                    NA
```

We can note that a few features (e.g. F0003 and F0006) have missing values in some samples. This is expected to a certain degree as not in all samples features, respectively their ions, need to be present. We will address this in the next section.

Gap filling

The previously observed missing values (*NA*) could be attributed to various reasons. Although they might represent a genuinely missing value, indicating that an ion (feature) was truly not present in a particular sample, they could also be a result of a failure in the preceding

chromatographic peak detection step. It is crucial to be able to recover missing values of the latter category as much as possible to reduce the eventual need for data imputation. We next examine how prevalent missing values are in our present dataset:

```
#' Percentage of missing values
sum(is.na(featureValues(lcms1))) /
    length(featureValues(lcms1)) * 100
```

```
[1] 26.41597
```

We can observe a substantial number of missing values values in our dataset. Let's therefore delve into the process of *gap-filling*. We first evaluate some example features for which a chromatographic peak was only detected in some samples:

```
ftidx <- which(is.na(rowSums(featureValues(lcms1))))
fts <- rownames(featureDefinitions(lcms1))[ftidx]
farea <- featureArea(lcms1, features = fts[1:2])

chromatogram(lcms1[c(2, 3)],
             rt = farea[, c("rtmin", "rtmax")],
             mz = farea[, c("mzmin", "mzmax")]) |>
    plot(col = c("red", "blue"), lwd = 2)
```

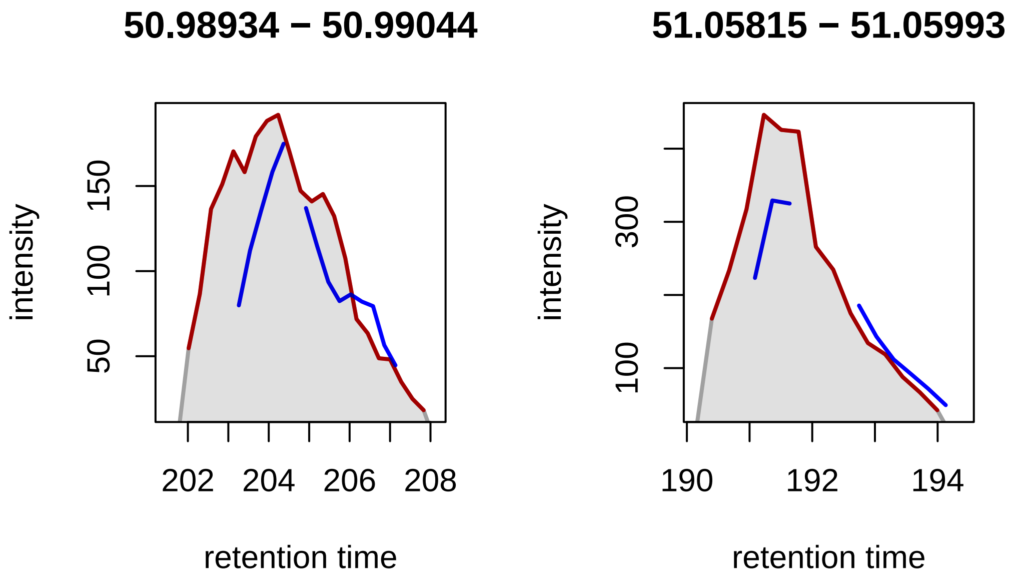

Figure 21: Examples of chromatographic peaks with missing values.

In both instances, a chromatographic peak was only identified in one of the two selected samples (red line), hence a missing value is reported for this feature in those particular samples (blue line). However, in both cases, signal was measured in both samples, thus, reporting a missing value would not be correct in this example. The signal for this feature is very low, which is the most likely reason peak detection failed. To rescue signal in such cases, the `fillChromPeaks()` function can be used with the `ChromPeakAreaParam` approach. This method defines an *m/z*-retention time area for each feature based on detected peaks, where the signal for the respective ion is expected. It then integrates all intensities within this area in samples that have missing values for that feature. This is then reported as feature abundance. Below we apply this method using the default parameters.

```
#' Fill in the missing values in the whole dataset
lcms1 <- fillChromPeaks(lcms1, param = ChromPeakAreaParam(), chunkSize = 5)

#' Percentage of missing values after gap-filling
sum(is.na(featureValues(lcms1))) /
    length(featureValues(lcms1)) * 100
```

```
[1] 5.155492
```

With `fillChromPeaks()` we could thus rescue most of the missing data in the data set. Note that, even if in a sample no ion would be present, in the worst case noise would be integrated, which is expected to be much lower than actual chromatographic peak signal. Let's look at our previously missing values again:

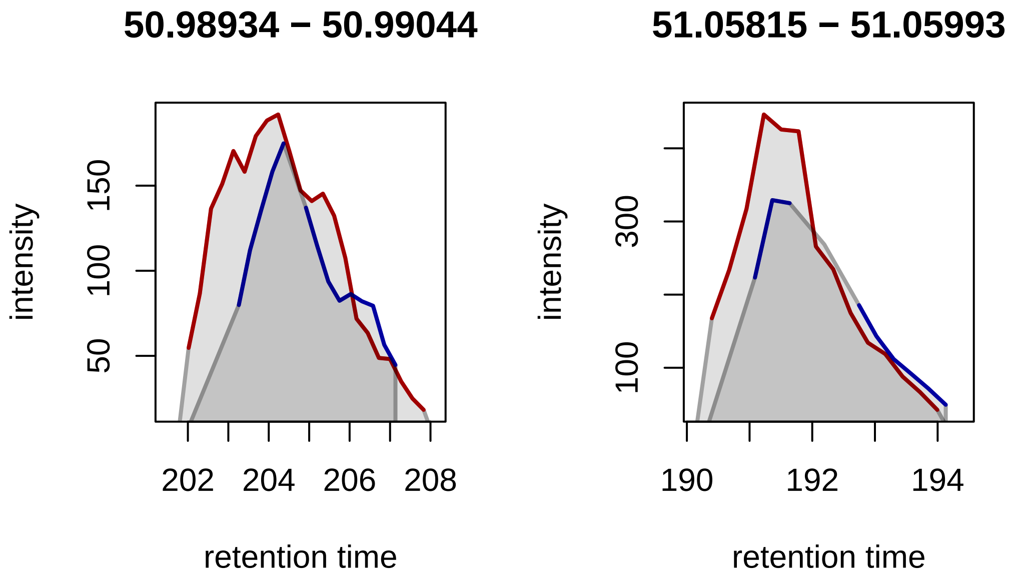

Figure 22: Examples of chromatographic peaks with missing values after gap-filling.

After gap-filling, also in the blue colored sample a chromatographic peak is present and its peak area would be reported as feature abundance for that sample.

To further assess the effectiveness of the gap-filling method for rescuing signals, we can also plot the average signal of features with at least one missing value against the average of filled-in signal. It is advisable to perform this analysis on repeatedly measured samples; in this case, our QC samples will be used.

For this, we extract:

- Feature values from detected chromatographic peaks by setting `filled = FALSE` in the `featuresValues()` call.
- The filled-in signal by first extracting both detected and gap-filled abundances and then replace the values for detected chromatographic peaks with `NA`.

Then, we calculate the row averages of both of these matrices and plot them against each other.

```r
#' Get only detected signal in QC samples
vals_detect <- featureValues(lcms1, filled = FALSE)[, QC_samples]

#' Get detected and filled-in signal
vals_filled <- featureValues(lcms1)[, QC_samples]

#' Replace detected signal with NA
vals_filled[!is.na(vals_detect)] <- NA

#' Identify features with at least one filled peak
has_filled <- is.na(rowSums(vals_detect))

#' Calculate row averages for features with missing values
avg_detect <- rowMeans(vals_detect[has_filled, ], na.rm = TRUE)
avg_filled <- rowMeans(vals_filled[has_filled, ], na.rm = TRUE)

#' Plot the values against each other (in log2 scale)
plot(log2(avg_detect), log2(avg_filled),
     xlim = range(log2(c(avg_detect, avg_filled)), na.rm = TRUE),
     ylim = range(log2(c(avg_detect, avg_filled)), na.rm = TRUE),
     pch = 21, bg = "#00000020", col = "#00000080")
grid()
abline(0, 1)
```

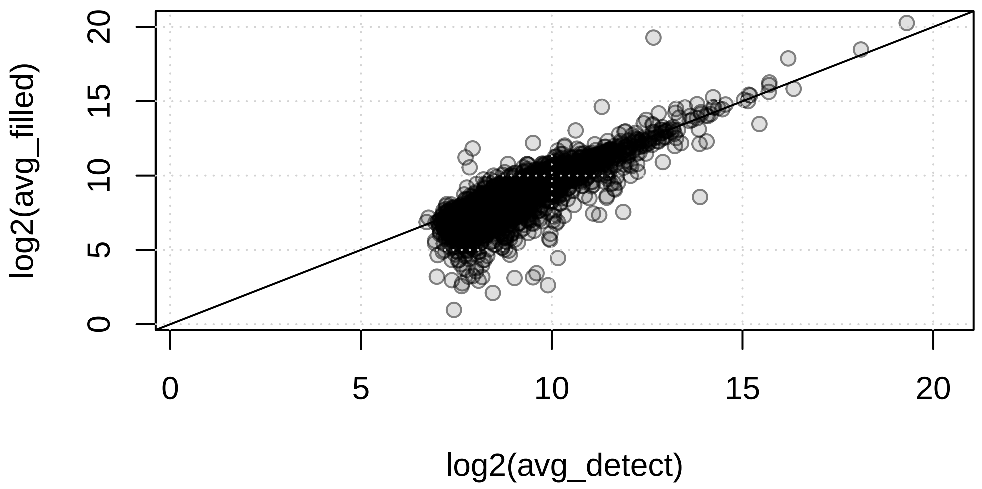

Figure 23: Detected vs. filled-in signal.

The detected (x-axis) and gap-filled (y-axis) values for QC samples are highly correlated. Especially for higher abundances, the agreement is very high, while for low intensities, as can be expected, differences are higher and trending to below the correlation line. Below we, in addition, fit a linear regression line to the data and summarize its results

```
#' fit a linear regression line to the data
l <- lm(log2(avg_filled) ~ log2(avg_detect))
summary(l)
```

```
Call:
lm(formula = log2(avg_filled) ~ log2(avg_detect))

Residuals:
    Min      1Q  Median      3Q     Max
-6.8176 -0.3807  0.1725  0.5492  6.7504

Coefficients:
                 Estimate Std. Error t value Pr(>|t|)
(Intercept)      -1.62359    0.11545  -14.06   <2e-16 ***
```

```
log2(avg_detect)  1.11763    0.01259   88.75   <2e-16 ***
---
Signif. codes:  0 '***' 0.001 '**' 0.01 '*' 0.05 '.' 0.1 ' ' 1

Residual standard error: 0.9366 on 2846 degrees of freedom
  (846 observations deleted due to missingness)
Multiple R-squared:  0.7346,    Adjusted R-squared:  0.7345
F-statistic:  7877 on 1 and 2846 DF,  p-value: < 2.2e-16
```

The linear regression line has a slope of 1.12 and an intercept of -1.62. This indicates that the filled-in signal is on average 1.12 times higher than the detected signal.

Filtering Features: Missing values

We now restrict the data set to features for which a chromatographic peak was detected in at least 2/3 of samples of at least one of the study samples groups. This ensures the statistical tests carried out later on the study samples are being performed on *reliable* signal. Also, with this filter we remove features that were mostly detected in QC samples, but not the study samples. Such filter can be performed with `filterFeatures()` function from the *xcms* package with the `PercentMissingFilter` setting. The parameters of this filer:

- `threshold`: defines the maximal acceptable percentage of samples with missing value(s) in at least one of the sample groups defined by parameter `f`.
- `f`: a factor defining the sample groups. By replacing the `"QC"` sample group with `NA` in parameter `f` we exclude the QC samples from the evaluation and consider only the study samples. With `threshold = 40` we keep only features for which a peak was detected in 2 out of the 3 samples in one of the sample groups.

To consider detected chromatographic peaks per sample, we apply the filter on the`"raw"` assay of our result object, that contains abundance values only for detected chromatographic peaks (prior gap-filling).

```r
#' Limit features to those with at least two detected peaks in one study group.
#' Setting the value for QC samples to NA excludes QC samples from the
#' calculation.
f <- sampleData(lcms1)$phenotype
f[f == "QC"] <- NA
f <- as.factor(f)
lcms1 <- filterFeatures(lcms1, PercentMissingFilter(f = f, threshold = 40))
```

```
344 features were removed
```

Preprocessing results

The final results of the LC-MS data preprocessing are stored within the `XcmsExperiment` object. This includes the identified chromatographic peaks, the alignment results, as well as the correspondence results. In addition, to guarantee reproducibility, this result object keeps track of all performed processing steps, including the individual parameter objects used to configure these. The `processHistory()` function returns a list of the various applied processing steps in chronological order. Below, we extract the information for the first step of the performed preprocessing.

```
#' Check first step of the process history
processHistory(lcms1)[[1]]
```

```
Object of class "XProcessHistory"
 type: Peak detection
 date: Wed Nov 12 07:34:02 2025
 info:
 fileIndex: 1,2,3,4,5,6,7,8,9,10
 Parameter class: CentWaveParam
 MS level(s) 1
```

The `processParam()` function could then be used to extract the actual parameter class used to configure this processing step.

The final result of the whole LC-MS data preprocessing is a two-dimensional matrix with abundances of the so-called LC-MS features in all samples. Note that at this stage of the analysis features are only characterized by their m/z and retention time and we don't have yet any information which metabolite a feature could represent.

As we have seen before, such feature matrix can be extracted with the `featureValues()` function and the corresponding feature characteristics (i.e., their m/z and retention time values) using the `featureDefinitions()` function. Thus, these two arrays could be extracted from the *xcms* result object and used/imported in other analysis packages for further processing. They could for example also be exported to tab delimited text files, and used in an external tool, or used, if also MS2 spectra were available, for feature-based molecular networking in the GNPS analysis environment (Nothias et al. 2020).

For further processing in R, if no reference or link to the raw MS data is required, it is suggested to extract the *xcms* preprocessing result using the `quantify()` function as a `SummarizedExperiment` object, Bioconductor's default container for data from biological assays/experiments. This simplifies integration with other Bioconductor analysis packages. The `quantify()` function takes the same parameters than the `featureValues()` function, thus, with the call below we extract a `SummarizedExperiment` with only the detected, but not gap-filled, feature abundances:

```r
#' Extract results as a SummarizedExperiment
res <- quantify(lcms1, method = "sum", filled = FALSE)
res
```

```
class: SummarizedExperiment
dim: 8724 10
metadata(7): '' '' ... '' ''
assays(1): raw
rownames(8724): FT0001 FT0002 ... FT9067 FT9068
rowData names(11): mzmed mzmin ... QC ms_level
colnames(10): MS_QC_POOL_1_POS.mzML MS_A_POS.mzML ... MS_F_POS.mzML
  MS_QC_POOL_4_POS.mzML
colData names(11): sample_name derived_spectra_data_file ... phenotype
  injection_index
```

Sample identifications from the *xcms* result's `sampleData()` are now available as `colData()` (column, sample annotations) and the `featureDefinitions()` as `rowData()` (row, feature annotations). The feature values have been added as the first `assay()` in the `SummarizedExperiment` and even the processing history is available in the object's `metadata()`. A `SummarizedExperiment` supports multiple assays, all being numeric matrices with the same dimensions. Below we thus add the detected and gap-filled feature abundances as an additional assay to the `SummarizedExperiment`.

```r
assays(res)$raw_filled <- featureValues(lcms1, method = "sum",
                                        filled = TRUE )

#' Different assay in the SummarizedExperiment object
assayNames(res)
```

```
[1] "raw"        "raw_filled"
```

Feature abundances can be extracted with the `assay()` function. Below we extract the first 6 lines of the detected and gap-filled feature abundances:

```r
assay(res, "raw_filled") |> head()
```

```
       MS_QC_POOL_1_POS.mzML MS_A_POS.mzML MS_B_POS.mzML MS_QC_POOL_2_POS.mzML
FT0001              421.6162      689.2422      411.3295              481.7436
FT0002              710.8078      875.9192      457.5920              693.6997
FT0003              445.5711      613.4410      277.5022              497.8866
```

```
FT0004            16994.5260    24605.7340    19766.7069              17808.0933
FT0005             3284.2664     4526.0531     3521.8221               3379.8909
FT0006            10681.7476    10009.6602     9599.9701              10800.5449
       MS_C_POS.mzML MS_D_POS.mzML MS_QC_POOL_3_POS.mzML MS_E_POS.mzML
FT0001      314.7567      635.2732              439.6086      570.5849
FT0002      781.2416      648.4344              700.9716     1054.0207
FT0003      425.3774      634.9370              449.0933      556.2544
FT0004    22780.6683    22873.1061            16965.7762    23432.1252
FT0005     4396.0762     4317.7734             3270.5290     4533.8667
FT0006     4792.2390     7296.4262             2382.1788     9236.9799
       MS_F_POS.mzML MS_QC_POOL_4_POS.mzML
FT0001      579.9360              437.0340
FT0002      534.4577              711.0361
FT0003      461.0465              232.1075
FT0004    22198.4607            16796.4497
FT0005     4161.0132             3142.2268
FT0006     6817.8785             6911.5439
```

An advantage, in addition to being a container for the full preprocessing results is also the possibility of an easy and intuitive creation of data subsets ensuring data integrity. It would for example be very easy to subset the full data to a selection of features and/or samples:

```r
res[1:14, 3:8]
```

```
class: SummarizedExperiment
dim: 14 6
metadata(7): '' '' ... '' ''
assays(2): raw raw_filled
rownames(14): FT0001 FT0002 ... FT0013 FT0014
rowData names(11): mzmed mzmin ... QC ms_level
colnames(6): MS_B_POS.mzML MS_QC_POOL_2_POS.mzML ...
  MS_QC_POOL_3_POS.mzML MS_E_POS.mzML
colData names(11): sample_name derived_spectra_data_file ... phenotype
  injection_index
```

Before moving to the next step of the analysis, it is advisable to save the preprocessing results. We have multiple format options to save into, and they can be found in the `MsIO` package documentation. Below we will save our `XcmsExperiment` object into a file format handled by the alabster framework, which ensures that our object can be easily read from other languages like Python and Javascript as well as loaded easily back into R.

```r
#' XcmsExperiment object:
saveMsObject(lcms1,
             AlabasterParam(path = file.path("objects/preprocessed_lcms1")))

#' Below we remove the processHistory of the res object to allow for export.
metadata(res) <- list()
saveObject(res, file.path("objects/preprocessed_res"))
```

Data normalization

After preprocessing, data normalization or scaling might need to be applied to remove any technical variances from the data. While simple approaches like median scaling can be implemented with a few lines of R code, more advanced normalization algorithms are available in packages such as Bioconductor's *preprocessCore*. The comprehensive workflow "Notame" also propose a very interesting normalization approach adaptable and scalable to the user dataset (Klåvus et al. 2020).

Generally, for LC-MS data, bias can be categorized into three main groups(Broadhurst et al. 2018):

- Variances introduced by sample collection and initial processing, which can include differences in sample amounts. This type of bias is expected to be sample-specific and affect all signals in a sample in the same way. Methods like median scaling, LOESS or quantiles normalization can adjust this bias.
- Signal drifts along measurement of samples from an experiment. Reasons for such drifts can be related to aging of the instrumentation used (columns, detector), but also to changes in metabolite abundances and characteristics due to reactions and modifications, such as oxidation. These changes are expected to affect more the samples measured later in a run rather than the ones measured at the beginning. For this reason, this bias can play a major role in large experiments bias can play a major role in large experiments measured over a long time range and is usually considered to affect individual metabolites (or metabolite groups) differently. For adjustment, moving average or linear regression-based approaches can be used. The latter can for example be performed using the `adjust_lm()` function from the *MetaboCoreUtils* package.
- Batch-related biases. These comprise any noise that is specific to a larger set of samples, which can be the set of samples measured in one LC-MS measurement run (i.e. from one analysis plate) or samples measured using a specific batch of reagents. This noise is assumed to affect all samples in one batch in the same way and linear modeling-based approaches can be used to adjust for this.

Unwanted variation can arise from various sources and is highly dependent on the experiment. Therefore, data normalization should be chosen carefully based on experimental design, statistical aims, and the balance of accuracy and precision achieved through the use of auxiliary information.

Sample preparation biases can be evaluated using internal standards, depending however also on when they were added to the sample mixes during sample processing. Repeated measurements of QC samples on the other hand allows to estimate and correct for LC-MS specific biases. Also, proper planning of an experiment, such as measurement of study samples in random order, can largely avoid biases introduced by most of the above mentioned sources of variance.

In this workflow we present some tools to assess data quality and evaluate need for normalization as well as options for normalization. For space reasons we are not able to provide solutions to adjust for all possible sources of variation.

Initial quality assessment

A principal component analysis (PCA) is a very helpful tool for an initial, unsupervised, visualization of the data that also provides insights into potential quality issues in the data. In order to apply a PCA to the measured feature abundances, we need however to impute (the still present) missing values. We assume that most of these missing values (after the gap-filling step) represent signal which is below detection limit. In such cases, missing values can be replaced with random values sampled from a uniform distribution, ranging from half of the smallest measured value to the smallest measured value for a specific feature. The uniform distribution is defined with two parameters (minimum and maximum) and all values between them have an equal probability of being selected.

Below we impute missing values with this approach and add the resulting data matrix as a new *assay* to our result object.

```
#' Impute missing values using an uniform distribution
na_unidis <- function(z) {
    na <- is.na(z)
    if (any(na)) {
        min = min(z, na.rm = TRUE)
        z[na] <- runif(sum(na), min = min/2, max = min)
    }
    z
}

#' Row-wise impute missing values and add the data as a new assay
```

```r
tmp <- apply(assay(res, "raw_filled"), MARGIN = 1, na_unidis)
assays(res)$raw_filled_imputed <- t(tmp)
```

Principal Component Analysis

PCA is a powerful tool for detecting biases in data. As a dimensionality reduction technique, it enables visualization of data in a lower-dimensional space. In the context of LC-MS data, PCA can be used to identify overall biases in batch, sample, injection index, etc. However, it is important to note that PCA is a linear method and may not be able to detect all biases in the data.

Before plotting the PCA, we apply a log2 transform, center and scale the data. The log2 transformation is applied to stabilize the variance while centering to remove dependency on absolute abundances.

```r
#' Log2 transform and scale data
vals <- assay(res, "raw_filled_imputed") |>
    log2() |>
    t() |>
    scale(center = TRUE, scale = TRUE)

#' Perform the PCA
pca_res <- prcomp(vals, scale = FALSE, center = FALSE)

#' Plot the results
vals_st <- cbind(vals, phenotype = res$phenotype)
pca_12 <- autoplot(pca_res, data = vals_st , colour = 'phenotype',scale = 0) +
    scale_color_manual(values = col_phenotype)
pca_34 <- autoplot(pca_res, data = vals_st, colour = 'phenotype',
                   x = 3, y = 4, scale = 0) +
    scale_color_manual(values = col_phenotype)
grid.arrange(pca_12, pca_34, ncol = 1)
```

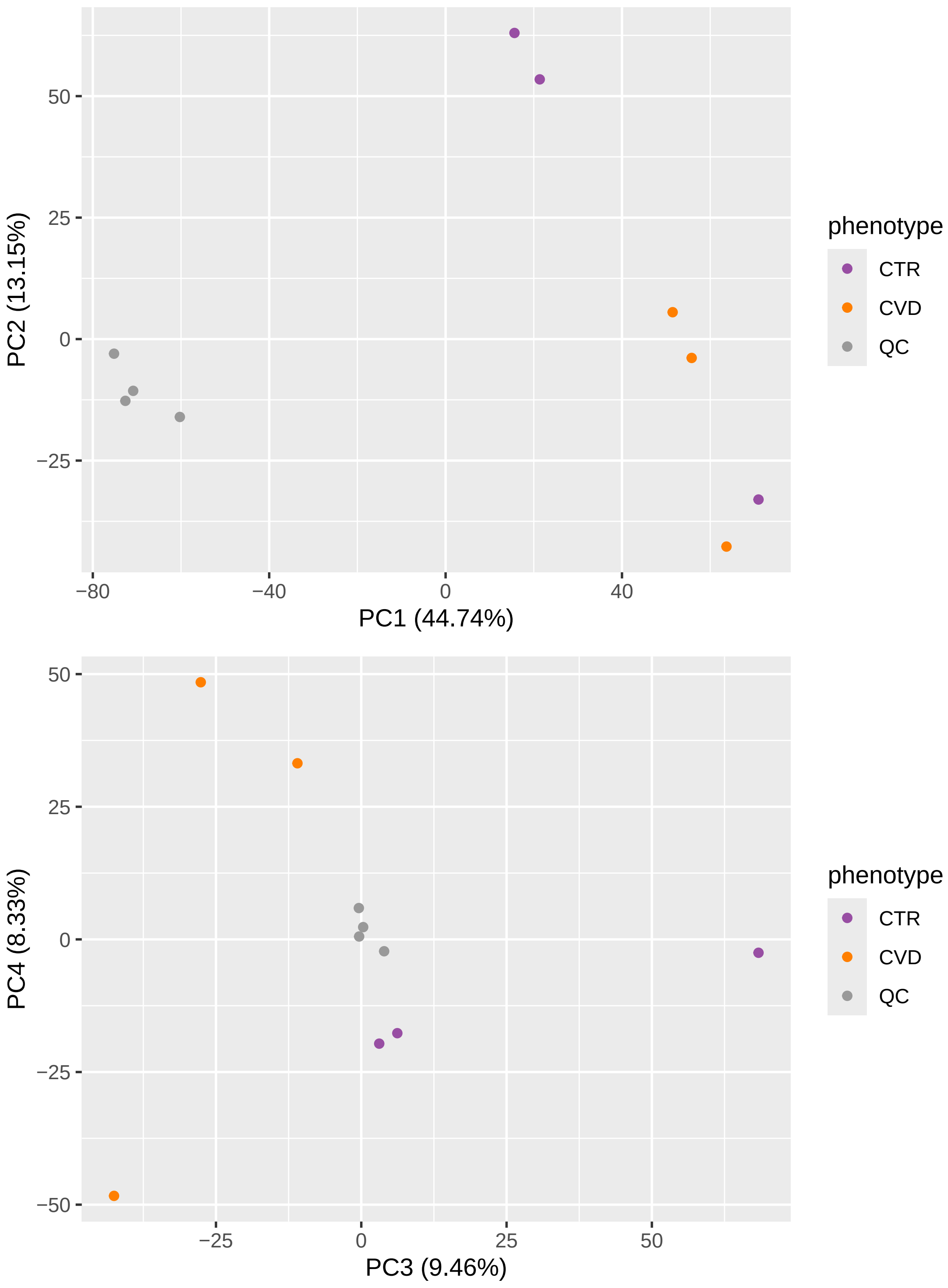

Figure 24: PCA of the data coloured by phenotypes.

The PCA above shows a clear separation of the study samples (plasma) from the QC samples (serum) on the first principal component (PC1). The separation based on phenotype is visible on the third principal component (PC3).

In some cases, it can be a better option to remove the imputed values and evaluate the PCA again. This is especially true if the imputed values are replacing a large proportion of the data.

Intensity evaluation

Global differences in feature abundances between samples (e.g. due to sample-specific biases) can be evaluated by plotting the distribution of the log2 transformed feature abundances using boxplots of violin plots. Below we show the number of detected chromatographic peaks per sample and the distribution of the log2 transformed feature abundances.

```
layout(mat = matrix(1:3, ncol = 1), height = c(0.2, 0.2, 0.8))

par(mar = c(0.2, 4.5, 0.2, 3))
barplot(apply(assay(res, "raw"), MARGIN = 2, function(x) sum(!is.na(x))),
        col = paste0(col_sample, 80), border = col_sample,
        ylab = "# detected peaks", xaxt = "n", space = 0.012)
grid(nx = NA, ny = NULL)
barplot(apply(assay(res, "raw_filled"), MARGIN = 2,
              function(x) sum(!is.na(x))),
        col = paste0(col_sample, 80), border = col_sample,
        ylab = "# detected + filled peaks", xaxt = "n",
        space = 0.012)
grid(nx = NA, ny = NULL)
vioplot(log2(assay(res, "raw_filled")), xaxt = "n",
        ylab = expression(log[2]~feature~abundance),
        col = paste0(col_sample, 80), border = col_sample)
points(colMedians(log2(assay(res, "raw_filled")), na.rm = TRUE),
       type = "b", pch = 1)
grid(nx = NA, ny = NULL)
legend("topright", col = col_phenotype,
       legend = names(col_phenotype), lty=1, lwd = 2, xpd = TRUE,
       ncol = 3, cex = 0.8,  bty = "n")
```

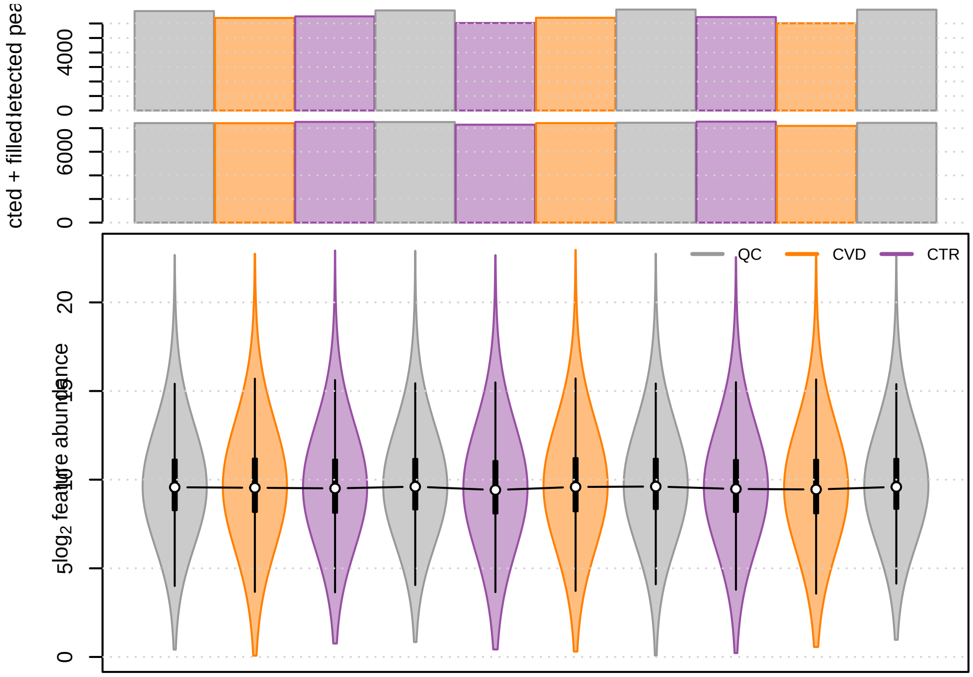

Figure 25: Number of detected peaks and feature abundances.

The upper part of the plot show that the gap filling steps allowed to rescue a substantial number of *NAs* and allowed us to have a more consistent number of feature values per sample. This consistency aligns with our asspumption that every sample should have a similar amount of features detected. Additionally we observe that, on average, the signal distribution from the individual samples is very similar.

An alternative way to evaluate differences in abundances between samples are *relative log abundance* (RLA) plots (De Livera et al. 2012). An RLA value is the abundance of a feature in a sample relative to the median abundance of the same feature across multiple samples. We can discriminate between *within group* and *across group* RLAs, depending whether the abundance is compared against samples within the same sample group or across all samples. Within group RLA plots assess the tightness of replicates within groups and should have a median close to zero and low variation around it. When used across groups, they allow to compare behavior between groups. Generally, between-sample differences can be easily spotted using RLA plots. Below we calculate and visualize within group RLA values using the `rowRla()` function from the *MsCoreUtils* package defining with parameter `f` the sample groups.

```
par(mfrow = c(1, 1), mar = c(3.5, 4.5, 2.5, 1))
boxplot(MsCoreUtils::rowRla(assay(res, "raw_filled"),
                            f = res$phenotype, transform = "log2"),
```

```
        cex = 0.5, pch = 16,
        col = paste0(col_sample, 80), ylab = "RLA",
        border = col_sample, boxwex = 1,
        outline = FALSE, xaxt = "n", main = "Relative log abundance",
        cex.main = 1)
axis(side = 1, at = seq_len(ncol(res)), labels = colData(res)$sample_name)
grid(nx = NA, ny = NULL)
abline(h = 0, lty=3, lwd = 1, col = "black")
legend("topright", col = col_phenotype,
       legend = names(col_phenotype), lty=1, lwd = 2, xpd = TRUE,
       ncol = 3, cex = 0.8,  bty = "n")
```

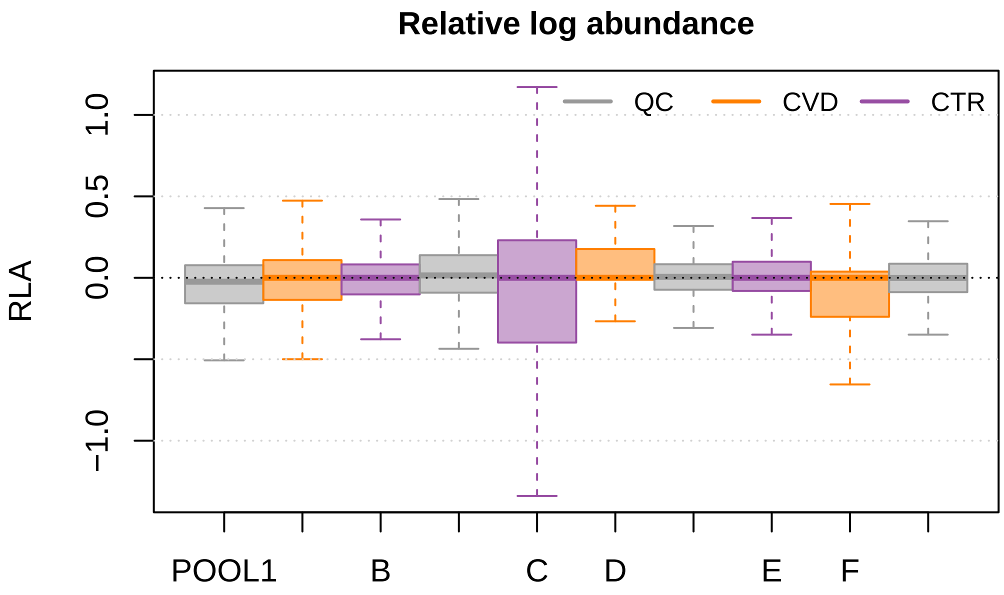

Figure 26: RLA plot for the raw data and filled data.

On the RLA plot above, we can observe that the medians for most samples are indeed centered around 0. Exception are two of the *CVD* samples. Thus, while the distribution of signals across samples is comparable, some differences seem to be present that require a between sample normalization.

Between sample normalisation

The previous RLA plot showed that the data had some biases that need to be corrected. Therefore, we will implement between-sample normalization using filled-in features. This process effectively mitigates variations influenced by technical issues, such as differences in sample preparation, processing and injection methods. In this instance, we will employ a commonly used technique known as median scaling (De Livera et al. 2012).

Median scaling

This method involves computing the median for each sample, followed by determining the median of these individual sample medians. This ensures consistent median values for each sample throughout the entire data set. Maintaining uniformity in the average total metabolite abundance across all samples is crucial for effective implementation.

This process aims to establish a shared baseline for the central tendency of metabolite abundance, mitigating the impact of sample-specific technical variations. This approach fosters a more robust and comparable analysis of the top features across the data set. The assumption is that normalizing based on the median, known for its lower sensitivity to extreme values, enhances the comparability of top features and ensures a consistent average abundance across samples.

```
#' Compute median and generate normalization factor
mdns <- apply(assay(res, "raw_filled"), MARGIN = 2,
              median, na.rm = TRUE )
nf_mdn <- mdns / median(mdns)

#' divide dataset by median of median and create a new assay.
assays(res)$norm <- sweep(assay(res, "raw_filled"), MARGIN = 2, nf_mdn, '/')
assays(res)$norm_imputed <- sweep(assay(res, "raw_filled_imputed"),
                                  MARGIN = 2, nf_mdn, '/')
```

The median scaling is calculated for both imputed and non-imputed data, with each set stored separately within the `SummarizedExperiment` object. This approach facilitates testing various normalization strategies while maintaining a record of all processing steps undertaken, enabling easy regression to previous stages if necessary.

Assessing overall effectiveness of the normalization approach

It is crucial to evaluate the effectiveness of the normalization process. This can be achieved by comparing the distribution of the log2 transformed feature abundances before and after

normalization. Additionally, the RLA plots can be used to assess the tightness of replicates within groups and compare the behavior between groups.

Principal Component Analysis

```
#' Data before normalization
vals_st <- cbind(vals, phenotype = res$phenotype)
pca_raw <- autoplot(pca_res, data = vals_st,
                    colour = 'phenotype', scale = 0) +
    scale_color_manual(values = col_phenotype)

#' Data after normalization
vals_norm <- apply(assay(res, "norm"), MARGIN = 1, na_unidis) |>
    log2() |>
    scale(center = TRUE, scale = TRUE)

pca_res_norm <- prcomp(vals_norm, scale = FALSE, center = FALSE)
vals_st_norm <- cbind(vals_norm, phenotype = res$phenotype)
pca_adj <- autoplot(pca_res_norm, data = vals_st_norm,
                    colour = 'phenotype', scale = 0) +
    scale_color_manual(values = col_phenotype)

grid.arrange(pca_raw, pca_adj, ncol = 1)
```

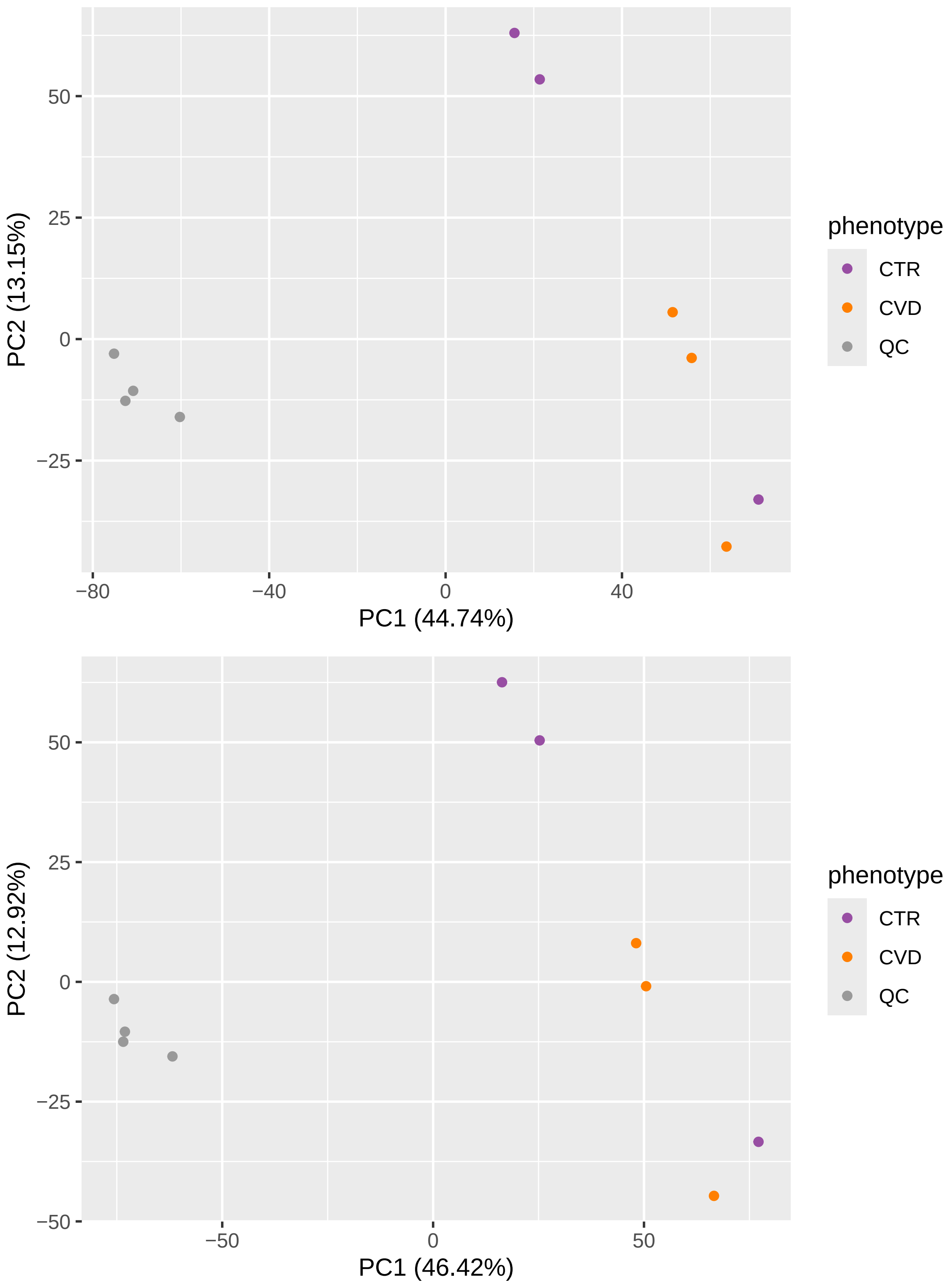

Figure 27: PC1 and PC2 of the data before and after normalization.

Normalization did not have a large impact on PC1 or PC2, but the separation of the study groups on PC3 seems to be better and difference between QC samples lower after normalization (see below).

```
pca_raw <- autoplot(pca_res, data = vals_st ,
                    colour = 'phenotype', x = 3, y = 4, scale = 0) +
    scale_color_manual(values = col_phenotype)
pca_adj <- autoplot(pca_res_norm, data = vals_st_norm,
                    colour = 'phenotype', x = 3, y = 4, scale = 0) +
    scale_color_manual(values = col_phenotype)

grid.arrange(pca_raw, pca_adj, ncol = 1)
```

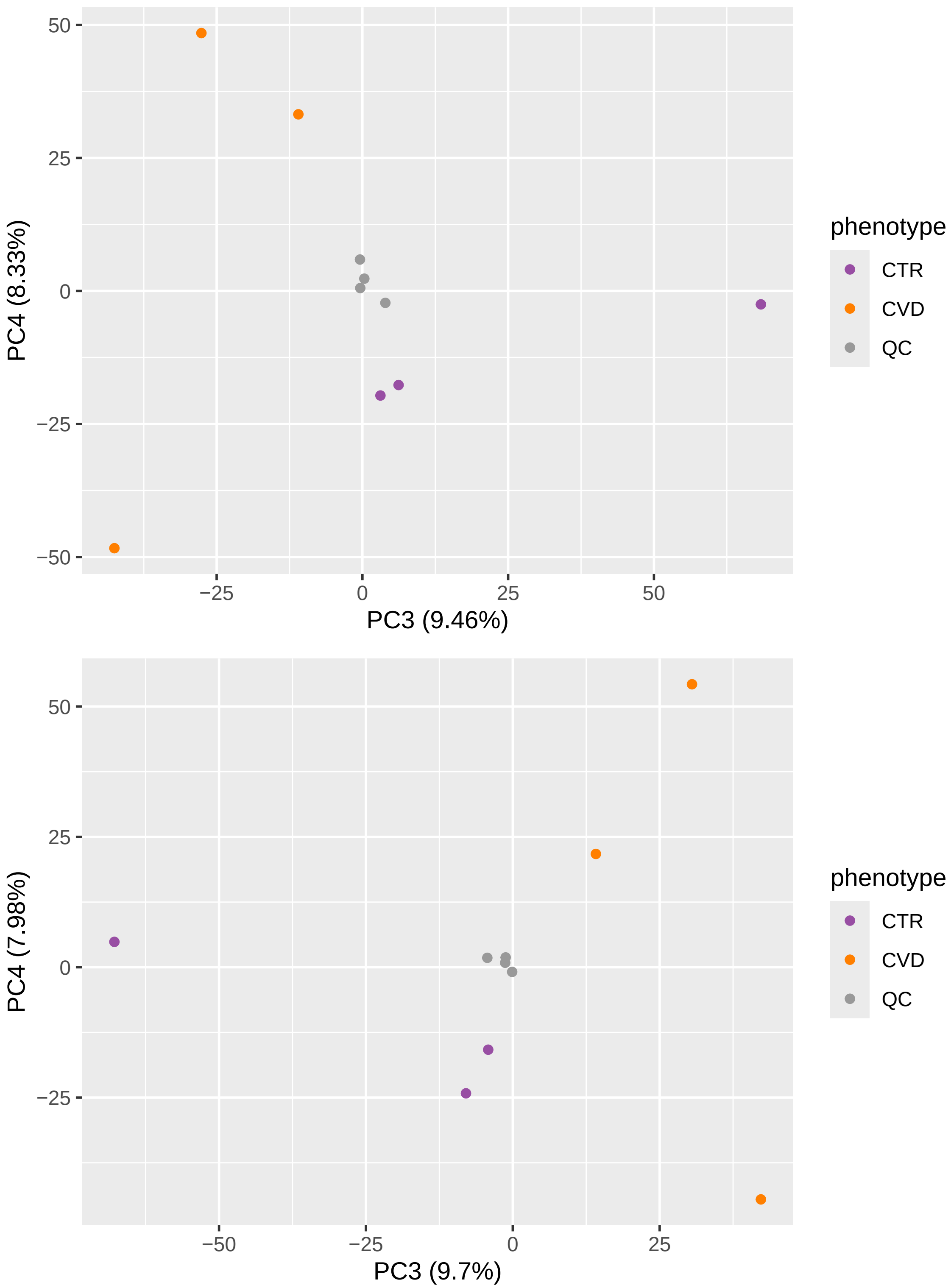

Figure 28: PC3 and PC4 of the data before and after normalization.

The PCA plots above show that the normalization process has not changed the overall structure of the data. The separation between the study and QC samples remains the same. This is an expected results as normalization should not correct for biological variance and only technical.

Intensity evaluation

We compare the RLA plots before and after between-sample normalization to evaluate its impact on the data.

```
par(mfrow = c(2, 1), mar = c(3.5, 4.5, 2.5, 1))

boxplot(rowRla(assay(res, "raw_filled"), group = res$phenotype),
        cex = 0.5, pch = 16, ylab = "RLA", border = col_sample,
        col = paste0(col_sample, 80), cex.main = 1, outline = FALSE,
        xaxt = "n", main = "Raw data", boxwex = 1)
grid(nx = NA, ny = NULL)
legend("topright", inset = c(0, -0.2), col = col_phenotype,
       legend = names(col_phenotype), lty=1, lwd = 2, xpd = TRUE,
       ncol = 3, cex = 0.7, bty = "n")
abline(h = 0, lty=3, lwd = 1, col = "black")

boxplot(rowRla(assay(res, "norm"), group = res$phenotype),
        cex = 0.5, pch = 16, ylab = "RLA", border = col_sample,
        col = paste0(col_sample, 80), boxwex = 1, outline = FALSE,
        xaxt = "n", main = "Normallized data", cex.main = 1)
axis(side = 1, at = seq_len(ncol(res)), labels = res$sample_name)
grid(nx = NA, ny = NULL)
abline(h = 0, lty = 3, lwd = 1, col = "black")
```

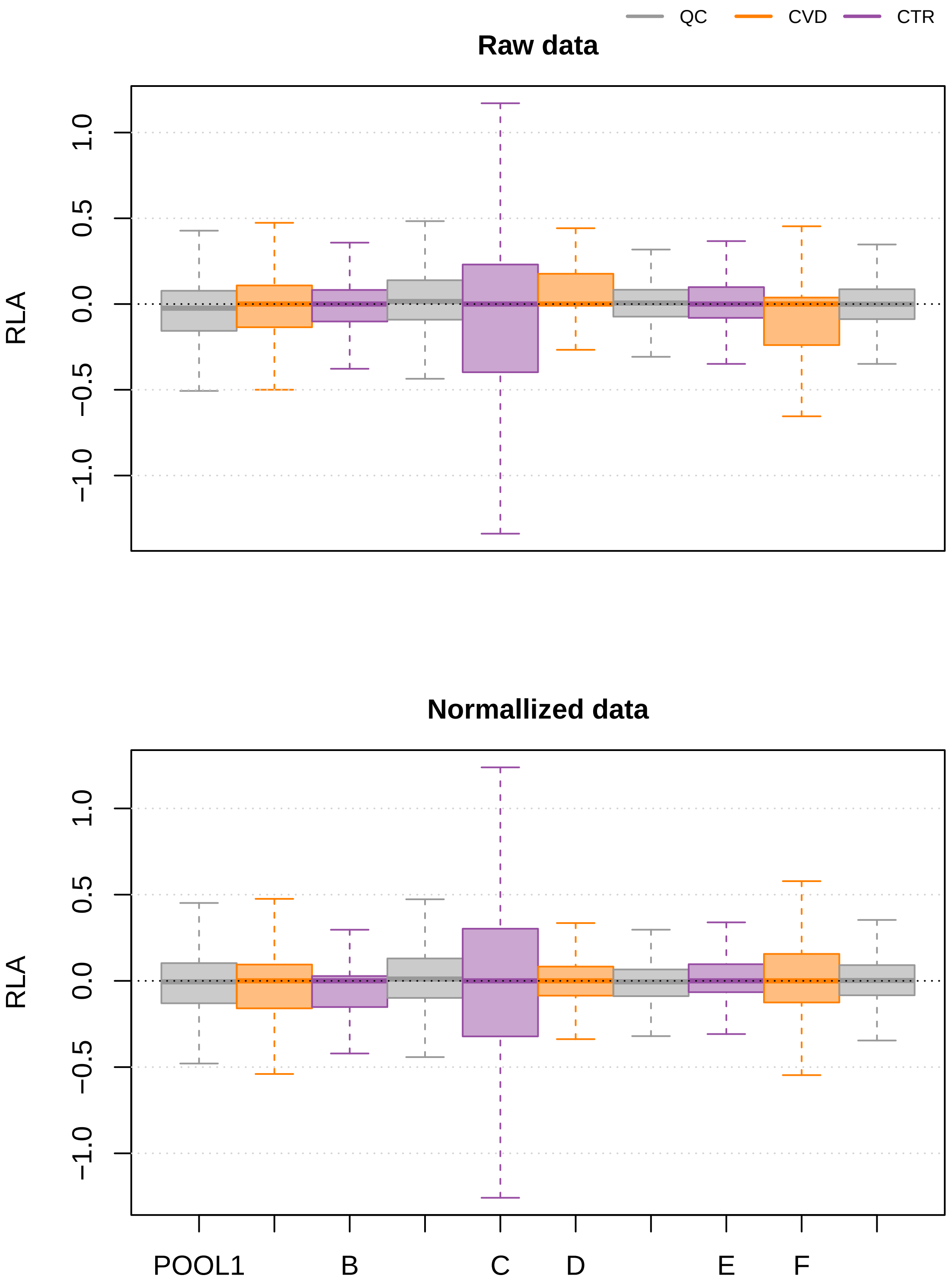

Figure 29: RLA plot before and after normalization.

The normalization process has effectively centered the data around the median and medians for all samples are now closer to zero.

Coefficient of variation

We next evaluate the coefficient of variation (CV, also referred to as *relative standard deviation* RSD) for features across samples from either QC or study samples. In QC samples, the CV would represent technical noise, while in study samples it would include also expected biological differences. Thus, normalization should reduce the CV in QC samples, while only slightly reducing the CV in study samples. The CV is calculated below using the `rowRsd()` function from the *MetaboCoreUtils* package. By setting `mad = TRUE` we use a more robust calculation using the median absolute deviation instead of the standard deviation.

```
#' Calculate the CV values
index_study <- res$phenotype %in% c("CTR", "CVD")
index_QC <- res$phenotype == "QC"

sample_res <- cbind(
    QC_raw = rowRsd(assay(res, "raw_filled")[, index_QC],
                    na.rm = TRUE, mad = TRUE),
    QC_norm = rowRsd(assay(res, "norm")[, index_QC],
                     na.rm = TRUE, mad = TRUE),
    Study_raw = rowRsd(assay(res, "raw_filled")[, index_study],
                       na.rm = TRUE, mad = TRUE),
    Study_norm = rowRsd(assay(res, "norm")[, index_study],
                        na.rm = TRUE, mad = TRUE)
)

#' Summarize the values across features
res_df <- data.frame(
    QC_raw = quantile(sample_res[, "QC_raw"], na.rm = TRUE),
    QC_norm = quantile(sample_res[, "QC_norm"], na.rm = TRUE),
    Study_raw = quantile(sample_res[, "Study_raw"], na.rm = TRUE),
    Study_norm = quantile(sample_res[, "Study_norm"], na.rm = TRUE)
)

pandoc.table(res_df, style = "rmarkdown")
```

	QC_raw	QC_norm	Study_raw	Study_norm
**0%**	0	0	0.0008024	0.002748
**25%**	0.04507	0.04463	0.1424	0.1431

	QC_raw	QC_norm	Study_raw	Study_norm
**50%**	0.0972	0.09708	0.2669	0.2658
**75%**	0.2004	0.1986	0.4867	0.4826
**100%**	1.464	1.464	1.483	1.483

Table 8: Distribution of CV values across samples for the raw and normalized data.

The table above shows the distribution of the CV for both raw and normalized data. The first column highlights the % of the data which is below a given CV value, e.g. 25% of the data has a CV equal or lower than 0.04557 at the *QC_raw* data. As anticipated, the CV values for the QCs, which reflect technical variance, are lower compared to those for the study samples, which include both technical and biological variance. Overall, minimal disparity exists between the raw and normalized data, which is a positive indication that the normalization process has not introduced bias into the dataset, but also reflects the little differences in average abundances between sample in the raw data.

Conclusion on normalization

The overall conclusion of the normalization process is that very little variance was present from the beginning, normalization was however able to center the data around the median (as shown by the RLA plot). Given the simplicity and limited size of our example dataset, we will conclude the normalization process at this stage. For more intricate datasets with diverse biases, a tailored approach would be devised. This could include also approaches to adjust for signal drifts or batch effects. One possible option would be to use a linear-model based approach such as can for example be applied with the `adjust_lm()` function from the *MetaboCoreUtils* package.

Quality control: Feature prefiltering

After normalizing our data we can now pre-filter to clean the data before performing any statistical analysis. In general, pre-filtering of samples and features is performed to remove outliers.

Below we copy the original result object to also keep the unfiltered data for later comparisons.

```
#' Number of features before filtering
nrow(res)
```

```
[1] 8724
```

```r
#' keep unfiltered object
res_unfilt <- res
```

Here we will eliminate features that exhibit high variability in our dataset. Repeatedly measured QC samples typically serve as a robust basis for cleansing datasets allowing to identify features with excessively high noise. As in our data set external QC samples were used, i.e. pooled samples from a different collection and using a slightly different sample matrix, their utility for filtering is somewhat limited. For a comprehensive description and guidelines for data filtering in untargeted metabolomic studies, please refer to (Broadhurst et al. 2018).

Following the guidelines stated above we decided to still use the QC samples for pre-filtering, on the basis that they represent similar bio-fluids to our study samples, and thus, we anticipate observing relatively similar metabolites affected by similar measurement biases.

We therefore evaluate the *dispersion ratio* (Dratio) (Broadhurst et al. 2018) for all features in the data set. We accomplish this task using the same function as above but this time with the `DratioFilter` parameter. More filters exist for this function and we invite the user to explore them as to decide what is best for their dataset.

```r
#' Compute and filter based on the Dratio
filter_dratio <- DratioFilter(threshold = 0.4,
                              qcIndex = res$phenotype == "QC",
                              studyIndex = res$phenotype != "QC",
                              mad = TRUE)
res <- filterFeatures(res, filter = filter_dratio, assay = "norm_imputed")
```

```
4207 features were removed
```

The Dratio filter is a powerful tool to identify features that exhibit high variability in the data, relating the variance observed in QC samples with that in study samples. By setting a threshold of 0.4, we remove features that have a high degree of variability between the QC and study samples. In this example, any feature in which the deviation at the QC is higher than 40% (`threshold = 0.4`)of the deviation on the study samples is removed. This filtering step ensures that only features are retained that have considerably lower technical than biological variance.

Note that the `rowDratio()` and `rowRsd()` functions from the *MetaboCoreUtils* package could be used to calculate the actual numeric values for the estimates used for filtering, to e.g. to evaluate their distribution in the whole data set or identify data set-dependent threshold values.

Finally, we evaluate the number of features left after the filtering steps and calculate the percentage of features that were removed.

```r
#' Number of features after analysis
nrow(res)
```

```
[1] 4517
```

```r
#' Percentage left: end/beginning
nrow(res)/nrow(res_unfilt) * 100
```

```
[1] 51.77671
```

The dataset has been reduced from 8724 to 4517 features. We did remove a considerable amount of features but this is expected as we want to focus on the most reliable features for our analysis. For the rest of our analysis we need to separate the QC samples from the study samples. We will store the QC samples in a separate object for later use.

```r
res_qc <- res[, res$phenotype == "QC"]
res <- res[, res$phenotype != "QC"]
```

We in addition calculate the CV of the QC samples and add that as an additional column to the `rowData()` of our result object. These could be used later to prioritize identified *significant* features with e.g. low technical noise.

```r
#' Calculate the QC's CV and add as feature variable to the data set
rowData(res)$qc_cv <- assay(res_qc, "norm") |>
               rowRsd()
```

Now that our data set has been preprocessed, normalized and filtered, we can start to evaluate the distribution of the data and estimate the variation due to biology.

Overlapping features

Before performing complex statistical analysis we do some short quality analysis. A possible evaluation is to which features are overlapping between the different groups. In our case grouping per condition (CTR versus CVD group) would be the most interesting plot. However we want to address the case when people test more than 3 condition therefore we will show the overlapping feature per sample (therefore comparing 6 groups).

```r
df_logical <- !is.na(assay(res, "raw_filled"))

# Create a Venn diagram
library(ggVennDiagram)
l_logical <- apply(df_logical, MARGIN = 2,function(i) which(i))
ggVennDiagram(l_logical, label = "count",
                             category.names = res$sample_name,
              color = 1, lwd = 0.7) +
 scale_fill_gradient(low = "lightblue", high = "#4981BF") +
 theme(legend.position = "none")
```

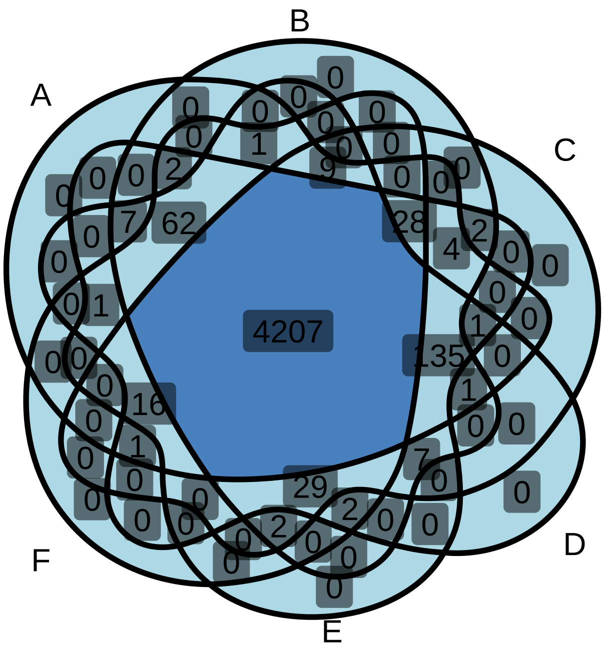

In case of a large number of groups a Venn diagram can be replace by an Upset plot which can handle a larger number of groups, while being still readable.

```r
#make upsetplot
library(UpSetR)
binary_data <-apply(df_logical, MARGIN = 2, as.integer)
upset(as.data.frame(binary_data), nset = 6,
      sets = colnames(df_logical), keep.order = TRUE)
```

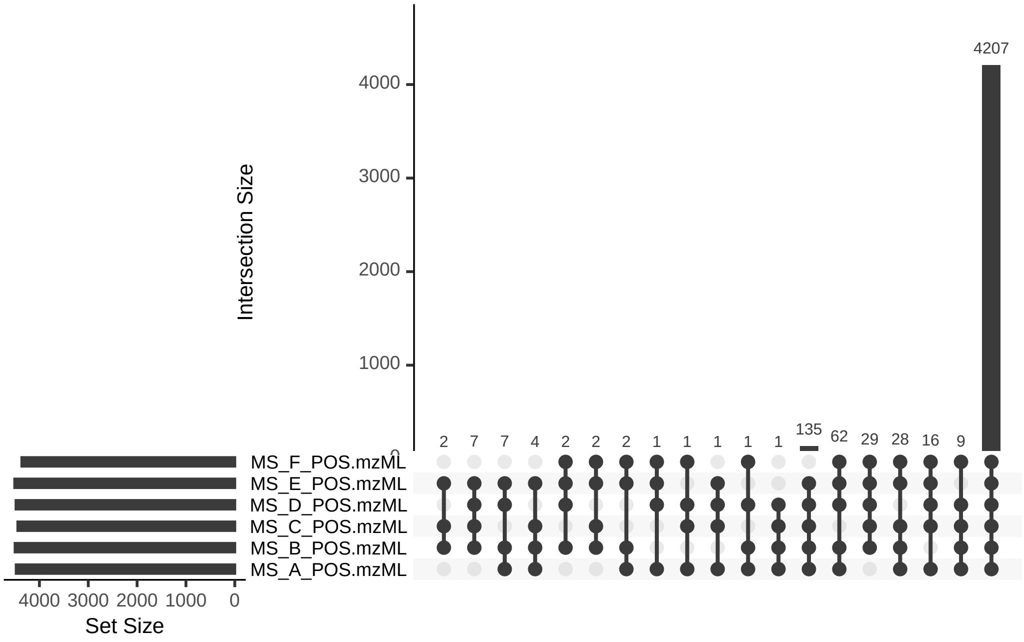

Other quality analysis of the data could be, evaluating the number of feature detected per group, the overall abundance, the noise, … This all depends on the goal of the user research. R has a lot of flexibility, you can do many things !

Differential abundance analysis

After normalization and quality control, the next step is to identify features that are differential abundant between the study groups. This crucial step allows us to identify potential biomarkers or metabolites that are associated with the study groups. There are various approaches and methods available for the identification of such features of interest. In this workflow we use a multiple linear regression analysis to identify features with significantly difference in abundances between the CVD or CTR study group.

Before performing the tests we evaluate similarities between study samples using a PCA (after excluding the QC samples to avoid them influencing the results).

```
#' Define the colors for the plot
col_sample <- col_phenotype[res$phenotype]

#' Log transform and scale the data for PCA analysis
vals <- assay(res, "norm_imputed") |>
    t() |>
```

```
    log2() |>
    scale(center = TRUE, scale = TRUE)
pca_res <- prcomp(vals, scale = FALSE, center = FALSE)

vals_st <- cbind(vals, phenotype = res$phenotype)
autoplot(pca_res, data = vals_st , colour = 'phenotype', scale = 0) +
    scale_color_manual(values = col_phenotype)
```

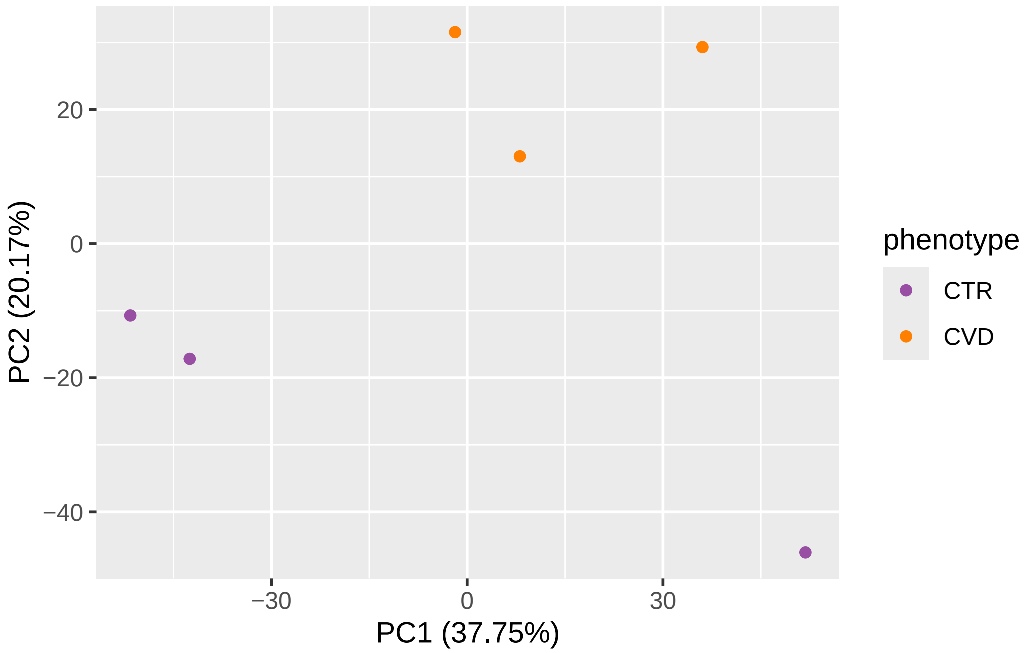

Figure 30: PCA of the data after normalization and quality control.

The samples clearly separate by study group on PCA indicating that there are differences in the metabolite profiles between the two groups. However, what drives the separation on PC1 is not clear. Below we evaluate whether this could be explained by the other available variable of our study, i.e., age:

```
#' Add age to the PCA plot
vals_st <- cbind(vals, age = res$age)
autoplot(pca_res, data = vals_st , colour = 'age', scale = 0)
```

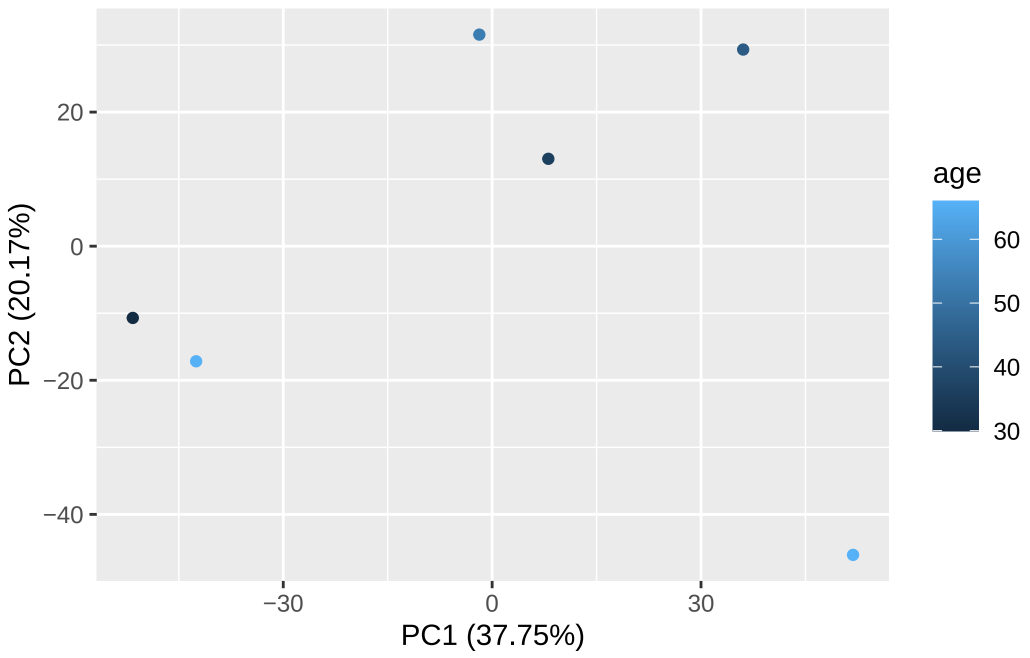

Figure 31: PCA colored by age of the data after normalization and quality control.

According to the PCA above, PC1 does not seem to be related to age.

Even if there is some variance in the data set we can't explain at this stage, we proceed with the (supervised) statistical tests to identify features of interest. We compute linear models for each metabolite explaining the observed feature abundance by the available study variables. While we could also use the base R function `lm()`, we utilize the R `Biocpkg("limma")` package to conduct the differential abundance analysis: the *moderated* test statistics (Smyth 2004) provided by this package are specifically well suited for experiments with a limited number of replicates. For our tests we use a linear model `~ phenotype + age`, hence explaining the abundances of one metabolite accounting for the study group assignment and age of each individual. The analysis might benefit from inclusion of a study covariate associated with PC2 or explaining the variance seen in that principal component, but for the present analysis only the participant's age and disease association was provided.

Below we define the design of the study with the `model.matrix()` function and fit feature-wise linear models to the log2-transformed abundances using the `lmFit()` function. P-values for significance of association are then calculated using the `eBayes()` function, that also performs the empirical Bayes-based robust estimation of the standard errors. See also the excellent vignette/user guide of the *limma* package for examples and details on the linear model procedure.

```r
#' Define the linear model to be applied to the data
p.cut <- 0.05     # cut-off for significance.
m.cut <- 0.5      # cut-off for log2 fold change

age <- res$age
phenotype <- factor(res$phenotype)
design <- model.matrix(~ phenotype + age)

#' Fit the linear model to the data, explaining metabolite
#' concentrations by phenotype and age.
fit <- lmFit(log2(assay(res, "norm_imputed")), design = design)
fit <- eBayes(fit)
```

After the linear models have been fitted, we can now proceed to extract the results. We create a data frame containing the coefficients, raw and adjusted p-values (applying a Benjamini-Hochberg correction, i.e., `method = "BH"` for improved control of the false discovery rate), the average intensity of signals in CVD and CTR samples, and an indication of whether a feature is deemed significant or not. We consider all metabolites with an adjusted p-value smaller than 0.05 to be significant, but we could also include the (absolute) difference in abundances in the cut-off criteria. At last, we add the differential abundance results to the result object's `rowData()`.

```r
#' Compile a result data frame
tmp <- data.frame(
    coef.CVD = fit$coefficients[, "phenotypeCVD"],
    pvalue.CVD = fit$p.value[, "phenotypeCVD"],
    adjp.CVD = p.adjust(fit$p.value[, "phenotypeCVD"], method = "BH"),
    avg.CVD = rowMeans(
        log2(assay(res, "norm_imputed")[, res$phenotype == "CVD"])),
    avg.CTR = rowMeans(
        log2(assay(res, "norm_imputed")[, res$phenotype == "CTR"]))
)
tmp$significant.CVD <- tmp$adjp.CVD < 0.05
#' Add the results to the object's rowData
rowData(res) <- cbind(rowData(res), tmp)
```

We can now proceed to visualize the distribution of the raw and adjusted p-values.

```r
#' Plot the distribution of p-values
par(mfrow = c(1, 2))
hist(rowData(res)$pvalue.CVD, breaks = 64, xlab = "p value",
```

```
     main = "Distribution of raw p-values",
     cex.main = 1, cex.lab = 1, cex.axis = 1)
hist(rowData(res)$adjp.CVD, breaks = 64, xlab = expression(p[BH]~value),
     main = "Distribution of adjusted p-values",
     cex.main = 1, cex.lab = 1, cex.axis = 1)
```

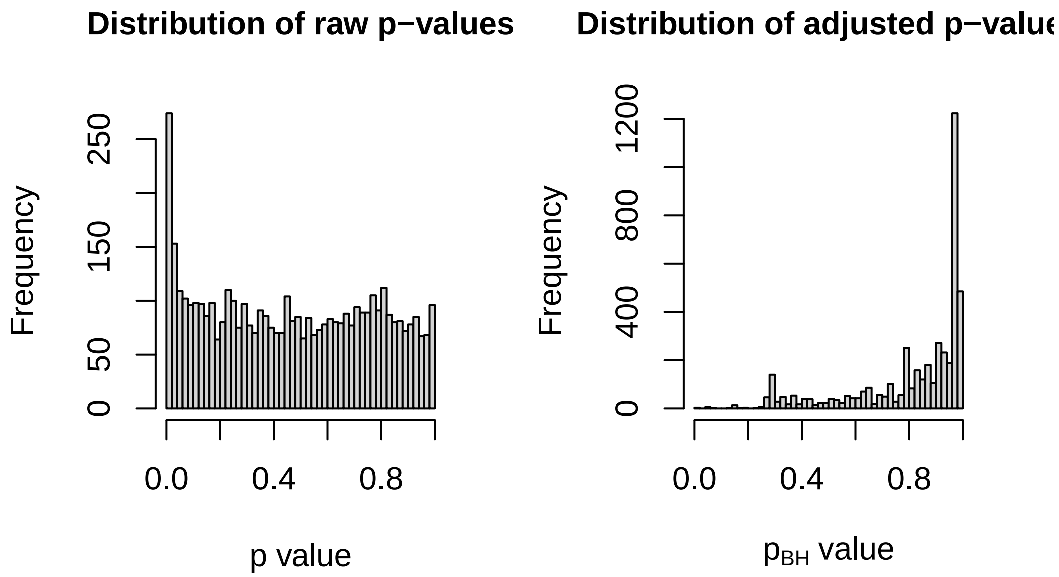

Figure 32: Distribution of raw (left) and adjusted p-values (right).

The histograms above show the distribution of raw and adjusted p-values. Except for an enrichment of small p-values, the raw p-values are (more or less) uniformly distributed, which indicates the absence of any strong systematic biases in the data. The adjusted p-values are more conservative and account for multiple testing; this is important here as we fit a linear model to each feature and therefore perform a large number of tests which leads to a high chance of false positive findings. We do see that some features have very low p-values, indicating that they are likely to be significantly different between the two study groups.

Below we plot the adjusted p-values against the log2 fold change of (average) abundances. This volcano plot will allow us to visualize the features that are significantly different between the two study groups. These are highlighted with a blue color in the plot below.

```
#' Plot volcano plot of the statistical results
par(mfrow = c(1, 1), mar = c(5, 5, 5, 1))
```

```
plot(rowData(res)$coef.CVD, -log10(rowData(res)$adjp.CVD),
     xlab = expression(log[2]~difference),
     ylab = expression(-log[10]~p[BH]), pch = 16, col = "#00000060",
     cex.main = 1.5, cex.lab = 1.5, cex.axis = 1.3)
grid()
abline(h = -log10(0.05), col = "#0000ffcc")
if (any(rowData(res)$significant.CVD)) {
    points(rowData(res)$coef.CVD[rowData(res)$significant.CVD],
           -log10(rowData(res)$adjp.CVD[rowData(res)$significant.CVD]),
           col = "#0000ffcc")
}
```

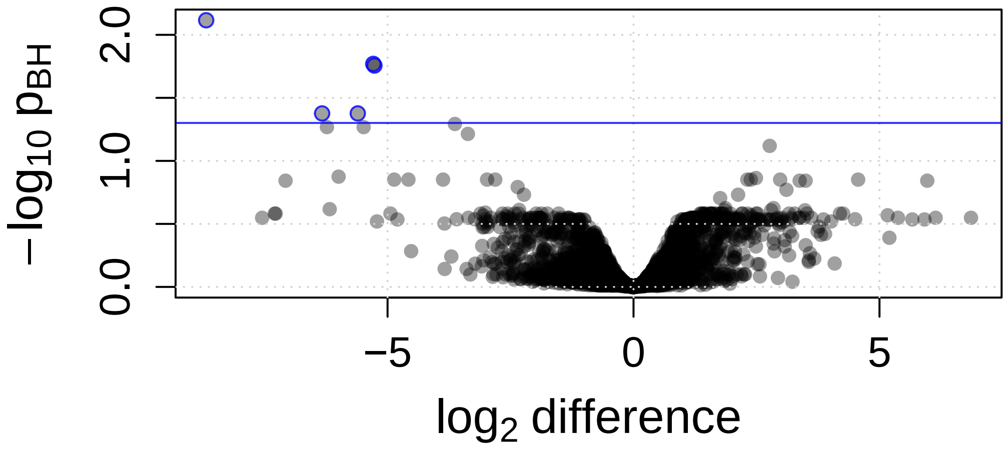

Figure 33: Volcano plot showing the analysis results.

The most interesting features are in the top corners in the volcano plot (i.e., features with a large difference in abundance between the groups and a small p-value). All significant features have a negative coefficient (log2 fold change value) indicating that their abundance is lower in CVD samples compared to the CTR samples. These features are listed, along with their average difference in (log2) abundance between the compared groups, their adjusted p-values, their average (log2) abundance in each sample group and their RSD (CV) in QC samples in the table below.

```r
# Table of significant features
tab <- rowData(res)[rowData(res)$significant.CVD,
                    c("mzmed", "rtmed", "coef.CVD", "adjp.CVD",
                      "avg.CTR", "avg.CVD", "qc_cv")] |>
    as.data.frame()
tab <- tab[order(abs(tab$coef.CVD), decreasing = TRUE), ]
pandoc.table(tab, style = "rmarkdown")
```

	mzmed	rtmed	coef.CVD	adjp.CVD	avg.CTR	avg.CVD
**FT0732**	182.1	34.84	-8.687	0.007661	12.23	3.668
**FT0845**	195.1	32.66	-6.33	0.04203	16.91	10.45
**FT0565**	161	162.1	-5.605	0.04203	10.29	4.482
**FT1171**	229.1	181.1	-5.29	0.017	10.72	5.597
**FT0371**	138.1	148.4	-5.267	0.01753	9.915	4.311

Table 9: Table continues below

	qc_cv
**FT0732**	0.2116
**FT0845**	0.03047
**FT0565**	0.03609
**FT1171**	0.07063
**FT0371**	0.5564

Features with significant differences in abundances.

Below we visualize the EICs of the significant features to evaluate their (raw) signal. We restrict the MS data set to study samples. Parameters

- `keepFeatures = TRUE`: ensures that identified features are retained in the subset object.
- `peakBg`: defines the (background) color for each individual chromatographic peak in the EIC object.

```r
#' Restrict the raw data to study samples.
lcms1_study <- lcms1[sampleData(lcms1)$phenotype != "QC", keepFeatures = TRUE]
#' Extract EICs for the significant features
eic_sign <- featureChromatograms(
    lcms1_study, features = rownames(tab), expandRt = 5, filled = TRUE)
```

```
#' Plot the EICs.
plot(eic_sign, col = col_sample,
     peakBg = paste0(col_sample[chromPeaks(eic_sign)[, "sample"]], 40))
legend("topright", col = col_phenotype,
       legend = names(col_phenotype), lty = 1)
```

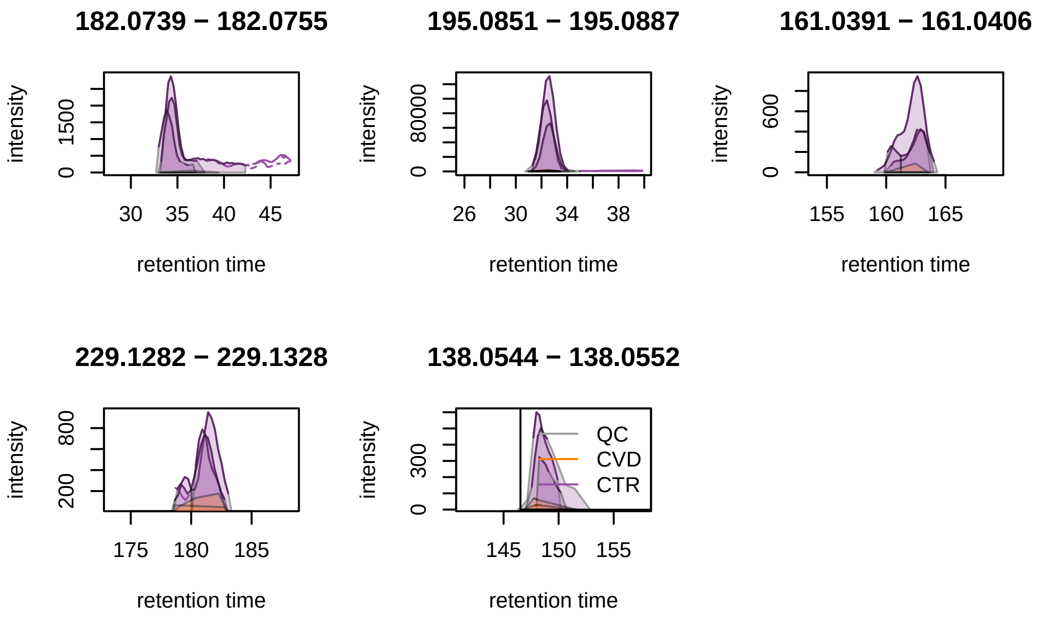

Figure 34: Extracted ion chromatograms of the significant features.

The EICs for all significant features show a clear and single peak. Their intensities are (as already observed above) much larger in the CTR than in the CVD samples. With the exception of the second feature (second EIC in the top row), the intensities for all significant features are however generally low. This might make it challenging to identify them using an LC-MS/MS setup.

Annotation

We have now identified the features with significant differences in abundances between the two study groups. These provide information on metabolic pathways that differentiate affected from healthy individuals and might hence also serve as biomarkers. However, at this stage

of the analysis we do not know what compounds/metabolites they actually represent. We thus need now to annotate these signals. Annotation can be performed at different level of confidence Schymanski et al. (2014). The lowest level of annotation, with the highest rate of false positive hits, bases on the features m/z ratios. Higher levels of annotations employ fragment spectra (MS2 spectra) of ions of interest requiring this however acquisition of additional data. In this section, we will demonstrate multiple ways to annotate our significant features using functionality provided through Bioconductor packages. Alternative approaches and external software tools, which may be better suited, will also be discussed later in the section.

MS1-based annotation

Our data set was acquired using an LC-MS setup and our features are thus only characterized by their m/z and retention times. The retention time is LC-setup-specific and, without prior data/knowledge provide only little information on the features' identity. Modern MS instruments have a high accuracy and the m/z values should therefore be reliable estimates of the compound ion's mass-to-charge ratio. As first approach, we use the features' m/z values and match them against reference values, i.e., exact masses of chemical compounds that are provided by a reference database, in our case the MassBank database. The full MassBank data is re-distributed through Bioconductor's *AnnotationHub* resource, which simplifies their integration into reproducible R-based analysis workflows.

Below we load the resource, list all available MassBank data sets/releases and load one of them.

```r
#' load reference data
ah <- AnnotationHub()
```

```r
#' List available MassBank data sets
query(ah, "MassBank")
```

```
AnnotationHub with 8 records
# snapshotDate(): 2025-10-29
# $dataprovider: MassBank
# $species: NA
# $rdataclass: CompDb
# additional mcols(): taxonomyid, genome, description,
#   coordinate_1_based, maintainer, rdatadateadded, preparerclass, tags,
#   rdatapath, sourceurl, sourcetype
# retrieve records with, e.g., 'object[["AH107048"]]'

             title
```

```
  AH107048 | MassBank CompDb for release 2021.03
  AH107049 | MassBank CompDb for release 2022.06
  ...        ...
  AH119518 | MassBank CompDb for release 2024.06
  AH119519 | MassBank CompDb for release 2024.11
```

```
#' Load one MassBank release
mb <- ah[["AH116166"]]
```

The MassBank data is provided as a self-contained SQLite database and data can be queried and accessed through the *CompoundDb* Bioconductor package. Below we use the `compounds()` function to extract small compound annotations from the database.

```
#' Extract compound annotations
cmps <- compounds(mb, columns = c("name", "formula",
                                  "exactmass", "inchikey"))
head(cmps)
```

```
        formula exactmass                    inchikey                 name
1    C27H29NO11  543.1741 AOJJSUZBOXZQNB-UHFFFAOYSA-N           Epirubicin
2       C40H54O4  598.4022 KFNGKYUGHHQDEE-AXWOCEAUSA-N Crassostreaxanthin A
3    C10H24N2O2  204.1838 AEUTYOVWOVBAKS-UWVGGRQHSA-N           Ethambutol
4     C16H27NO5  313.1889 LMFKRLGHEKVMNT-UJDVCPFMSA-N           Heliotrine
5 C20H15Cl3N2OS  435.9971 JLGKQTAYUIMGRK-UHFFFAOYSA-N        Sertaconazole
6       C15H14O5  274.0841 BWNCKEBBYADFPQ-UHFFFAOYSA-N     (R)Semivioxanthin
```

MassBank (as most other small compound annotation databases) provides the (exact) molecular mass of each compound. Since almost all small compounds are neutral in their natural state, they need to be first converted to m/z values to allow matching against the feature's m/z. To calculate an m/z from a neutral mass, we need to assume which ion (adduct) might be generated from the measured metabolites by the employed electro-spray ionization. In positive polarity, for human serum samples, the most common ions will be protonated (*[M+H]+*), or bear the addition of sodium (*[M+Na]+*) or removal of ammonium (*[M+H-NH3]+*) ions. To match the observed m/z values against reference values from these potential ions we use again the `matchValues()` function with the `Mass2MzParam` approach, that allows to specify the types of expected ions with its `adducts` parameter and the maximal allowed difference between the compared values using the `tolerance` and `ppm` parameters. Below we first prepare the data.frame with the significant features, set up the parameters for the matching and perform the comparison of the query features against the reference database.

```r
#' Prepare query data frame
rowData(res)$feature_id <- rownames(rowData(res))
res_sig <- res[rowData(res)$significant.CVD, ]

#' Setup parameters for the matching
param <- Mass2MzParam(adducts = c("[M+H]+", "[M+Na]+", "[M+H-NH3]+"),
                      tolerance = 0, ppm = 5)

#' Perform the matching.
mtch <- matchValues(res_sig, cmps, param = param, mzColname = "mzmed")
mtch
```

```
Object of class Matched
Total number of matches: 43
Number of query objects: 5 (4 matched)
Number of target objects: 25685 (43 matched)
```

The resulting `Matched` object shows that 4 of our 6 significant features could be matched to ions of compounds from the MassBank database. Below we extract the full result from the `Matched` object.

```r
#' Extracting the results
mtch_res <- matchedData(mtch, c("feature_id", "mzmed", "rtmed",
                                "adduct", "ppm_error",
                                "target_formula", "target_name",
                                "target_inchikey"))
mtch_res
```

```
DataFrame with 44 rows and 8 columns
        feature_id     mzmed     rtmed      adduct ppm_error target_formula
       <character> <numeric> <numeric> <character> <numeric>    <character>
FT0371      FT0371   138.055   148.396      [M+H]+   2.08055        C7H7NO2
FT0371      FT0371   138.055   148.396      [M+H]+   1.93568        C7H7NO2
...            ...       ...       ...         ...       ...            ...
FT0845      FT0845   195.088   32.6567      [M+H]+  0.163988      C8H10N4O2
FT1171      FT1171   229.130  181.0883     [M+Na]+  3.077084      C12H18N2O
         target_name target_inchikey
         <character>     <character>
FT0371 Benzohydro...    VDEUYMSGMP...
FT0371 Trigonelli...    WWNNZCOKKK...
...              ...              ...
```

```
FT0845 1,3,7-TRIM...   RYYVLZVUVI...
FT1171 Isoproturo...   PUIYMUZLKQ...
```

Thus, in total 43 ions of compounds in MassBank were matched to our significant features based on the specified tolerance settings. Many compounds, while having a different structure and thus function/chemical property, have an identical chemical formula and thus mass. Matching exclusively on the *m/z* of features will hence result in many potentially false positive hits and is thus considered to provide only low confidence annotation. An additional complication is that some annotation resources, like MassBank, being community maintained, contain a large amount of redundant information. To reduce redundancy in the result table we below iterate over the hits of a feature and keep only matches to unique compounds (identified by their INCHIKEY). The INCHI or INCHIKEY combine information from compound's chemical formula and structure, so while different compounds can share the same chemical formula, they should have a different structure and thus INCHI.

```r
rownames(mtch_res) <- NULL

#' Keep only info on features that machted - create a utility function for that
mtch_res <- split(mtch_res, mtch_res$feature_id) |>
    lapply(function(x) {
        lapply(split(x, x$target_inchikey), function(z) {
            z[which.min(z$ppm_error), ]
        })
    }) |>
    unlist(recursive = FALSE) |>
    do.call(what = rbind)

#' Display the results
pandoc.table(as.data.frame(mtch_res), style = "rmarkdown")
```

feature_id	mzmed	rtmed	adduct	ppm_error	target_formula
FT0371	138.1	148.4	[M+H]+	1.936	C7H7NO2
FT0371	138.1	148.4	[M+H]+	1.936	C7H7NO2
FT0371	138.1	148.4	[M+H]+	1.936	C7H7NO2
FT0371	138.1	148.4	[M+H]+	1.936	C7H7NO2
FT0371	138.1	148.4	[M+H]+	1.936	C7H7NO2
FT0371	138.1	148.4	[M+H]+	1.936	C7H7NO2
FT0371	138.1	148.4	[M+H]+	1.925	C7H7NO2
FT0371	138.1	148.4	[M+H]+	1.936	C7H7NO2
FT0371	138.1	148.4	[M+H]+	1.936	C7H7NO2
FT0371	138.1	148.4	[M+H]+	1.936	C7H7NO2

feature_id	mzmed	rtmed	adduct	ppm_error	target_formula
FT0371	138.1	148.4	[M+H]+	1.925	C7H7NO2
FT0371	138.1	148.4	[M+H]+	1.936	C7H7NO2
FT0371	138.1	148.4	[M+H]+	1.936	C7H7NO2
FT0371	138.1	148.4	[M+H]+	1.936	C7H7NO2
FT0371	138.1	148.4	[M+H]+	1.936	C7H7NO2
FT0371	138.1	148.4	[M+H]+	1.936	C7H7NO2
FT0565	161	162.1	[M+Na]+	3.125	C8H10S
FT0845	195.1	32.66	[M+H]+	0.06147	C8H10N4O2
FT0845	195.1	32.66	[M+H]+	0.06147	C8H10N4O2
FT0845	195.1	32.66	[M+H]+	0.1866	C8H10N4O2
FT1171	229.1	181.1	[M+Na]+	3.077	C12H18N2O

Table 11: Table continues below

target_name	target_inchikey
4-Aminobenzoic acid	ALYNCZNDIQEVRV-UHFFFAOYSA-N
2-PYRIDINECARBOXYLIC ACID METHYL ESTER	NMMIHXMBOZYNET-UHFFFAOYSA-N
4-PYRIDINECARBOXYLIC ACID METHYL ESTER	OLXYLDUSSBULGU-UHFFFAOYSA-N
SALICYLALDOXIME	ORIHZIZPTZTNCU-VMPITWQZSA-N
ORTHO NITROTOLUENE	PLAZTCDQAHEYBI-UHFFFAOYSA-N
4-PYRIDYL ACETATE	PTZQTYADHHANGW-UHFFFAOYSA-N
Salicylaldehyde oxime	PUNVNOQWQQPNES-UHFFFAOYSA-N
META NITROTOLUENE	QZYHIOPPLUPUJF-UHFFFAOYSA-N
Anthranilic acid	RWZYAGGXGHYGMB-UHFFFAOYSA-N
2-HYDROXYBENZAMIDE	SKZKKFZAGNVIMN-UHFFFAOYSA-N
N-Hydroxybenzamide	VDEUYMSGMPQMIK-UHFFFAOYSA-N
3-Pyridylacetic acid	WGNUNYPERJMVRM-UHFFFAOYSA-N

target_name	target_inchikey
Trigonelline	WWNNZCOKKKDOPX-UHFFFAOYSA-N
META-AMINOBENZOIC ACID	XFDUHJPVQKIXHO-UHFFFAOYSA-N
3-PYRIDINECARBOXYLIC ACID METHYL ESTER	YNBADRVTZLEFNH-UHFFFAOYSA-N
4-NITROTOLUENE	ZPTVNYMJQHSSEA-UHFFFAOYSA-N
BENZYL METHYL SULFIDE	OFQPKKGMNWASPN-UHFFFAOYSA-N
Isocaffeine	LPHGQDQBBGAPDZ-UHFFFAOYSA-N
Caffeine	RYYVLZVUVIJVGH-UHFFFAOYSA-N
1,3-Benzenedicarboxylic acid, dihydrazide	UTTHLMXOSUFZCQ-UHFFFAOYSA-N
Isoproturon	PUIYMUZLKQOUOZ-UHFFFAOYSA-N

MS1 annotation results.

The table above shows the results of the MS1-based annotation process. We can see that four of our significant features were matched. The matches seem to be pretty accurate with low ppm errors. The deduplication performed above considerably reduced the number of hits for each feature, but the first still matches ions of a large number of compounds (all with the same chemical formula).

Considering both features' *m/z* and retention times in the MS1-based annotation will increase the annotation confidence, but requires additional data, such as recording of the retention time of thepure standard for a compound on the same LC setup. An alternative approach which might provide better inside on annotations and help to choose between different annotations for a same feature is to evaluate certain chemical properties of the possible matches. For instance, the LogP value, available in several databases such HMDB, provides an insight on a given compound's polarity. As this property highly affects the interaction of the analyte with the column, it usually also directly affects separation. Therefore, a comparison between an analyte's retention time and polarity can help to rule out some possible misidentifications.

While being of low confidence, MS1-based annotation can provide first candidate annotations that could be confirmed or rejected by additional analyses.

MS2-based annotation

MS1 annotation is a fast and efficient method to annotate features and therefore give a first insight into the compounds that are significantly different between the two study groups. However, it is not always the most accurate. MS2 data can provide a higher level of confidence in the annotation process as it provides, through the observed fragmentation pattern, information on the structure of the compound.

MS2 data can be generated through LC-MS/MS measurement in which MS2 spectra are recorded for ions either in a data dependent acquisition (DDA) or data independent acquisition (DIA) mode. Generally, it is advised to include some LC-MS/MS runs of QC samples or randomly selected study samples already during the acquisition of the MS1 data that is used for quantification of the signals. As an alternative, or in addition, a post-hoc LC-MS/MS acquisition can be performed to generate the MS2 data needed for annotation. For the present experiment, a separate LC-MS/MS measurement was conducted on QC samples and selected study samples to generate such data using an *inclusion list* of pre-selected ions. These represent features found to be significantly different between CVD and CTR samples in an initial analysis of the full experiment. We use a subset of this second LC-MS/MS data set to show how such data can be used for MS2-based annotation. In our differential abundance analysis we found features with significantly higher abundances in CTR samples. Consequently, we will utilize the MS2 data obtained from the CTR samples to annotate these significant features.

Below we load the LC-MS/MS data from the experiment and restrict it to data acquired from the CTR sample.

```
#' Load form the MetaboLights Database
param <- MetaboLightsParam(mtblsId = "MTBLS8735",
                           assayName = paste0("a_MTBLS8735_LC-MSMS_positive_",
                           "hilic_metabolite_profiling.txt"),
                           filePattern = ".mzML")

lcms2 <- readMsObject(MsExperiment(),
                      param,
                      keepOntology = FALSE,
                      keepProtocol = FALSE,
                      simplify = TRUE)
```

```
#adjust sampleData
colnames(sampleData(lcms2)) <-
    c("sample_name", "derived_spectra_data_file",
      "metabolite_asssignment_file", "source_name",
      "organism", "blood_sample_type",
      "sample_type", "age", "unit", "phenotype")
```

```
# Identify MS/MS data files for control samples
keep <- sampleData(lcms2)$phenotype == "CTR" &
        grepl("MSMS", sampleData(lcms2)$derived_spectra_data_file)

# filter samples to keep MSMS data from CTR samples:
lcms2 <- lcms2[keep]

# Add fragmentation data information (from filenames)
sampleData(lcms2)$fragmentation_mode <- c("CE20", "CE30", "CES")

#let's look at the updated sample data
sampleData(lcms2)[, c("derived_spectra_data_file",
                      "phenotype", "sample_name", "age")] |>
    as.data.frame() |>
    pandoc.table(style = "rmarkdown")
```

derived_spectra_data_file	phenotype	sample_name	age
FILES/MSMS_2_E_CE20_POS.mzML	CTR	E	66
FILES/MSMS_2_E_CE30_POS.mzML	CTR	E	66
FILES/MSMS_2_E_CES_POS.mzML	CTR	E	66

Table 13: Samples from the LC-MS/MS data set.

```
#' Filter the data to the same RT range as the LC-MS run
lcms2 <- filterSpectra(lcms2, filterRt, c(10, 240))
```

In total we have 3 LC-MS/MS data files for the control samples, each with a different collision energy to fragment the ions. Below we show the number of MS1 and MS2 spectra for each of the files (each column being one sample/file, with the first row providing the number of MS1, the second of MS2 spectra per file).

```
#' check the number of spectra per ms level
spectra(lcms2) |>
    msLevel() |>
    split(spectraSampleIndex(lcms2)) |>
    lapply(table) |>
    do.call(what = cbind)
```

```
      1    2    3
```

```
1  186  186  186
2 3123 3118 3120
```

Compared to the number of MS2 spectra, far less MS1 spectra were acquired. The configuration of the MS instrument was set to ensure that the ions specified in the *inclusion list* were selected for fragmentation, even if their intensity might be very low. With this setting, however, most of the recorded MS2 spectra will represent only noise. The plot below shows the location of precursor ions in the m/z - retention time plane for the three files.

```
plotPrecursorIons(lcms2)
```

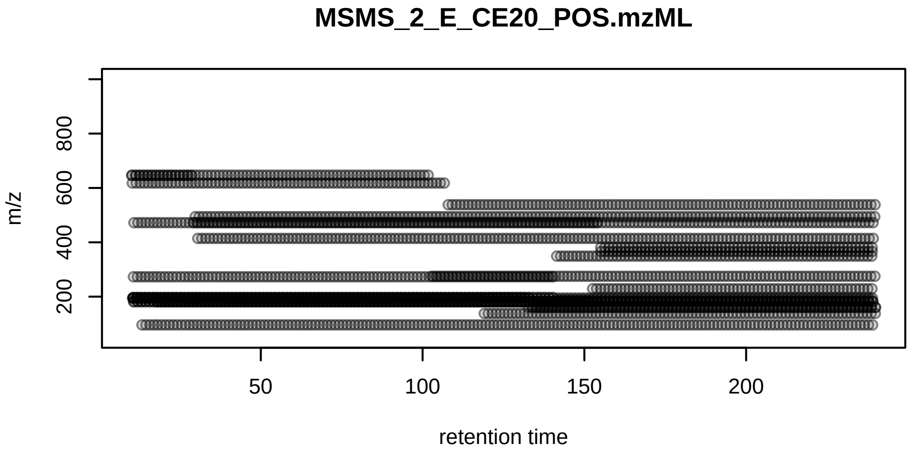

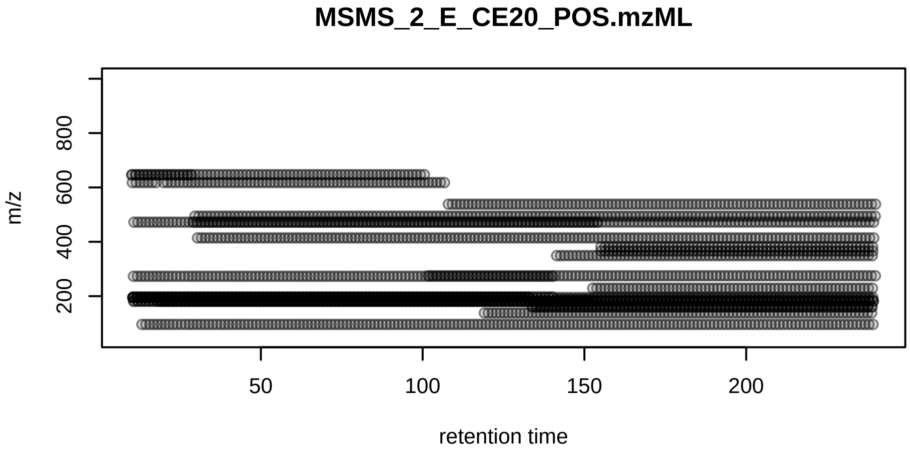

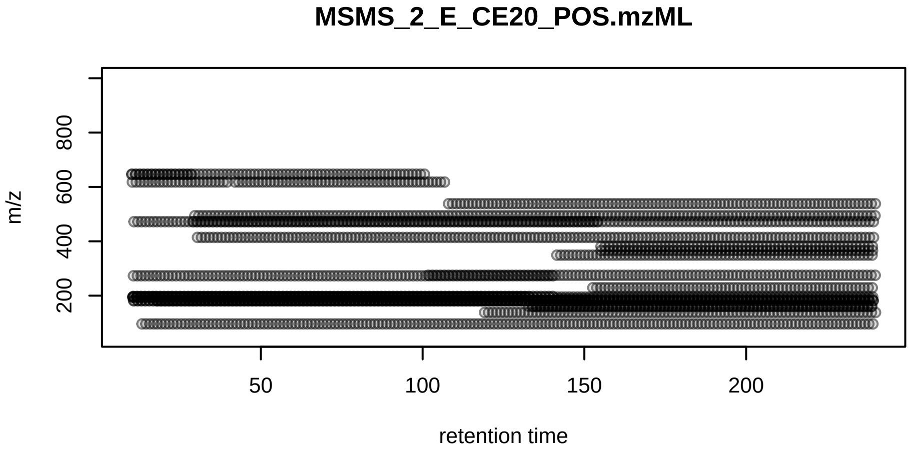

Figure 35: Precursor ions in the LC-MS/MS data set.

We can see MS2 spectra being recorded for the m/z of interest along the full retention time range, even if the actual ions will only be eluting within certain retention time windows. For the present data set the instrument configuration was thus not ideal.

We next extract the `Spectra` object with the MS data from the data object and assign a new spectra variable with the employed collision energy, which we extract from the data object `sampleData`.

```
ms2_ctr <- spectra(lcms2)
ms2_ctr$collision_energy <-
    sampleData(lcms2)$fragmentation_mode[spectraSampleIndex(lcms2)]
```

We next filter the MS data by first restricting to MS2 spectra and then removing mass peaks from each spectrum with an intensity that is lower than 5% of the highest intensity for that spectrum, assuming that these very low intensity peaks represent background signal.

```
#' Remove low intensity peaks
low_int <- function(x, ...) {
    x > max(x, na.rm = TRUE) * 0.05
}

ms2_ctr <- filterMsLevel(ms2_ctr, 2L) |>
    filterIntensity(intensity = low_int)
```

We next remove also mass peaks with an m/z value greater or equal to the precursor m/z of the ion. This puts, for the later matching against reference spectra, more weight on the fragmentation pattern of ions and avoids hits based only on the precursor m/z peak (and hence a similar mass of the compared compounds). At last, we restrict the data to spectra with at least two fragment peaks and scale their intensities such that their sum is 1 for each spectrum. While similarity calculations are not affected by this scaling, it makes visual comparison of fragment spectra easier to read.

```
#' Remove precursor peaks and restrict to spectra with a minimum
#' number of peaks
ms2_ctr <- filterPrecursorPeaks(ms2_ctr, ppm = 50, mz = ">=")
ms2_ctr <- ms2_ctr[lengths(ms2_ctr) > 1] |>
    scalePeaks()
```

Finally, also to speed up the later comparison of the spectra against the reference database, we load the full MS data into memory (by changing the *backend* to `MsBackendMemory`) and *apply* all processing steps performed on this data so far. Keeping the MS data in memory has performance benefits, but is generally not suggested for large data sets. To evaluate the

impact for the present data set we print in addition the size of the data object before and after changing the backend.

```
#' Size of the object before loading into memory
print(object.size(ms2_ctr), units = "MB")
```

```
1.9 Mb
```

```
#' Load the MS data subset into memory
ms2_ctr <- setBackend(ms2_ctr, MsBackendMemory())
ms2_ctr <- applyProcessing(ms2_ctr)

#' Size of the object after loading into memory
print(object.size(ms2_ctr), units = "MB")
```

```
6 Mb
```

There is thus only a moderate increase in memory demand after loading the MS data into memory (also because we filtered and cleaned the MS2 data).

While we could proceed and match all these experimental MS2 spectra against reference fragment spectra, for our workflow we aim to annotate the features found significant in the differential abundance analysis. The goal is thus to identify the MS2 spectra from the second (LC-MS/MS) run that could represent fragments of the ions of features from the data in the first (LC-MS) run. Our approach is to match MS2 spectra against the significant features determined earlier based on their precursor m/z and retention time (given an acceptable tolerance) to the feature's m/z and retention time. This can be easily done using the `featureArea()` function that effectively considers the actual m/z and retention time ranges of the features' chromatographic peaks and therefore increase the chance of finding a correct match. This however also assumes that retention times between the first and second run don't differ by much. Alternatively, we would need to align the retention times of the second LC-MS/MS data set to those of the first.

Below we first extract the *feature area*, i.e., the m/z and retention time ranges, for the significant features.

```
#' Define the m/z and retention time ranges for the significant features
target <- featureArea(lcms1)[rownames(res_sig), ]
target
```

```
          mzmin    mzmax     rtmin     rtmax
FT0371 138.0544 138.0552 146.32270 152.86115
FT0565 161.0391 161.0407 159.00234 164.30799
FT0732 182.0726 182.0756  32.71242  42.28755
FT0845 195.0799 195.0887  30.73235  35.67337
FT1171 229.1282 229.1335 178.01450 183.35303
```

We next identify the fragment spectra with their precursor *m/z* and retention times within these ranges. We use the `filterRanges()` function that allows to filter a `Spectra` object using multiple ranges simultaneously. We apply this function separately to each feature (row in the above matrix) to extract the MS2 spectra representing fragmentation information of the presumed feature's ions.

```
#' Identify for each feature MS2 spectra with their precursor m/z and
#' retention time within the feature's m/z and retention time range
ms2_ctr_fts <- apply(target[, c("rtmin", "rtmax", "mzmin", "mzmax")],
                     MARGIN = 1, FUN = filterRanges, object = ms2_ctr,
                     spectraVariables = c("rtime", "precursorMz"))
lengths(ms2_ctr_fts)
```

```
FT0371 FT0565 FT0732 FT0845 FT1171
    11     12     46     21     12
```

The result from this `apply()` call is a `list` of `Spectra`, each element representing the result for one feature. With the exception of the last feature, multiple MS2 spectra could be identified. We next combine the `list` of `Spectra` into a single `Spectra` object using the `concatenateSpectra()` function and add an additional spectra variable containing the respective feature identifier.

```
l <- lengths(ms2_ctr_fts)
#' Combine the individual Spectra objects
ms2_ctr_fts <- concatenateSpectra(ms2_ctr_fts)
#' Assign the feature identifier to each MS2 spectrum
ms2_ctr_fts$feature_id <- rep(rownames(res_sig), l)

## Save for reuse in other vignettes
save(ms2_ctr_fts, file = "objects/spectra_significant_fts.RData")
```

We have now a `Spectra` object with fragment spectra for the significant features from our differential expression analysis.

This object can be used for annotation using various tools, see the vignette presenting how to process and annotate it using python tools [here](add later).

We next build our reference data which we need to process the same way as our *query* spectra. We extract all fragment spectra from the MassBank database, restrict to positive polarity data (since our experiment was acquired in positive polarity) and perform the same processing to the fragment spectra from the MassBank database.

```
ms2_ref <- Spectra(mb) |>
    filterPolarity(1L) |>
    filterIntensity(intensity = low_int) |>
    filterPrecursorPeaks(ppm = 50, mz = ">=")
ms2_ref <- ms2_ref[lengths(ms2_ref) > 1] |>
    scalePeaks()
```

Note that here we could switch to a `MsBackendMemory` backend hence loading the full data from the reference database into memory. This could have a positive impact on the performance of the subsequent spectra matching, however it would also increase the memory demand of the present analysis.

Now that both the `Spectra` object for the second run and the database spectra have been prepared, we can proceed with the matching process. We use the `matchSpectra()` function from the *MetaboAnnotation* package with the `CompareSpectraParam` to define the settings for the matching. With the following parameters:

- `requirePrecursor = TRUE`: Limits spectra similarity calculations to fragment spectra with a similar precursor m/z.
- `tolerance` and `ppm`: Defines the acceptable difference between compared m/z values. These relaxed tolerance settings ensure that we find matches even if reference spectra were acquired on instruments with a lower accuracy.
- `THRESHFUN`: Defines which matches to report. Here, we keep all matches resulting in a spectra similarity score (calculated with the normalized dot product (Stein and Scott 1994), the default similarity function) larger than 0.6.

```
register(SerialParam())
#' Define the settings for the spectra matching.
prm <- CompareSpectraParam(ppm = 40, tolerance = 0.05,
                           requirePrecursor = TRUE,
                           THRESHFUN = function(x) which(x >= 0.7))

ms2_mtch <- matchSpectra(ms2_ctr_fts, ms2_ref, param = prm)
ms2_mtch
```

```
Object of class MatchedSpectra
Total number of matches: 117
Number of query objects: 102 (10 matched)
Number of target objects: 69561 (14 matched)
```

Thus, from the total 102 query MS2 spectra, only 10 could be matched to (at least) one reference fragment spectrum.

Below we restrict the results to these matching spectra and extract all metadata of the query and target spectra as well as the similarity score (the complete list of available metadata information can be listed with the `colnames()` function).

```r
#' Keep only query spectra with matching reference spectra
ms2_mtch <- ms2_mtch[whichQuery(ms2_mtch)]

#' Extract the results
ms2_mtch_res <- matchedData(ms2_mtch)
nrow(ms2_mtch_res)
```

```
[1] 117
```

Now, we have query-target pairs with a spectra similarity higher than 0.7. Similar to the MS1-based annotation also this result table contains redundant information: we have multiple fragment spectra per feature and also MassBank contains several fragment spectra for each compound, measured using differing collision energies or MS instruments, by different laboratories. Below we thus iterate over the feature-compound pairs and select for each the one with the highest score. As an identifier for a compound, we use its fragment spectra's INCHI-key, since compound names in MassBank do not have accepted consensus/controlled vocabularies.

```r
#' - split the result per feature
#' - select for each feature the best matching result for each compound
#' - combine the result again into a data frame
ms2_mtch_res <-
    ms2_mtch_res |>
    split(f = paste(ms2_mtch_res$feature_id, ms2_mtch_res$target_inchikey)) |>
    lapply(function(z) {
        z[which.max(z$score), ]
    }) |>
    do.call(what = rbind) |>
    as.data.frame()
```

```r
#' List the best matching feature-compound pair
pandoc.table(ms2_mtch_res[, c("feature_id", "target_name", "score",
                              "target_inchikey")],
             style = "rmarkdown")
```

feature_id	target_name	score	target_inchikey
FT0845	Isocaffeine	0.8048	LPHGQDQBBGAPDZ-UHFFFAOYSA-N
FT0845	Caffeine	0.8926	NA
FT0845	Caffeine	0.9998	RYYVLZVUVIJVGH-UHFFFAOYSA-N

Table 14: MS2 annotation results.

Thus, from the 5 significant features, only one could be annotated to a compound based on the MS2-based approach. There could be many reasons for the failure to find matches for the other features. Although MS2 spectra were selected for each feature, most appear to only represent noise, as for most features, in the LC-MS/MS run, no or only very low MS1 signal was recorded, indicating that in that selected sample the original compound might not (or no longer) be present. Also, most reference databases contain predominantly fragment spectra for protonated (*[M+H]+*) ions of compounds, while some of the features might represent signal from other types of ions which would result in a different fragmentation pattern. Finally, fragment spectra of compounds of interest might also simply not be present in the used reference database.

Thus, combining the information from the MS1- and MS2 based annotation we can only annotate one feature with a considerable confidence. The feature with the m/z of 195.0879 and a retention time of 32 seconds seems to be an ion of caffeine. While the result is somewhat disappointing it also clearly shows the importance of proper experimental planning and the need to control for all potential confounding factors. For the present experiment, no disease-specific biomarker could be identified, but a life-style property of individuals suffering from this disease: coffee consumption was probably contraindicated to patients of the CVD group to reduce the risk of heart arrhythmia.

We below plot the EIC for this feature highlighting the retention time at which the highest scoring MS2 spectra was recorded and create a mirror plot comparing this MS2 spectra to the reference fragment spectra for caffeine.

```r
col_sample <- col_phenotype[sampleData(lcms1)$phenotype]
#' Extract and plot EIC for the annotated feature
eic <- featureChromatograms(lcms1, features = ms2_mtch_res$feature_id[1])
```

```
Processing chromatographic peaks for features
```

```r
plot(eic, col = col_sample, peakCol = col_sample[chromPeaks(eic)[, "sample"]],
     peakBg = paste0(col_sample[chromPeaks(eic)[, "sample"]], 20))
legend("topright", col = col_phenotype, legend = names(col_phenotype), lty = 1)

#' Identify the best matching query-target spectra pair
idx <- which.max(ms2_mtch_res$score)

#' Indicate the retention time of the MS2 spectrum in the EIC plot
abline(v = ms2_mtch_res$rtime[idx])
```

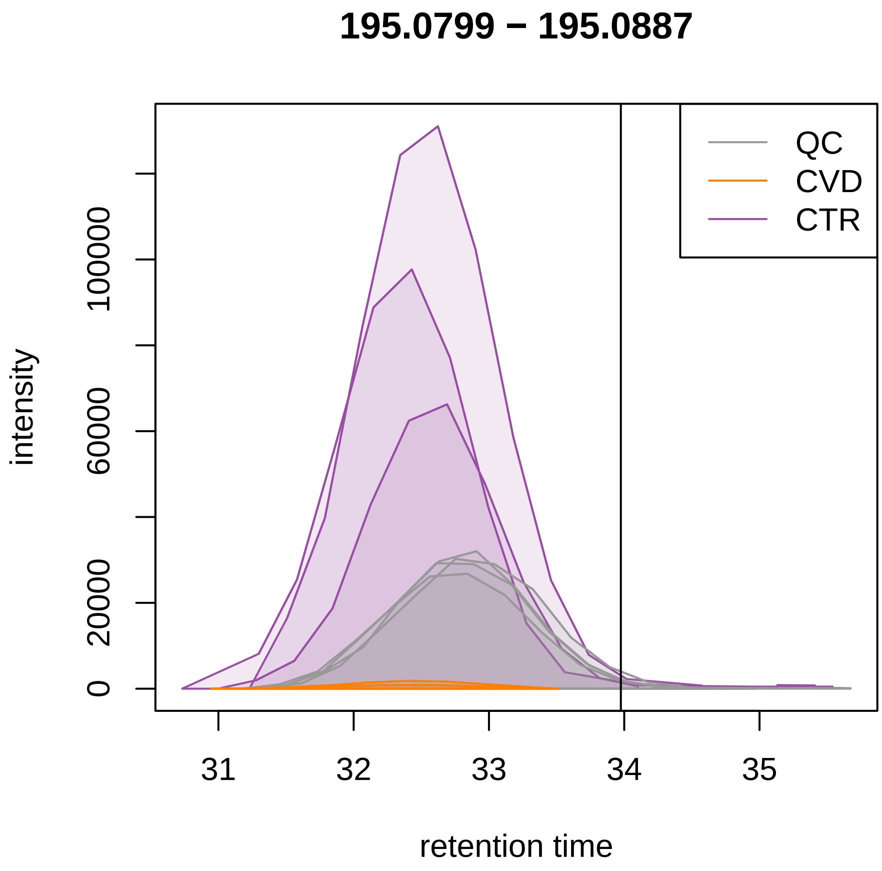

Figure 36: Extracted ion chromatograms and MS2 spectra for the annotated feature.

```r
#' Get the index of the MS2 spectrum in the query object
query_idx <- which(query(ms2_mtch)$.original_query_index ==
                                  ms2_mtch_res$.original_query_index[idx])
query_ms2 <- query(ms2_mtch)[query_idx]
#' Get the index of the MS2 spectrum in the target object
target_idx <- which(target(ms2_mtch)$spectrum_id ==
                                    ms2_mtch_res$target_spectrum_id[idx])
target_ms2 <- target(ms2_mtch)[target_idx]

#' Create a mirror plot comparing the two best matching spectra
plotSpectraMirror(query_ms2, target_ms2)
legend("topleft",
       legend = paste0("precursor m/z: ",
                       format(precursorMz(query_ms2), 3)))
```

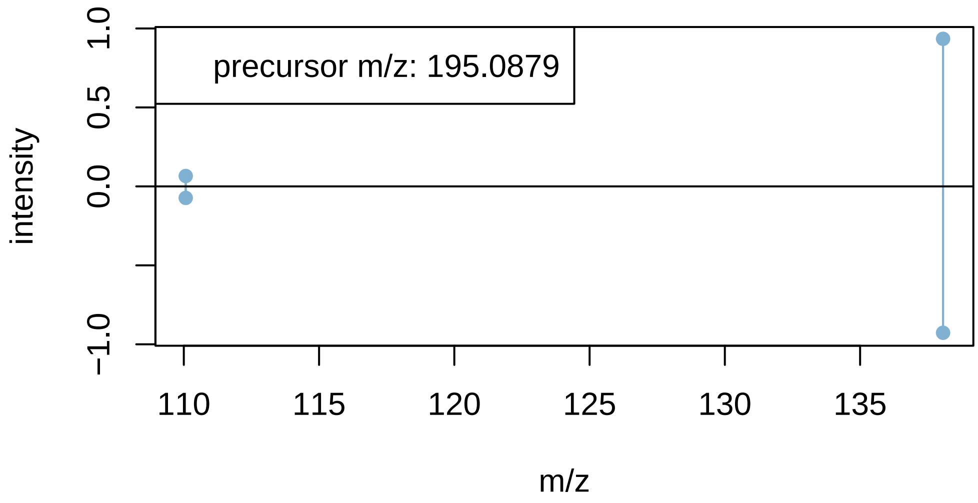

Figure 37: MS2 spectra for the annotated feature.

The plot above clearly shows the higher signal for this feature in CTR compared to CVD samples. The QC samples exhibit a lower but highly consistent signal, suggesting the absence of strong technical noise or biases in the raw data of this experiment. The vertical line indicates the retention time of the fragment spectrum with the best match against a reference spectrum. It has to be noted that, since the fragment spectra were measured in a separate LC-MS/MS

experiment, this should only be considered as an indication of the approximate retention time in which ions were fragmented in the second experiment. The fragment spectrum for this feature, shown in the upper panel of the right plot above is highly similar to the reference spectrum for caffeine from MassBank (shown in the lower panel). In addition to their matching precursor m/z, the same two fragments (same m/z and intensity) are present in both spectra.

We can also extract additional metadata for the matching reference spectrum, such as the used collision energy, fragmentation mode, instrument type, instrument as well as the ion (adduct) that was fragmented.

```
spectraData(target_ms2, c("collisionEnergy_text", "fragmentation_mode",
                          "instrument_type", "instrument", "adduct")) |>
    as.data.frame()
```

```
  collisionEnergy_text fragmentation_mode instrument_type
1         55 (nominal)                HCD     LC-ESI-ITFT
                            instrument adduct
1 LTQ Orbitrap XL Thermo Scientific [M+H]+
```

External tools or alternative annotation approaches

The present workflow highlights how annotation could be performed within R using packages from the Bioconductor project, but there are also other excellent external softwares that could be used as an alternative, such as SIRIUS (Dührkop et al. 2019), mummichog (Li et al. 2013) or GNPS (Nothias et al. 2020) among others. To use these, the data would need to be exported in a format supported by these. For MS2 spectra, the data could easily be exported in the required MGF file format using the *MsBackendMgf* Bioconductor package. Integration of *xcms* into feature-based molecular networking with GNPS is described in the GNPS documentation.

In alternative, or in addition, evidence for the potential matching chemical formula of a feature could be derived by evaluating the isotope pattern of its full MS1 scan. This could provide information on the isotope composition. Also for this, various functions such as the `isotopologues()` from the or *MetaboCoreUtils* package or the functionality of the *envipat* R package (Loos et al. 2015) could be used.

Summary

In this tutorial, we describe an end-to-end workflow for LC-MS-based untargeted metabolomics experiments, conducted entirely within R using packages from the Bioconductor project or base R functionality. While other excellent software exists to perform similar analyses, the power

of an R-based workflow lies in its adaptability to individual data sets or research questions and its ability to build reproducible workflows and documentation.

Due to space restrictions we don't provide a comprehensive listing of methodologies for the individual analysis steps. More advanced options or approaches would be available, e.g., for normalization of the data, which will however also be heavily dependent on the size and properties of the analyzed data set, as well as for annotation of the features.

As a result, we found in the present analysis a set of features with significant abundance differences between the compared groups. From these we could however only reliably annotate a single feature, that related to the lifestyle of individuals rather then to the pathological properties of the investigated disease. This low proportion of annotated signals is however not uncommon for untargeted metabolomics experiments and reflects the need for more comprehensive and reliable reference annotation libraries.

Session information

```r
sessionInfo()
```

```
R version 4.5.2 (2025-10-31)
Platform: x86_64-pc-linux-gnu
Running under: Ubuntu 24.04.3 LTS

Matrix products: default
BLAS:   /usr/lib/x86_64-linux-gnu/openblas-pthread/libblas.so.3
LAPACK: /usr/lib/x86_64-linux-gnu/openblas-pthread/libopenblasp-r0.3.26.so;  LAPACK version 3

locale:
 [1] LC_CTYPE=en_US.UTF-8       LC_NUMERIC=C
 [3] LC_TIME=en_US.UTF-8        LC_COLLATE=en_US.UTF-8
 [5] LC_MONETARY=en_US.UTF-8    LC_MESSAGES=en_US.UTF-8
 [7] LC_PAPER=en_US.UTF-8       LC_NAME=C
 [9] LC_ADDRESS=C               LC_TELEPHONE=C
[11] LC_MEASUREMENT=en_US.UTF-8 LC_IDENTIFICATION=C

time zone: Etc/UTC
tzcode source: system (glibc)

attached base packages:
[1] stats4    stats     graphics  grDevices utils     datasets  methods
[8] base
```

```
other attached packages:
 [1] UpSetR_1.4.0                ggVennDiagram_1.5.4
 [3] MetaboAnnotation_1.14.0     CompoundDb_1.14.0
 [5] AnnotationFilter_1.34.0     AnnotationHub_4.0.0
 [7] BiocFileCache_3.0.0         dbplyr_2.5.1
 [9] gridExtra_2.3               ggfortify_0.4.19
[11] ggplot2_4.0.0               vioplot_0.5.1
[13] zoo_1.8-14                  sm_2.2-6.0
[15] pheatmap_1.0.13             RColorBrewer_1.1-3
[17] pander_0.6.6                limma_3.66.0
[19] MetaboCoreUtils_1.18.0      xcms_4.8.0
[21] MsBackendMetaboLights_1.4.2 Spectra_1.20.0
[23] BiocParallel_1.44.0         alabaster.se_1.10.0
[25] alabaster.base_1.10.0       SummarizedExperiment_1.40.0
[27] Biobase_2.70.0              GenomicRanges_1.62.0
[29] Seqinfo_1.0.0               IRanges_2.44.0
[31] S4Vectors_0.48.0            BiocGenerics_0.56.0
[33] generics_0.1.4              MatrixGenerics_1.22.0
[35] matrixStats_1.5.0           MsIO_0.0.11
[37] MsExperiment_1.12.0         ProtGenerics_1.42.0
[39] readxl_1.4.5                BiocStyle_2.38.0
[41] quarto_1.5.1                knitr_1.50

loaded via a namespace (and not attached):
  [1] later_1.4.4                 bitops_1.0-9
  [3] filelock_1.0.3              tibble_3.3.0
  [5] cellranger_1.1.0            preprocessCore_1.72.0
  [7] XML_3.99-0.20               lifecycle_1.0.4
  [9] httr2_1.2.1                 doParallel_1.0.17
 [11] processx_3.8.6              lattice_0.22-7
 [13] MASS_7.3-65                 MultiAssayExperiment_1.36.0
 [15] magrittr_2.0.4              rmarkdown_2.30
 [17] yaml_2.3.10                 MsCoreUtils_1.21.0
 [19] DBI_1.2.3                   abind_1.4-8
 [21] purrr_1.1.0                 RCurl_1.98-1.17
 [23] rappdirs_0.3.3              MSnbase_2.36.0
 [25] ncdf4_1.24                  codetools_0.2-20
 [27] DelayedArray_0.36.0         DT_0.34.0
 [29] xml2_1.4.1                  tidyselect_1.2.1
 [31] farver_2.1.2                base64enc_0.1-3
 [33] jsonlite_2.0.0              iterators_1.0.14
 [35] foreach_1.5.2               tools_4.5.2
```

```
 [37] progress_1.2.3           Rcpp_1.1.0
 [39] glue_1.8.0               SparseArray_1.10.1
 [41] BiocBaseUtils_1.12.0     xfun_0.54
 [43] dplyr_1.1.4              HDF5Array_1.38.0
 [45] withr_3.0.2              BiocManager_1.30.26
 [47] fastmap_1.2.0            rhdf5filters_1.22.0
 [49] digest_0.6.37            R6_2.6.1
 [51] rsvg_2.7.0               dichromat_2.0-0.1
 [53] RSQLite_2.4.4            h5mread_1.2.0
 [55] tidyr_1.3.1              data.table_1.17.8
 [57] prettyunits_1.2.0        PSMatch_1.14.0
 [59] httr_1.4.7               htmlwidgets_1.6.4
 [61] S4Arrays_1.10.0          pkgconfig_2.0.3
 [63] gtable_0.3.6             blob_1.2.4
 [65] S7_0.2.0                 impute_1.84.0
 [67] MassSpecWavelet_1.76.0   XVector_0.50.0
 [69] htmltools_0.5.8.1        MALDIquant_1.22.3
 [71] clue_0.3-66              scales_1.4.0
 [73] alabaster.matrix_1.10.0  png_0.1-8
 [75] rstudioapi_0.17.1        reshape2_1.4.4
 [77] rjson_0.2.23             curl_7.0.0
 [79] cachem_1.1.0             rhdf5_2.54.0
 [81] stringr_1.5.2            BiocVersion_3.22.0
 [83] parallel_4.5.2           AnnotationDbi_1.72.0
 [85] mzID_1.48.0              vsn_3.78.0
 [87] pillar_1.11.1            grid_4.5.2
 [89] alabaster.schemas_1.10.0 vctrs_0.6.5
 [91] MsFeatures_1.18.0        pcaMethods_2.2.0
 [93] cluster_2.1.8.1          evaluate_1.0.5
 [95] cli_3.6.5                compiler_4.5.2
 [97] rlang_1.1.6              crayon_1.5.3
 [99] labeling_0.4.3           QFeatures_1.20.0
[101] ChemmineR_3.62.0         ps_1.9.1
[103] affy_1.88.0              plyr_1.8.9
[105] fs_1.6.6                 stringi_1.8.7
[107] Biostrings_2.78.0        lazyeval_0.2.2
[109] Matrix_1.7-4             hms_1.1.4
[111] bit64_4.6.0-1            Rhdf5lib_1.32.0
[113] KEGGREST_1.50.0          statmod_1.5.1
[115] alabaster.ranges_1.10.0  mzR_2.44.0
[117] igraph_2.2.1             memoise_2.0.1
[119] affyio_1.80.0            bit_4.6.0
```

Acknowledgment

Thanks to Steffen Neumann for his continuous work to develop and maintain the xcms software. And the Bioconductor and RforMassSpectrometry community for their continuous support and development of the software used in this tutorial.

References

Broadhurst, David, Royston Goodacre, Stacey N. Reinke, Julia Kuligowski, Ian D. Wilson, Matthew R. Lewis, and Warwick B. Dunn. 2018. "Guidelines and Considerations for the Use of System Suitability and Quality Control Samples in Mass Spectrometry Assays Applied in Untargeted Clinical Metabolomic Studies." *Metabolomics : Official Journal of the Metabolomic Society* 14 (6): 72. https://doi.org/10.1007/s11306-018-1367-3.

Chambers, Matthew C, Brendan Maclean, Robert Burke, Dario Amodei, Daniel L Ruderman, Steffen Neumann, Laurent Gatto, et al. 2012. "A Cross-Platform Toolkit for Mass Spectrometry and Proteomics." *Nature Biotechnology* 30 (10): 918–20. https://doi.org/10.1038/nbt.2377.

De Livera, Alysha M., Daniel A. Dias, David De Souza, Thusitha Rupasinghe, James Pyke, Dedreia Tull, Ute Roessner, Malcolm McConville, and Terence P. Speed. 2012. "Normalizing and Integrating Metabolomics Data." *Analytical Chemistry* 84 (24): 10768–76. https://doi.org/10.1021/ac302748b.

Dührkop, Kai, Markus Fleischauer, Marcus Ludwig, Alexander A. Aksenov, Alexey V. Melnik, Marvin Meusel, Pieter C. Dorrestein, Juho Rousu, and Sebastian Böcker. 2019. "SIRIUS 4: A Rapid Tool for Turning Tandem Mass Spectra into Metabolite Structure Information." *Nature Methods* 16 (4): 299–302. https://doi.org/10.1038/s41592-019-0344-8.

Klåvus, Anton, Marietta Kokla, Stefania Noerman, Ville M. Koistinen, Marjo Tuomainen, Iman Zarei, Topi Meuronen, et al. 2020. "'Notame': Workflow for Non-Targeted LC–MS Metabolic Profiling." *Metabolites* 10 (4): 135. https://doi.org/10.3390/metabo10040135.

Li, Shuzhao, Youngja Park, Sai Duraisingham, Frederick H. Strobel, Nooruddin Khan, Quinlyn A. Soltow, Dean P. Jones, and Bali Pulendran. 2013. "Predicting Network Activity from High Throughput Metabolomics." *PLoS Computational Biology* 9 (7): e1003123. https://doi.org/10.1371/journal.pcbi.1003123.

Loos, Martin, Christian Gerber, Francesco Corona, Juliane Hollender, and Heinz Singer. 2015. "Accelerated Isotope Fine Structure Calculation Using Pruned Transition Trees." *Analytical Chemistry* 87 (11): 5738–44. https://doi.org/10.1021/acs.analchem.5b00941.

Nothias, Louis-Félix, Daniel Petras, Robin Schmid, Kai Dührkop, Johannes Rainer, Abinesh Sarvepalli, Ivan Protsyuk, et al. 2020. "Feature-Based Molecular Networking in the GNPS Analysis Environment." *Nature Methods* 17 (9): 905–8. https://doi.org/10.1038/s41592-020-0933-6.

Rainer, Johannes, Andrea Vicini, Liesa Salzer, Jan Stanstrup, Josep M. Badia, Steffen Neumann, Michael A. Stravs, et al. 2022. "A Modular and Expandable Ecosystem for

Metabolomics Data Annotation in R." *Metabolites* 12 (2): 173. https://doi.org/10.3390/metabo12020173.

Schymanski, Emma L., Junho Jeon, Rebekka Gulde, Kathrin Fenner, Matthias Ruff, Heinz P. Singer, and Juliane Hollender. 2014. "Identifying Small Molecules via High Resolution Mass Spectrometry: Communicating Confidence." *Environmental Science & Technology* 48 (4): 2097–98. https://doi.org/10.1021/es5002105.

Smith, Colin A., Elizabeth J. Want, Grace O'Maille, Ruben Abagyan, and Gary Siuzdak. 2006. "XCMS: Processing Mass Spectrometry Data for Metabolite Profiling Using Nonlinear Peak Alignment, Matching, and Identification." *Analytical Chemistry* 78 (3): 779–87. https://doi.org/10.1021/ac051437y.

Smyth, Gordon K. 2004. "Linear Models and Empirical Bayes Methods for Assessing Differential Expression in Microarray Experiments." *Statistical Applications in Genetics and Molecular Biology* 3: Art. 3, 29 pp. (electronic).

Stein, Stephen E., and Donald R. Scott. 1994. "Optimization and Testing of Mass Spectral Library Search Algorithms for Compound Identification." *Journal of the American Society for Mass Spectrometry* 5 (9): 859–66. https://doi.org/10.1021/jasms.8b00613.

Sumner, Lloyd W., Alexander Amberg, Dave Barrett, Michael H. Beale, Richard Beger, Clare A. Daykin, Teresa W.-M. Fan, et al. 2007. "Proposed Minimum Reporting Standards for Chemical Analysis Chemical Analysis Working Group (CAWG) Metabolomics Standards Initiative (MSI)." *Metabolomics : Official Journal of the Metabolomic Society* 3 (3): 211–21. https://doi.org/10.1007/s11306-007-0082-2.

Tautenhahn, Ralf, Christoph Böttcher, and Steffen Neumann. 2008. "Highly Sensitive Feature Detection for High Resolution LC/MS." *BMC Bioinformatics* 9 (1): 504. https://doi.org/10.1186/1471-2105-9-504.
