# Supplementary material for: xcms in Peak Form: Now Anchoring a Complete Metabolomics Data Preprocessing and Analysis Software Ecosystem
Source: Anal Chem. 2025 Dec 8;97(50):27639–45. doi: 10.1021/acs.analchem.5c04338 (PMC12750407; doi:10.1021/acs.analchem.5c04338)
Supplement: Supplementary file 3 [file ac5c04338_si_003.pdf]

xcms-based preprocessing of a large LC-MS/MS data set; xcms version 4

Supporting Information for: ***xcms in peak form: Now anchoring a complete metabolomics data preprocessing and analysis software ecosystem***

Philippine Louail[1,2], Carl Brunius[3], Mar Garcia-Aloy[4], William Kumler[5], Norman Storz[6], Jan Stanstrup[7], Hendrik Treutler[6], Pablo Vangeenderhuysen[8], Michael Witting[9,10], Steffen Neumann[6,11,*], Johannes Rainer[1]

[1]Institute for Biomedicine, Eurac Research, 39100, Bolzano, Italy.
[2]Chair for Bioinformatics, Friedrich-Schiller-University Jena, 07743, Jena, Germany.
[3]Department of Life Sciences, Food and Nutrition Science, Chalmers University of Technology, SE-412 96 Göte-borg, Sweden.
[4]Metabolomics Unit, Research and Innovation Centre, Fondazione Edmund Mach, 38098, San Michele all'Adige (TN), Italy.
[5]School of Oceanography, University of Washington, Seattle, WA, 98195, USA.
[6]Leibniz Institute of Plant Biochemistry, MetaCom, Weinberg 3, 06120 Halle, Germany.
[7]Department of Nutrition, Exercise and Sports, University of Copenhagen, Rolighedsvej 26, 1958 Frederiksberg C, Denmark.
[8]Laboratory of Integrative Metabolomics (LIMET), Ghent University, 9820 Merelbeke, Belgium.
[9]Metabolomics and Proteomics Core, Helmholtz Zentrum München, Ingolstädter Landstraße 1, 85764 Neuherberg, Germany.
[10]Chair of Analytical Food Chemistry, TUM School of Life Sciences, Technical University of Munich, Maximus-von-Imhof-Forum 2, 85354 Freising-Weihenstephan, Germany.
[11]German Centre for Integrative Biodiversity Research (iDiv) Halle-Jena-Leipzig, Puschstraße 4, 04103 Leipzig, Germany.
[*]corresponding author: sneumann@ipb-halle.de

Abstract

This document describes the preprocessing of a large untargeted metabolomics dataset consisting of about 1,000 samples using the *xcms* package (Louail, Brunius, et al. 2025) and export of its results for molecular networking with GNPS. For the present analysis, *xcms* version 4.8, part of Bioconductor release 3.22, was used. To evaluate the performance, memory usage and timing of function calls are recorded during the analysis.

Introduction

In this document we perform the preprocessing of a large untargeted metabolomics dataset using the *xcms* package (Louail, Brunius, et al. 2025) and record memory usage and timing of function calls during the analysis. The dataset consists of approximately **1,000 samples** (mzXML) files which can be downloaded from the *MassIVE* repository. The accession ID of the data set is *MSV000080030*. Preprocessing of this data set was also performed in (Nothias et al. 2020) to exemplify large-scale analysis with *xcms* in the framework of the GNPS environment.

Note that the settings for the individual preprocessing steps were not optimized for the present data set. For information on how to derive settings specific for a data set see the Metabonaut resource (Louail, Graeve, et al. 2025).

Data import

It is assumed that the mzXML files were downloaded from MassIVE and the *MSV000080030* folder was placed within the same directory this quarto document resides. We first load all required libraries and configure the parallel processing setup. For the present analysis we use 4 parallel processes. To track memory usage and duration of the various function calls we use the `peakRAM()` function from the same-named R package. Note however that this function fails to properly track memory usage in R if parallel processing is used.

```
library(MsExperiment)
library(Spectra)
library(xcms)
library(peakRAM)
```

Setup parallel processing.

```
NCORES <- 4
register(MulticoreParam(NCORES), default = TRUE)
```

We next define all data files of the project, drop measurements of blanks and import the data as a `MsExperiment` object.

```
fls <- dir("MSV000080030", pattern = "mzXML$", full.names = TRUE,
           recursive = TRUE)
fls <- fls[-grep(fls, pattern = "/Blank", fixed = TRUE)]
pr <- peakRAM(
    mse <- readMsExperiment(fls)
)
```

The data set consists of in total 1039 files with in total 5101430 spectra, 1020035 MS1 and 4081395 MS2 spectra. The size of the object in memory is shown below. Note that only spectra metadata is loaded into memory, while the mass peak data is retrieved on-the-fly from the original data files.

```
print(object.size(mse), units = "GB")
```

```
1 Gb
```

Chromatographic peak detection

Chromatographic peak detection is performed using the *CentWave* algorithm using the same seetings as in (Nothias et al. 2020).

```
cwp <- CentWaveParam(snthresh = 3, noise = 800, peakwidth = c(2, 30),
                     ppm = 20)
tmp <- peakRAM(
    xmse <- findChromPeaks(mse, param = cwp, chunkSize = NCORES * 2)
)
pr <- rbind(pr, tmp)
```

In total 4105804 chromatographic peaks were identified. We next perform the peak refinement to reduce peak detection artifacts. This step was not performed in the original analysis. It was developed to improve the output of the chromatographic peak detection.

```
tmp <- peakRAM(refineChromPeaks(xmse, MergeNeighboringPeaksParam(),
                                chunkSize = NCORES * 2))
```

```
Reduced from 4105804 to 4011070 chromatographic peaks.
```

```
pr <- rbind(pr, tmp)
```

After peak refinement, 4105804 chromatographic peaks are present.

The size of the result object in memory is:

```
print(object.size(xmse), units = "GB")
```

```
1.9 Gb
```

Alignment

Prior retention time alignment we need to perform an initial correspondence analysis. Among the such defined features (i.e., chromatographic peaks grouped across samples) *anchor peaks* will be defined that are used to reduce retention time shifts and hence align the samples.

```
pdp <- PeakDensityParam(sampleGroups = rep(1, length(xmse)),
                        bw = 2, binSize = 0.1, minFraction = 0.5)
tmp <- peakRAM(
    xmse <- groupChromPeaks(xmse, param = pdp)
)
pr <- rbind(pr, tmp)
```

Next we perform the retention time alignment.

```
pgp <- PeakGroupsParam(minFraction = 0.7, extraPeaks = 100, span = 0.3)
tmp <- peakRAM(
    xmse <- adjustRtime(xmse, param = pgp)
)
```

```
Performing retention time alignment using 387 anchor peaks.
```

```
Warning: Adjusted retention times had to be re-adjusted for some files to
ensure them being in the same order than the raw retention times. A call to
'dropAdjustedRtime' might thus fail to restore retention times of
chromatographic peaks to their original values. Eventually consider to increase
the value of the 'span' parameter.
```

```
pr <- rbind(pr, tmp)
```

The size of the result object in memory is:

```
print(object.size(xmse), units = "GB")
```

```
1.9 Gb
```

Correspondence analysis

We next perform the correspondence analysis.

```
pdp <- PeakDensityParam(sampleGroups = rep(1, length(xmse)),
                        bw = 2, binSize = 0.015, minFraction = 0.03)
tmp <- peakRAM(
    xmse <- groupChromPeaks(xmse, param = pdp)
)
pr <- rbind(pr, tmp)
```

The total number of defined LC-MS features are 19936.

The size of the result object in memory is:

```
print(object.size(xmse), units = "GB")
```

```
2 Gb
```

Gap filling

As a last step in the preprocessing, we perform the gap-filling to reduce the number of missing values.

```
tmp <- peakRAM(
    xmse <- fillChromPeaks(xmse, param = ChromPeakAreaParam(),
                           chunkSize = NCORES * 2)
)
pr <- rbind(pr, tmp)
```

The size of the result object in memory is:

```
print(object.size(xmse), units = "GB")
```

```
5.7 Gb
```

Summary of preprocessing performance

The time elapse for the individual preprocessing steps along with memory usage is shown in the table below. Note that on parallel processing setups the memory usage is not tracked properly by the `peakRAM()` function.

```
library(pander)
pr$Elapsed_Time_sec <- pr$Elapsed_Time_sec / 60
colnames(pr)[colnames(pr) == "Elapsed_Time_sec"] <- "Elapsed_Time_minutes"
pandoc.table(pr, style = "rmarkdown")
```

Table 1: Table continues below

Function_Call
mse<-readMsExperiment(fls)
xmse<-findChromPeaks(mse,param=cwp,chunkSize=NCORES*2)
refineChromPeaks(xmse,MergeNeighboringPeaksParam(),chunkSize=NCORES*2)
xmse<-groupChromPeaks(xmse,param=pdp)
xmse<-adjustRtime(xmse,param=pgp)
xmse<-groupChromPeaks(xmse,param=pdp)
xmse<-fillChromPeaks(xmse,param=ChromPeakAreaParam(),chunkSize=NCORES*2)

Elapsed_Time_minutes	Total_RAM_Used_MiB	Peak_RAM_Used_MiB
6.471	974.4	2638
98.27	477.2	3710
14.58	469.3	3233
0.3942	7.4	3114
0.8989	67	3201
1.588	25.7	2630
138.6	2171	6083

```
dr <- "xcms-preprocessing"
dir.create(dr, showWarnings = FALSE)
save(xmse, file = file.path(dr, "xmse.RData"))
```

Prepare and export data for GNPS

We next identify all MS2 spectra for all defined LC-MS features and export these, along with the feature abundances for use in the GNPS environment as described in (Nothias et al.

2020).

We first extract the feature values from the result object.

```
fall <- featureDefinitions(xmse)[, c("mzmin", "mzmed", "mzmax",
                                      "rtmin", "rtmed", "rtmax")]
fall <- cbind(feature_id = rownames(fall),
              fall,
              featureValues(xmse, value = "into"))
write.table(fall, file.path(dr, "xcms_all_features.txt"), sep = "\t",
            quote = FALSE, row.names = FALSE)
```

Next we extract all MS2 spectra associated with any of the features' detected chromatographic peaks. Note that we disable parallel processing for this step, because we would run out of memory on the computer setup used.

```
source("https://raw.githubusercontent.com/jorainer/xcms-gnps-tools/master/customFunctions.R")
bpstop()
register(SerialParam())
pr_ms2 <- peakRAM(
    ms2_all <- featureSpectra(xmse, skipFilled = TRUE)
)
```

In total 1489671 MS2 spectra were identified and extracted for the 19936 LC-MS features. We also clean and re-format the extracted MS2 spectra for their use in GNPS.

```
## ms2_all <- filterIntensity(ms2_all, intensity = 0)
## ms2_all <- filterEmptySpectra(ms2_all)
ms2_all <- formatSpectraForGNPS(ms2_all)
```

We next select a single MS2 spectrum for each feature and export this reduced set also as an .mgf file. We use the `Spectra::combineSpectra()` function to *combine* spectra with the same value for parameter `f` into one. In the example below we use the `maxTicPeaksData()` function from the xcms-gnps-tools repository to report for each feature only the fragment peaks matrix with the largest TIC. As an alternative, the `combineSpectra()` method would also allow to create a *representative* spectrum combining all fragment peaks or reporting fragment peaks present in e.g. 80% of related fragment spectra (see next section for an example). The sets of fragment spectra assigned to a feature have to be declared with parameter `f` (which in our example is set to `f = feature_ms2$FEATURE_ID`, defining for each spectrum the feature it is related to). In addition, we have to change parameter `p` (which defines how to split and process the `Spectra` object in parallel) from the default `p = dataStorage(object)` to `p = rep(1, length(feature_ms2))` hence switching from a per-mzML-file-based processing to a

processing that allows to select a single representative fragment spectrum for feature across all data files.

```
tmp <- peakRAM(
    ms2_maxtic <- combineSpectra(
        ms2_all, f = ms2_all$FEATURE_ID,
        p = rep(1, length(ms2_all)), FUN = maxTicPeaksData)
)
pr_ms2 <- rbind(pr_ms2, tmp)
spectraNames(ms2_maxtic) <- paste0(ms2_maxtic$FEATURE_ID, " maxTic")
```

Next we export the data to a file which can then be submitted to GNPS feature-based molecular networking.

```
library(MsBackendMgf)
tmp <- peakRAM(
    export(backend = MsBackendMgf(),
           ms2_maxtic,
           file = file.path(dr, "ms2spectra_maxTic.mgf"))
)
pr_ms2 <- rbind(pr_ms2, tmp)
```

Summary of data export performance

The time elapse for the individual result extraction, formatting for GNPS and export steps along with memory usage is shown in the table below.

```
pr_ms2$Elapsed_Time_sec <- pr_ms2$Elapsed_Time_sec / 60
colnames(pr_ms2)[colnames(pr_ms2) == "Elapsed_Time_sec"] <-
    "Elapsed_Time_minutes"
pandoc.table(pr_ms2, style = "rmarkdown")
```

Table 3: Table continues below

Function_Call
ms2_all<-featureSpectra(xmse,skipFilled=TRUE)
ms2_maxtic<-combineSpectra(ms2_all,f=ms2_all$FEATURE_ID,p=rep(1,length(ms2_all)),FUN=maxTicPeaksData)
export(backend=MsBackendMgf(),ms2_maxtic,file=file.path(dr,“ms2spectra_maxTic.mgf”))

Elapsed_Time_minutes	Total_RAM_Used_MiB	Peak_RAM_Used_MiB
2.141	352.4	4292
14.96	286.2	16712
0.7359	128	2941

Session and computation information

The present analysis was performed using the Bioconductor docker images for the developmental version 3.22. The analysis was run on 4 CPUs of a Framework 13' notebook with a 13th Gen Intel(R) Core(TM) i7-1370P CPU (20 cores) and 64 GB of main memory.

```r
sessionInfo()
```

```
R version 4.5.2 (2025-10-31)
Platform: x86_64-pc-linux-gnu
Running under: Ubuntu 24.04.3 LTS

Matrix products: default
BLAS:   /usr/lib/x86_64-linux-gnu/openblas-pthread/libblas.so.3
LAPACK: /usr/lib/x86_64-linux-gnu/openblas-pthread/libopenblasp-r0.3.26.so;  LAPACK version 3

locale:
 [1] LC_CTYPE=en_US.UTF-8       LC_NUMERIC=C
 [3] LC_TIME=en_US.UTF-8        LC_COLLATE=en_US.UTF-8
 [5] LC_MONETARY=en_US.UTF-8    LC_MESSAGES=en_US.UTF-8
 [7] LC_PAPER=en_US.UTF-8       LC_NAME=C
 [9] LC_ADDRESS=C               LC_TELEPHONE=C
[11] LC_MEASUREMENT=en_US.UTF-8 LC_IDENTIFICATION=C

time zone: Etc/UTC
tzcode source: system (glibc)

attached base packages:
[1] stats4    stats     graphics  grDevices utils     datasets  methods
[8] base

other attached packages:
 [1] MsBackendMgf_1.18.0 pander_0.6.6        peakRAM_1.0.2
 [4] xcms_4.8.0          Spectra_1.20.0      BiocParallel_1.44.0
 [7] S4Vectors_0.48.0    BiocGenerics_0.56.0 generics_0.1.4
```

```
[10] MsExperiment_1.12.0 ProtGenerics_1.42.0 BiocStyle_2.38.0

loaded via a namespace (and not attached):
 [1] tidyselect_1.2.1            dplyr_1.1.4
 [3] farver_2.1.2                S7_0.2.0
 [5] fastmap_1.2.0               lazyeval_0.2.2
 [7] MassSpecWavelet_1.76.0      XML_3.99-0.20
 [9] digest_0.6.37               lifecycle_1.0.4
[11] cluster_2.1.8.1             statmod_1.5.1
[13] magrittr_2.0.4              compiler_4.5.2
[15] progress_1.2.3              rlang_1.1.6
[17] tools_4.5.2                 igraph_2.2.1
[19] yaml_2.3.10                 data.table_1.17.8
[21] knitr_1.50                  prettyunits_1.2.0
[23] S4Arrays_1.10.0             DelayedArray_0.36.0
[25] plyr_1.8.9                  RColorBrewer_1.1-3
[27] abind_1.4-8                 purrr_1.2.0
[29] grid_4.5.2                  preprocessCore_1.72.0
[31] ggplot2_4.0.0               iterators_1.0.14
[33] scales_1.4.0                MASS_7.3-65
[35] MultiAssayExperiment_1.36.0 dichromat_2.0-0.1
[37] SummarizedExperiment_1.40.0 cli_3.6.5
[39] crayon_1.5.3                mzR_2.44.0
[41] rmarkdown_2.30              reshape2_1.4.5
[43] BiocBaseUtils_1.12.0        ncdf4_1.24
[45] DBI_1.2.3                   affy_1.88.0
[47] stringr_1.6.0               parallel_4.5.2
[49] impute_1.84.0               AnnotationFilter_1.34.0
[51] BiocManager_1.30.26         XVector_0.50.0
[53] vsn_3.78.0                  matrixStats_1.5.0
[55] vctrs_0.6.5                 Matrix_1.7-4
[57] jsonlite_2.0.0              hms_1.1.4
[59] IRanges_2.44.0              MALDIquant_1.22.3
[61] clue_0.3-66                 foreach_1.5.2
[63] limma_3.66.0                tidyr_1.3.1
[65] affyio_1.80.0               glue_1.8.0
[67] MSnbase_2.36.0              codetools_0.2-20
[69] QFeatures_1.20.0            stringi_1.8.7
[71] gtable_0.3.6                GenomicRanges_1.62.0
[73] mzID_1.48.0                 tibble_3.3.0
[75] pillar_1.11.1               MsFeatures_1.18.0
[77] pcaMethods_2.2.0            htmltools_0.5.8.1
[79] Seqinfo_1.0.0               R6_2.6.1
```

```
[81] doParallel_1.0.17         evaluate_1.0.5
[83] lattice_0.22-7            Biobase_2.70.0
[85] MetaboCoreUtils_1.18.0    Rcpp_1.1.0
[87] PSMatch_1.14.0            SparseArray_1.10.1
[89] xfun_0.54                 MsCoreUtils_1.21.0
[91] fs_1.6.6                  MatrixGenerics_1.22.0
[93] pkgconfig_2.0.3
```

References

Louail, Philippine, Carl Brunius, Mar Garcia-Aloy, William Kumler, Norman Storz, Jan Stanstrup, Hendrik Treutler, et al. 2025. "Xcms at 20 and Still in Peak Form: Anchoring a Complete Metabolomics Data Preprocessing and Analysis Software Ecosystem." ChemRxiv. https://doi.org/10.26434/chemrxiv-2025-2n2kh.

Louail, Philippine, Marilyn De Graeve, Anna Tagliaferri, Vinicius Verri Hernandes, Daniel Marques de Sá e Silva, and Johannes Rainer. 2025. "Rformassspectrometry/Metabonaut: V1.2.0." Zenodo. https://doi.org/10.5281/zenodo.15554287.

Nothias, Louis-Félix, Daniel Petras, Robin Schmid, Kai Dührkop, Johannes Rainer, Abinesh Sarvepalli, Ivan Protsyuk, et al. 2020. "Feature-Based Molecular Networking in the GNPS Analysis Environment." *Nature Methods* 17 (9): 905–8. https://doi.org/10.1038/s41592-020-0933-6.
