# Supplementary material for: xcms in Peak Form: Now Anchoring a Complete Metabolomics Data Preprocessing and Analysis Software Ecosystem
Source: Anal Chem. 2025 Dec 8;97(50):27639–45. doi: 10.1021/acs.analchem.5c04338 (PMC12750407; doi:10.1021/acs.analchem.5c04338)
Supplement: Supplementary file 4 [file ac5c04338_si_004.pdf]

Large Scale Data Preprocessing with *xcms*

Supplementary Information for: *xcms in peak form: Now anchoring a complete metabolomics data preprocessing and analysis software ecosystem*

Philippine Louail[1,2], Carl Brunius[3], Mar Garcia-Aloy[4], William Kumler[5], Norman Storz[6], Jan Stanstrup[7], Hendrik Treutler[6], Pablo Vangeenderhuysen[8], Michael Witting[9,10], Steffen Neumann[6,11,*], Johannes Rainer[1]

[1]Institute for Biomedicine, Eurac Research, 39100, Bolzano, Italy.
[2]Chair for Bioinformatics, Friedrich-Schiller-University Jena, 07743, Jena, Germany.
[3]Department of Life Sciences, Food and Nutrition Science, Chalmers University of Technology, SE-412 96 Göte-borg, Sweden.
[4]Metabolomics Unit, Research and Innovation Centre, Fondazione Edmund Mach, 38098, San Michele all'Adige (TN), Italy.
[5]School of Oceanography, University of Washington, Seattle, WA, 98195, USA.
[6]Leibniz Institute of Plant Biochemistry, MetaCom, Weinberg 3, 06120 Halle, Germany.
[7]Department of Nutrition, Exercise and Sports, University of Copenhagen, Rolighedsvej 26, 1958 Frederiksberg C, Denmark.
[8]Laboratory of Integrative Metabolomics (LIMET), Ghent University, 9820 Merelbeke, Belgium.
[9]Metabolomics and Proteomics Core, Helmholtz Zentrum München, Ingolstädter Landstraße 1, 85764 Neuherberg, Germany.
[10]Chair of Analytical Food Chemistry, TUM School of Life Sciences, Technical University of Munich, Maximus-von-Imhof-Forum 2, 85354 Freising-Weihenstephan, Germany.
[11]German Centre for Integrative Biodiversity Research (iDiv) Halle-Jena-Leipzig, Puschstraße 4, 04103 Leipzig, Germany.
[*]corresponding author: sneumann@ipb-halle.de

Abstract

This document describes the preprocessing of a large public untargeted metabolomics dataset using the *xcms* package using its new memory-saving data representation mode. This data representation mode enables analysis of very large data sets (the present consisting of about 4,000 samples) also on conventional computer hardware. Performance and memory usage of different storage modes are subsequently evaluated on a smaller data subset. Finally, details on the internal data and memory handling of *xcms* are presented and properties of different configurations for efficient (and parallel) processing of large scale data discussed.

Note: this vignette is **pre-computed**. See the session info for information on packages used and the date the vignette was rendered. The vignette is completely reproducible and can be run and evaluated locally. The full MS data is available in the MetaboLights repository and is downloaded and cached as part of this vignette. Thus, to run the code locally about 800GB of free disk space is required.

Note: the memory-saving functionality used in this document requires *xcms* version >= 3.8 which available since Bioconductor release 3.22.

Information on computational setup: this vignette was rendered on a Framework notebook with an 13th Gen Intel(R) Core(TM) i7-1370P CPU and 64GB of main memory.

Introduction

By design, *xcms* supports preprocessing of large scale data even with over 10,000 samples/data files. Handling the results of such experiments is however non-trivial and can require a very large amount of main memory. Recent updates to *xcms*, which include full support of the MS data infrastructure provided by the *Spectra* package as well as a new *on-disk* storage mode for preprocessing results, further reduce *xcms*' memory demand and hence enable memory-saving large scale data processing also on regular computer setups. In this document a large public metabolomics data set with about 4,000 data files is analyzed and *xcms*' memory usage and performance tracked. Performance and memory usage for different configurations are compared on a smaller data subset. Finally, details on the internal data and memory handling of *xcms* are presented and properties of different configurations for efficient (and parallel) processing of large scale data are discussed.

Data import

The data analyzed in this document was originally described in this paper. The full data is available in the MetaboLights repository with the accession ID MTBLS93. Detailed description on the study cohort, LC-MS setup and the data acquisition are provided in the original article's supplement. In brief, samples were analyzed in Waters MSe mode, i.e., following an MS1 scan, an all-ion fragmentation was performed and recorded as a MS2 scan. MS1 and MS2 data are for each samples are stored in two separate MS data files in CDF format. MS1 data in files ending with *01.CDF* and the respective MS2 scans in a file with same name, but ending in *02.CDF*. For the present analysis we focus on MS1 data only and thus restrict the import to the MS1-only data files. The *MsIO* R package and Bioconductor's *MsBackendMetaboLights* packages are used to retrieve and cache the MS data directly from the MetaboLights repository.

```
#' Load required libraries
library(MsBackendMetaboLights)
library(MsExperiment)
library(MsIO)
library(xcms)

library(peakRAM) # Track memory usage and processing time
library(pander)  # To render tables

#' Retrieve the MS1 data from the MetaboLights data set
mlp <- MetaboLightsParam(mtblsId = "MTBLS93", filePattern = "01.CDF$")

twins <- readMsObject(MsExperiment(), mlp, keepOntology = FALSE,
                      keepProtocol = FALSE, simplify = FALSE)
twins
#> Object of class MsExperiment
#>  Spectra: MS1 (12082605)
#>  Experiment data: 4063 sample(s)
#>  Sample data links:
```

```
#>   - spectra: 4063 sample(s) to 12082605 element(s).
```

The data set includes in total 12082605 MS spectra for 4063 samples.

The size of the data object in memory:

```
print(object.size(twins), units = "GB")
#> 2.8 Gb
```

Note that this data object contains only the MS metadata (i.e., retention times, MS levels etc), but no MS peaks data (i.e., *m/z* and intensity values). With the default data representation of the *Spectra* package, MS peaks data are only loaded upon demand from the original data files.

Overview of sample and experiment metadata

Various experimental and sample metadata are available for the data set in the imported object's `sampleData()`. These are directly imported from the respective data files in *MetaboLights*. The format of the imported variable names is however not ideal for R-based data processing and we thus rename the most relevant ones below.

```
#' Select variable names with eventually interesting information
scol <- c("Factor Value[Gender]", "Factor Value[Age]",
          "Factor Value[Cluster effect]", "Factor Value[RMSD]",
          "Factor Value[Injection number]", "Factor Value[Spectrum type]",
          "Factor Value[Analysis date]", "Sample Name")
#' Define R-save names for these
names(scol) <- c("sex", "age", "cluster_effect", "rmsd", "injection_number",
                 "spectrum_type", "analysis_date", "sample_name")
#' Rename the variables
colnames(sampleData(twins))[match(scol, colnames(sampleData(twins)))] <-
    names(scol)

table(sampleData(twins)$sex)
#>
#> Female   Male
#>   1747   2316
```

Parallel processing setup

Many functions from *xcms*, in particular the ones requiring heavy calculations, support parallel processing. Processing functions will split the data among these processes and perform the calculations in parallel. While parallel processing can reduce the processing time, it is important to note that it also requires all data that is being processed to be in memory. There should thus always be a balance between the number of parallel processes and the available/required memory needed. See also section *Performance evaluation* below for more information. In our example we use 8 CPUs in parallel.

```
#' Default parallel processing setup.
register(MulticoreParam(8L))
```

Every function from *xcms* supporting parallel processing will now use this default parallel processing setup.

Initial data evaluation

Before preprocessing, we inspect the available LC-MS data and create a base peak chromatogram (BPC). To reduce the processing time, we create this BPC on 200 randomly selected samples.

```r
#' Select 200 randomly selected samples
set.seed(123)
twins_rand <- twins[sample(seq_along(twins), 200)]
```

We next create the BPC. With parameter `chunkSize = 8L` we specify to load and process the MS peaks data of 8 MS data files at a time. The `peakRAM()` function is used to track memory usage and processing time. While the function will process the data of 8 files at a time in parallel, there is no large performance gain for that, because the processing consists of simply returning the maximum intensity per spectrum.

```r
#' Create the BPC for the data subset
p <- peakRAM(
    bpc <- chromatogram(twins_rand, aggregationFun = "max", chunkSize = 8)
)
```

The time and maximal (peak) memory used are:

```r
tbl <- data.frame(
    `Peak RAM [MiB]` = p$Peak_RAM_Used_MiB,
    `Processing time [hours]` = p$Elapsed_Time_sec / 60 / 60,
    check.names = FALSE)
pandoc.table(
    tbl, style = "rmarkdown", split.table = Inf,
    caption = paste0("Peak RAM memory usage and processing time for ",
                     "BPC extraction from 200 random samples."))
```

Table 1: Peak RAM memory usage and processing time for BPC extraction from 200 random samples.

Peak RAM [MiB]	Processing time [hours]
5001	0.08255

The BPC of these 200 random samples is shown below.

```r
plot(bpc, col = "#00000060")
```

Based on the BPC above, we filter the data set to spectra measured between 20 and 900 seconds. Such restriction of the data set also avoids to perform the chromatographic peak detection in the part of the LC where no compounds are expected to elute. We strongly recommend such filtering before starting preprocessing to reduce running time.

```r
twins <- filterSpectra(twins, filterRt, rt = c(20, 900))
```

We next inspect the signal for selected lipids that have been annotated in the original article (described in Figure S2 of the original paper). The retention times and *m/z* values for these compounds were taken from the original publication.

```r
#' Define m/z and retention times for annotated lipids
kc <- data.frame(
    name = c("LysoPC_18:1", "LysoPC_18:2", "MG_18:2"),
    mz = c(522.356, 520.341, 337.269),
    rt = c(382.20, 347.28, 384.00)
)
rownames(kc) <- kc$name
kc$rtmin <- kc$rt - 20
kc$rtmax <- kc$rt + 20
kc$mzmin <- kc$mz - 0.01
```

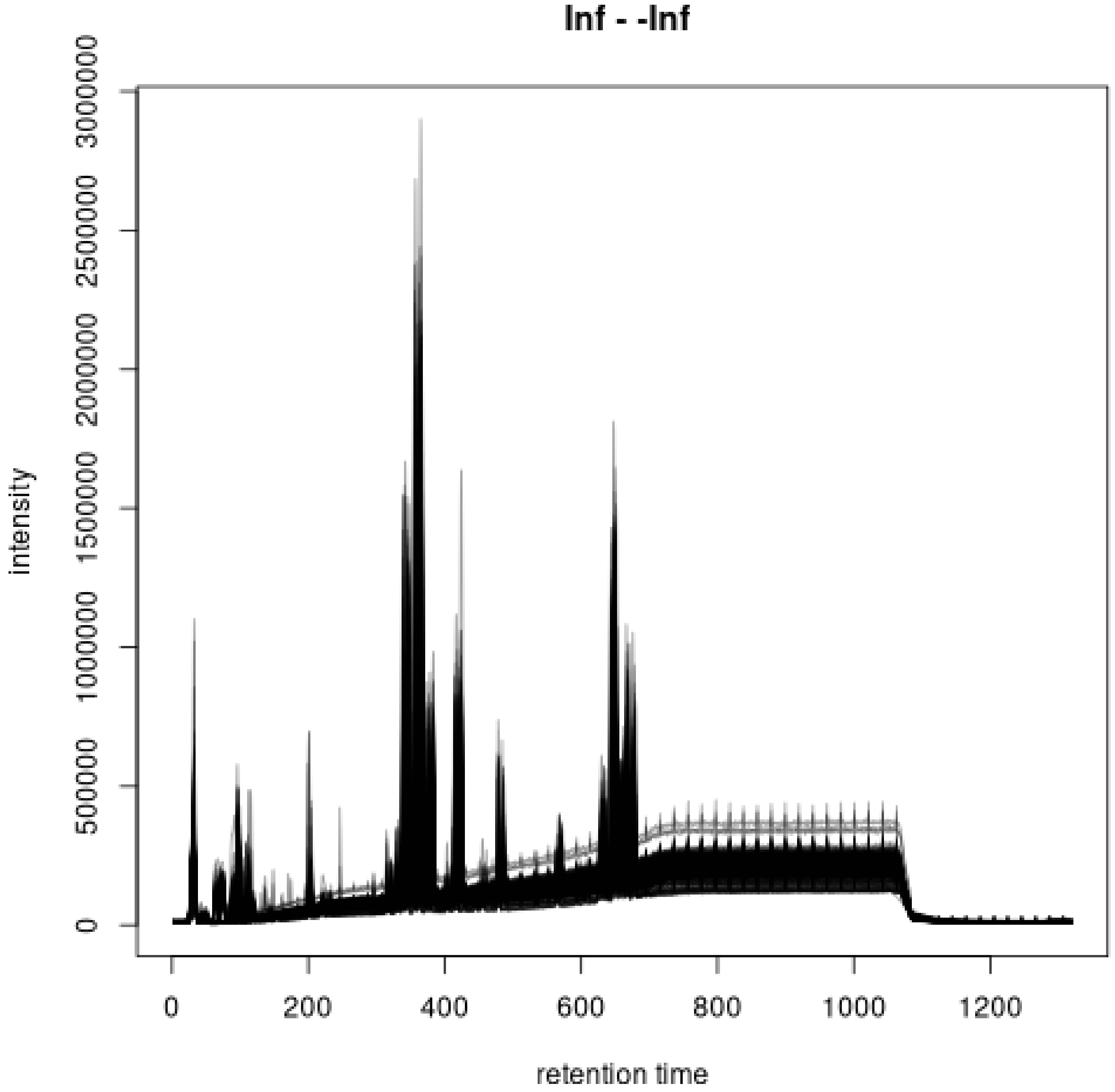

Figure 1: Base peak chromatogram of the full data set.

```r
kc$mzmax <- kc$mz + 0.01

pandoc.table(
    kc, split.table = Inf, style = "rmarkdown",
    caption = "Selected lipids that were annotated in the original article.")
```

Table 2: Selected lipids that were annotated in the original article.

	name	mz	rt	rtmin	rtmax	mzmin	mzmax
**LysoPC__18:1**	LysoPC__18:1	522.4	382.2	362.2	402.2	522.3	522.4
**LysoPC__18:2**	LysoPC__18:2	520.3	347.3	327.3	367.3	520.3	520.4
**MG__18:2**	MG__18:2	337.3	384	364	404	337.3	337.3

We extract and plot the ion chromatograms (EIC) for these 3 compounds from the 200 randomly selected samples.

```r
#' Extract the ion chromatogram for the 3 compounds
eics <- chromatogram(twins_rand, mz = cbind(kc$mzmin, kc$mzmax),
                     rt = cbind(kc$rtmin, kc$rtmax), chunkSize = 8)
#' Plot the EICs
plot(eics, col = "#00000040")
```

Apparent retention time shifts are visible for all 3 compounds. We can next evaluate chromatographic peak detection settings on these example signals.

```r
param <- CentWaveParam(ppm = 25,
                       peakwidth = c(2, 20),
                       snthresh = 0,
                       mzCenterFun = "wMean",
                       integrate = 2)
met_test <- findChromPeaks(eics, param = param)
chromPeaks(met_test[1]) |>
    head()
#>              mz   mzmin   mzmax      rt   rtmin   rtmax      into       intb
#> mzmin 522.356 522.346 522.366 383.178 375.894 388.319 2329316.3 1691671.50
#> mzmin 522.356 522.346 522.366 382.749 375.894 387.890 2109446.7 1535929.04
#> mzmin 522.356 522.346 522.366 382.318 375.034 387.031 2393436.5 1751258.46
#> mzmin 522.356 522.346 522.366 370.321 363.636 375.034  434015.4   74434.92
#> mzmin 522.356 522.346 522.366 382.747 376.321 388.317 2208231.7 1624551.74
#> mzmin 522.356 522.346 522.366 382.318 374.606 387.031 2392099.1 1752668.52
#>            maxo sn row column
#> mzmin 505062.25  5   1      1
#> mzmin 469037.25  5   1      2
#> mzmin 527234.00  5   1      3
#> mzmin  99709.62  0   1      3
#> mzmin 476111.50  5   1      4
#> mzmin 544067.50  5   1      5
plot(met_test)
```

Chromatographic peaks were detected in all cases, but due to the large number of samples investigated it is not easy to evaluate the results properly. We thus create the same plot for only the first 5 samples.

```r
plot(met_test[, 1:5])
```

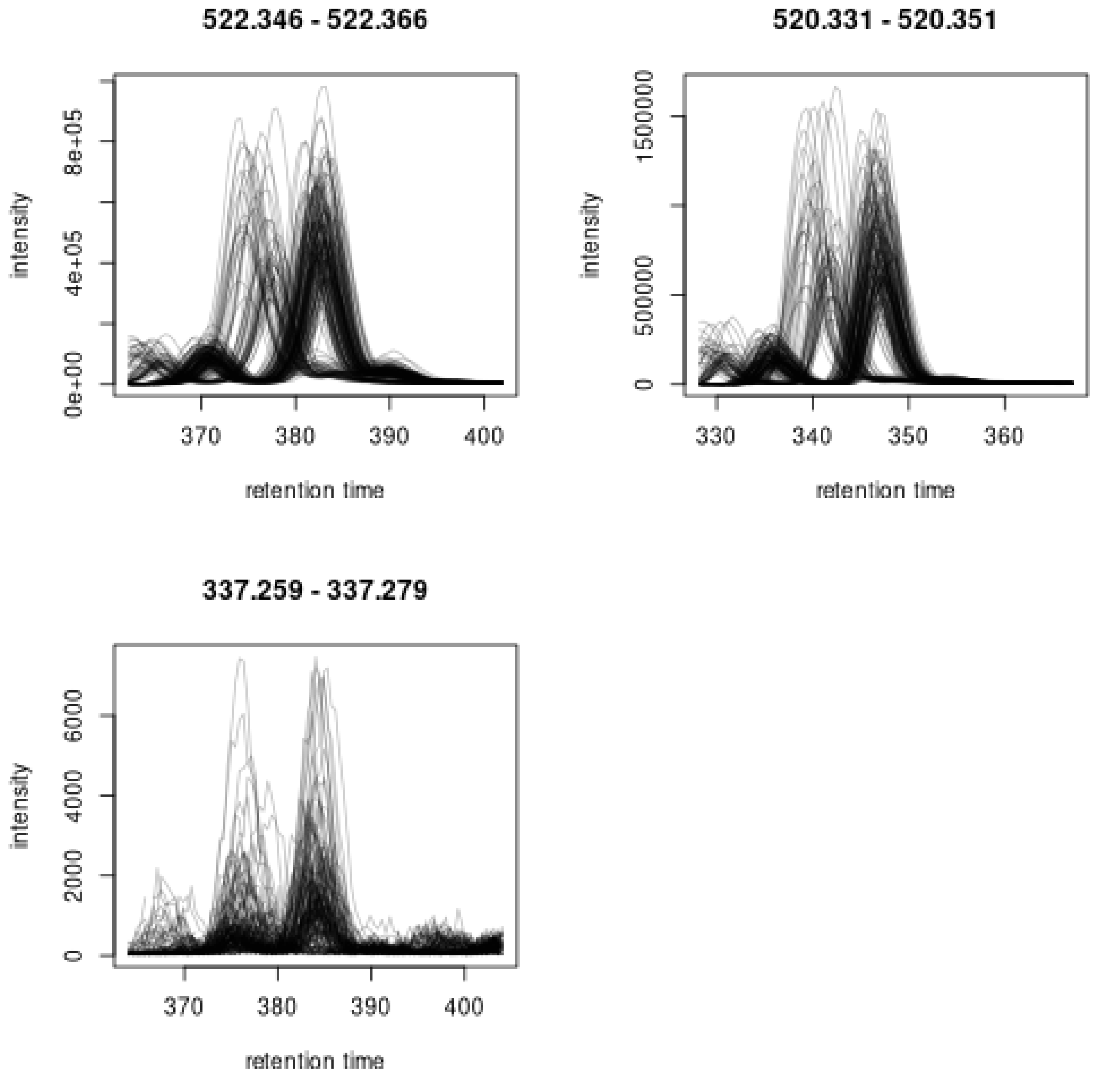

Figure 2: EIC plots for 3 compounds in 200 randomly selected samples.

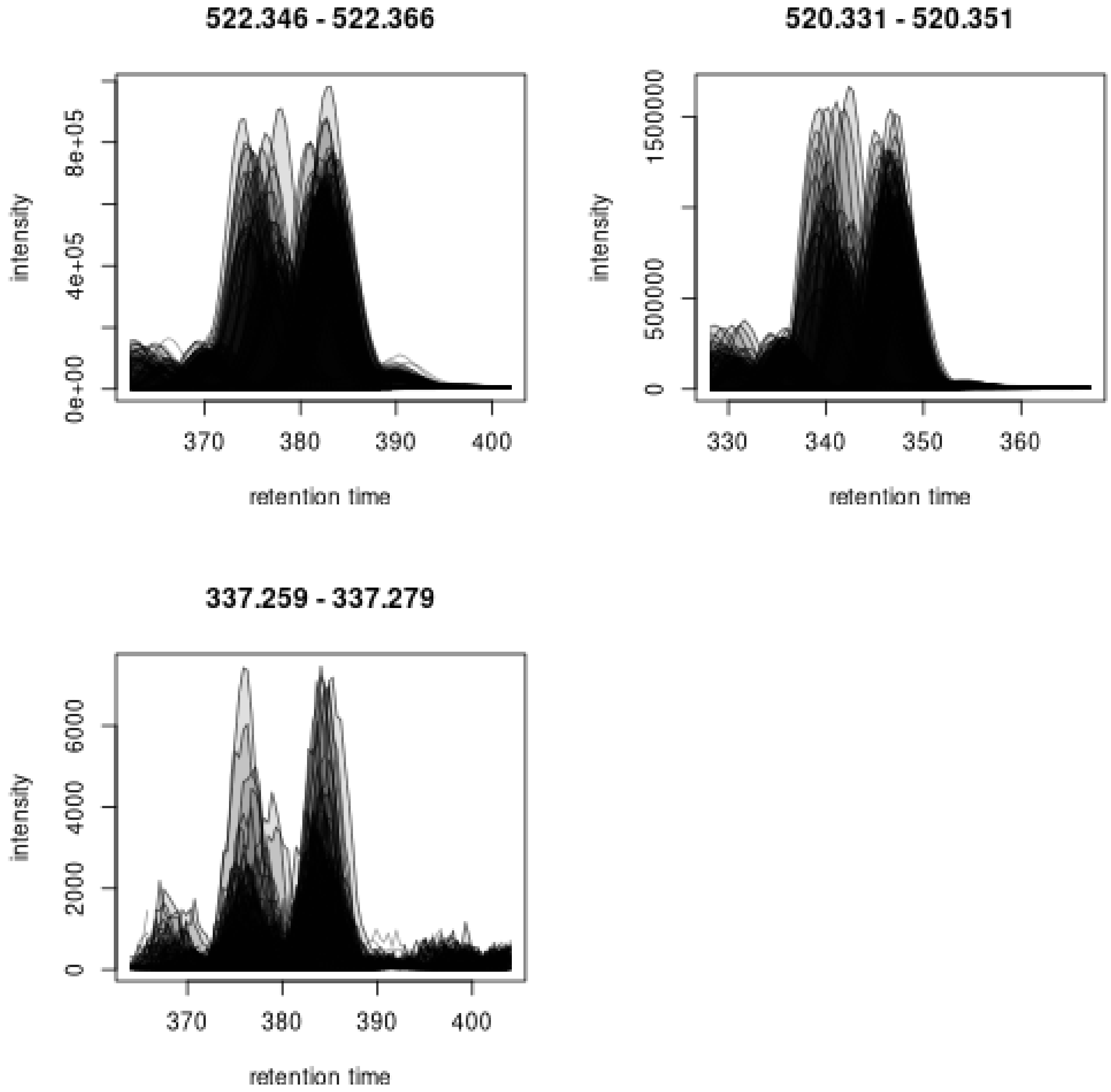

Figure 3: EIC plots for 3 compounds in 200 randomly selected samples with identified chromatographic peaks highlighted (grey color).

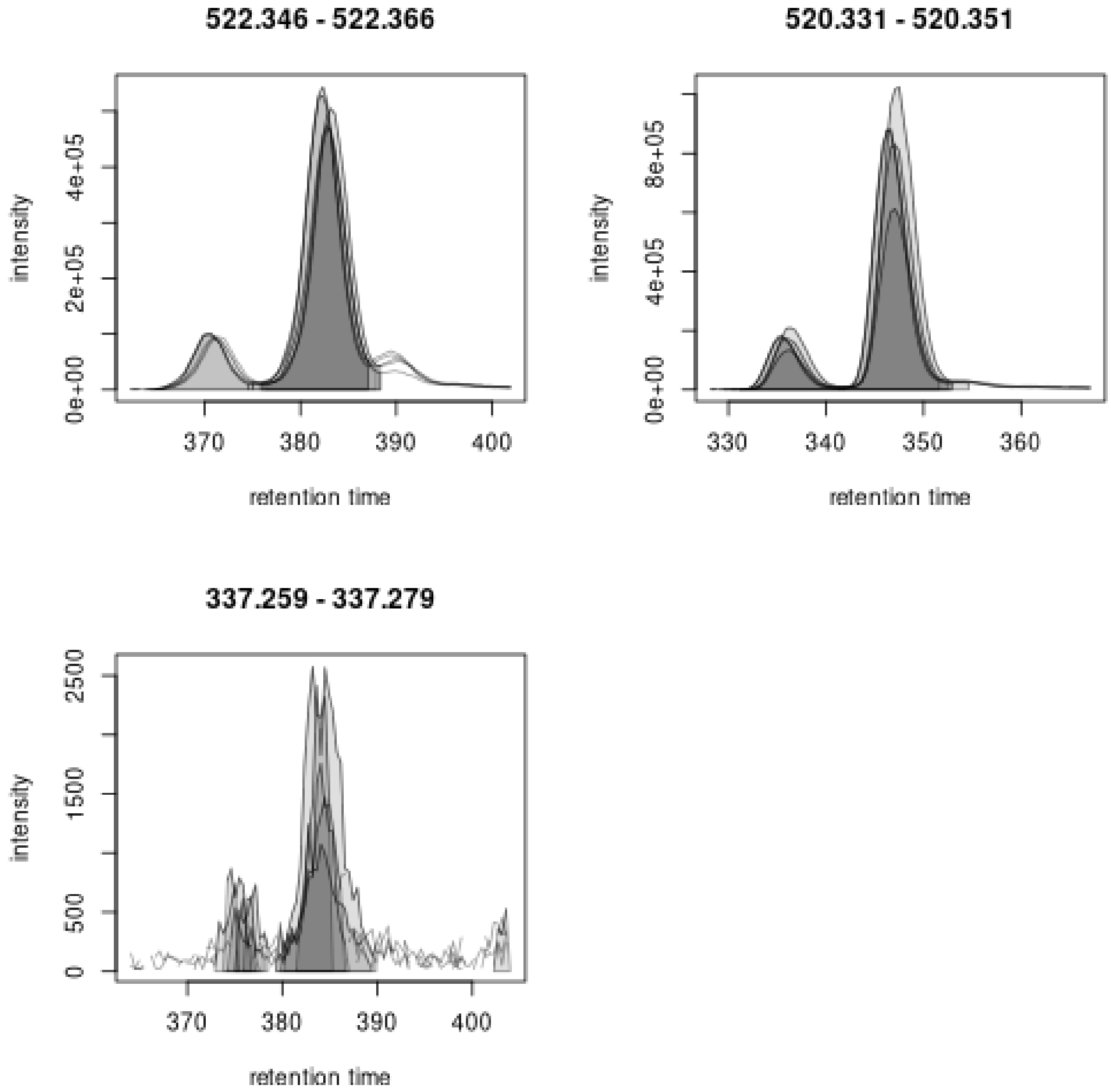

Figure 4: EIC plots for 3 compounds in 5 samples. Detected chromatographic peaks are indicated with a grey color.

The large peak was thus correctly identified. Also the lower abundance peak would be detected if the `snthresh` would be reduced for the peak detection in the EIC signal. Noise estimation is difficult for peak detection in extracted ion signals, as most of the chromatogram contains actual signal from the ion. This is different for the preprocessing on the full data set performed in the next section as much more *real* background signal is present in the full MS data to properly estimate the noise.

Preprocessing

We next perform the preprocessing of the LC-MS data. Settings of the individual processing steps were taken from the original data analysis R script and adapted to the new *xcms* interface.

At first we perform the chromatographic peak detection using the *centWave* method. With the parameter `hdf5File` we define the name (and eventually path) for a file to keep the preprocessing results. Information on identified chromatographic peaks and results from later preprocessing steps will then be stored into this file. The HDF5 file format guarantees efficient storage, and retrieval, of these results or subsets thereof. The memory footprint of this new result object is thus very small which is ideal for the processing of very large data sets, also on conventional computing infrastructure (e.g. laptop). With `chunkSize = 8` we define to load and process the MS data of 8 data files at a time. Peak detection is then performed in parallel on 8 CPUs using our predefined default parallel processing setup.

```
cwp <- CentWaveParam(ppm = 25,
                     peakwidth = c(2, 20),
                     prefilter = c(3, 500),
                     snthresh = 8,
                     mzCenterFun = "wMean",
                     integrate = 2)

if (file.exists("twins.h5")) invisible(file.remove("twins.h5"))
p <- peakRAM(
    twins <- findChromPeaks(twins, param = cwp, chunkSize = 8,
                            hdf5File = "twins.h5")
)
```

Memory usage and time elapsed for this processing step where:

```
tbl <- data.frame(
    `Peak RAM [MiB]` = p$Peak_RAM_Used_MiB,
    `Processing time [min]` = p$Elapsed_Time_sec / 60,
    check.names = FALSE)
pandoc.table(
    tbl, style = "rmarkdown", split.table = Inf,
    caption = paste0("Peak RAM memory usage and processing time for ",
                     "the chromatographic peak detection step on the",
                     " full data."))
```

Table 3: Peak RAM memory usage and processing time for the chromatographic peak detection step on the full data.

Peak RAM [MiB]	Processing time [min]
7054	572

As a result, the `findChromPeaks()` function returned an object of type `XcmsExperimentHdf5`, which, as described above, stored all preprocessing results on-disk in a file in HDF5 format. The size of this result object in memory is thus not much larger than the original object representing the MS data:

```r
print(object.size(twins), units = "GB")
#> 1.8 Gb
```

The identified chromatographic peaks are stored as a numeric matrix. Depending on the size of the experiment and MS data files as well as the used peak detection settings, this matrix can also be very large and use a big part of the main memory, which can significantly slow down subsequent analysis steps. Below we load this data matrix into memory to evaluate its size.

```r
#' Load the chromatographic peak detection results and
#' get its size.
chromPeaks(twins) |>
    object.size() |>
    print(units = "GB")
#> 3.4 Gb
```

For the present data set and the used settings, the size of the chromatographic peak matrix seems manageable also for regular computers. However, having this data object all the time in memory can have negative impact on processing efficiency and in the worst case have R running out of memory along the further analysis.

Below we count the number of chromatographic peaks detected per sample and determine also their total sum. Here we take advantage of the possibility to load only selected columns of the chromatographic peak matrix, which will reduce the memory need for the present calculation. Also, we use `bySample = TRUE` which returns the result as a `list` of chromatographic peak matrices. The length of the list is equal to the number of samples and each element is the chromatographic peak matrix of one sample.

```r
#' Load the "into" column from the chrom peak matrix
pc <- chromPeaks(twins, columns = "into", bySample = TRUE)

#' The distribution of the number of peaks counts
vapply(pc, nrow, integer(1)) |>
    quantile()
#>     0%    25%    50%    75%   100%
#> 3344.0 4919.5 5511.0 6124.0 9167.0
```

Between 5000 and 6000 chromatographic peaks have been detected per sample. The total number of peaks is:

```r
vapply(pc, nrow, integer(1)) |>
    sum()
#> [1] 22568648
```

Next we perform the *peak refinement*. This step helps to reduce common peak detection artifacts, such as duplicated peaks, overlapping peaks or artificially split peaks. Again, we process the data in chunks of 8 data files at a time to keep memory usage manageable for the used computer system.

```r
#' Perform peak refinement
mnpp <- MergeNeighboringPeaksParam(expandRt = 5)

p <- peakRAM(
    twins <- refineChromPeaks(twins, param = mnpp, chunkSize = 8)
)
```

Memory usage and time elapsed for this processing step where:

```r
tbl <- data.frame(
    `Peak RAM [MiB]` = p$Peak_RAM_Used_MiB,
    `Processing time [hours]` = p$Elapsed_Time_sec / 60 / 60,
    check.names = FALSE)
pandoc.table(
```

```
    tbl, style = "rmarkdown", split.table = Inf,
    caption = paste0("Peak RAM memory usage and processing time for ",
                     "the chromatographic peak refinement."))
```

Table 4: Peak RAM memory usage and processing time for the chromatographic peak refinement.

Peak RAM [MiB]	Processing time [hours]
5870	3.305

For retention time alignment we will use the *peak groups* method, that adjusts retention time shifts between samples based on the retention times of common compounds present in most samples (the so called *anchor peaks*). We thus run next an initial correspondence analysis (with relaxed settings for retention time differences) in order to define these.

```
#' Define settings for an initial correspondence analysis:
#' require anchor peaks to be present in 70% of samples, but use a
#' larger bw to allow rt shifts
pdp <- PeakDensityParam(sampleGroups = rep(1L, length(twins)),
                        minFraction = 0.7,
                        binSize = 0.01,
                        ppm = 10,
                        bw = 3.5)

p <- peakRAM(
    twins <- groupChromPeaks(twins, param = pdp)
)
```

```
tbl <- data.frame(
    `Peak RAM [MiB]` = p$Peak_RAM_Used_MiB,
    `Processing time [min]` = p$Elapsed_Time_sec / 60,
    check.names = FALSE)
pandoc.table(
    tbl, style = "rmarkdown", split.table = Inf,
    caption = paste0("Peak RAM memory usage and processing time for ",
                     "a correspondence analysis."))
```

Table 5: Peak RAM memory usage and processing time for a correspondence analysis.

Peak RAM [MiB]	Processing time [min]
5870	1.428

We can next perform the retention time alignment using the *peak groups* method.

```
pgp <- PeakGroupsParam(minFraction = 0.8, extraPeaks = 100,
                       span = 0.5)
#' Perform the alignment
p <- peakRAM(
    twins <- adjustRtime(twins, param = pgp)
)
```

```r
tbl <- data.frame(
    `Peak RAM [MiB]` = p$Peak_RAM_Used_MiB,
    `Processing time [min]` = p$Elapsed_Time_sec / 60,
    check.names = FALSE)
pandoc.table(
    tbl, style = "rmarkdown", split.table = Inf,
    caption = paste0("Peak RAM memory usage and processing time for ",
                     "the retention time alignment analysis."))
```

Table 6: Peak RAM memory usage and processing time for the retention time alignment analysis.

Peak RAM [MiB]	Processing time [min]
2820	1.617

We evaluate the performance of the alignment on EICs for the 3 known compounds in 200 random samples.

```r
#' Select 200 random samples and extract ion chromatograms for the 3
#' annotated lipids. We reuse the same random seed to choose the same
#' random samples selected before. Note that we use `keepAdjustedRtime = TRUE`
#' to avoid the retention times being restored to the original values.
set.seed(123)
twins_rand <- twins[sample(seq_along(twins), 200), keepAdjustedRtime = TRUE]
```

```r
#' Extract the ion chromatogram for the 3 compounds
eics <- chromatogram(twins_rand, mz = cbind(kc$mzmin, kc$mzmax),
                     rt = cbind(kc$rtmin, kc$rtmax), chunkSize = 8)
#' Plot the EICs
plot(eics, col = "#00000020", peakBg = NA)
```

The EICs are nicely aligned (see Figure 5).

We perform now the final correspondence analysis to match aligned chromatographic peaks across samples and define the *LC-MS features*. We reduce `minFraction` parameter to also define features if chromatographic peaks were only present in 30% of the samples and use a more stringent `bw` parameter.

```r
pdp <- PeakDensityParam(sampleGroups = rep(1L, length(twins)),
                        minFraction = 0.3,
                        binSize = 0.01,
                        ppm = 10,
                        bw = 1.5)

p <- peakRAM(
    twins <- groupChromPeaks(twins, param = pdp)
)
```

```r
tbl <- data.frame(
    `Peak RAM [MiB]` = p$Peak_RAM_Used_MiB,
    `Processing time [min]` = p$Elapsed_Time_sec / 60,
    check.names = FALSE)
pandoc.table(
    tbl, style = "rmarkdown", split.table = Inf,
    caption = paste0("Peak RAM memory usage and processing time for ",
                     "the final correspondence analysis."))
```

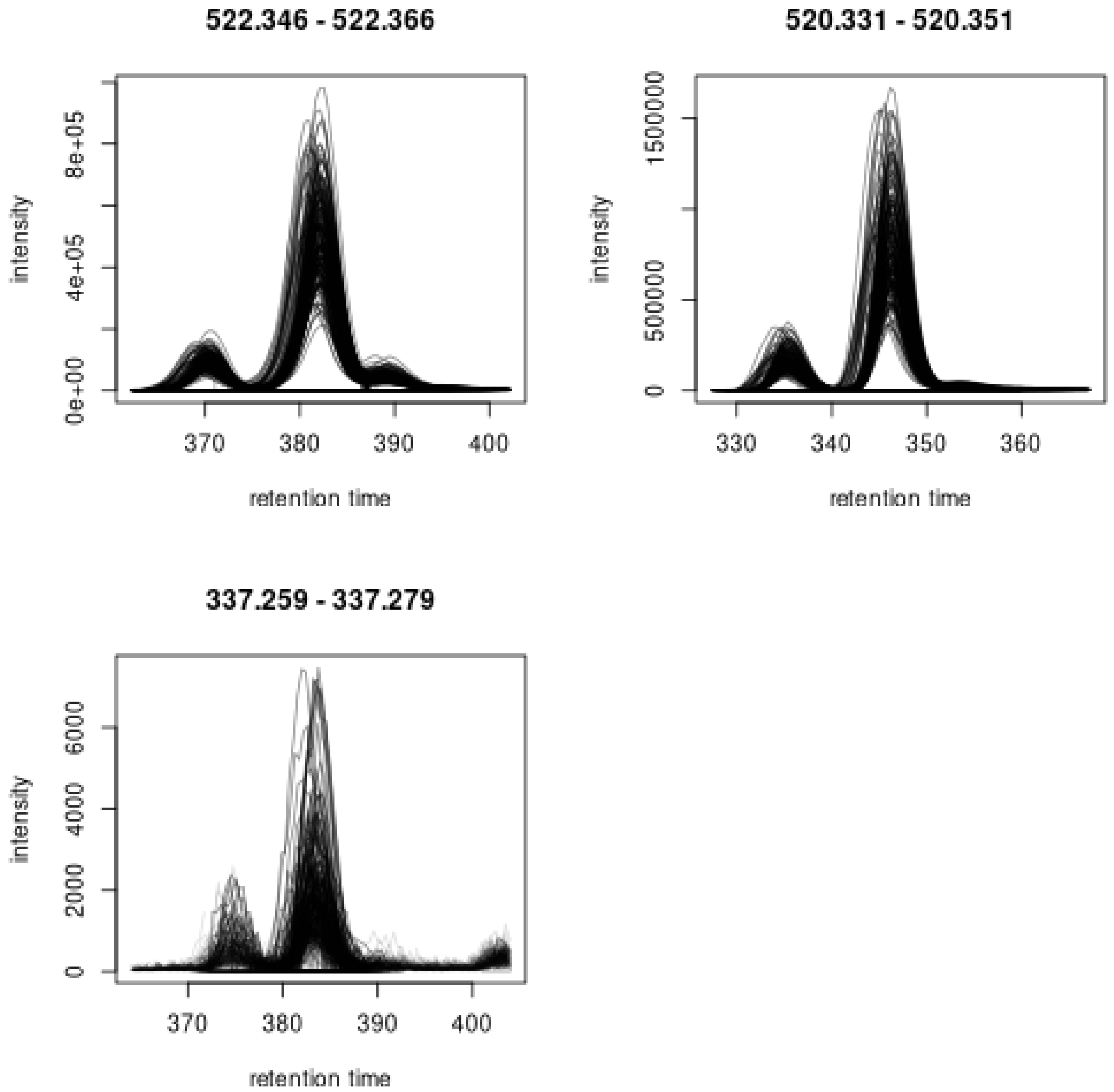

Figure 5: Example EICs after alignment.

Table 7: Peak RAM memory usage and processing time for the final correspondence analysis.

Peak RAM [MiB]	Processing time [min]
5798	2.425

We also evaluate whether the correspondence could correctly define features for the 3 selected lipids.

```
set.seed(123)
twins_rand <- twins[sample(seq_along(twins), 200),
                    keepAdjustedRtime = TRUE, keepFeatures = TRUE]
eics <- chromatogram(twins_rand, mz = cbind(kc$mzmin, kc$mzmax),
                     rt = cbind(kc$rtmin, kc$rtmax), chunkSize = 8)
```

Correspondence was able to correctly assign the chromatographic peaks left of the center peaks into a separate feature.

```
plotChromPeakDensity(eics[1])
```

Also for the second example, the chromatographic peaks were assigned to two separate features.

```
plotChromPeakDensity(eics[2])
```

Finally, also for the last example chromatographic peaks were correctly assigned to features.

```
plotChromPeakDensity(eics[3])
```

Below we extract the feature abundances for all features in all samples and count the total number of defined features. The `featureValues()` function collects and extracts this information from the associated HDF5 file used to store the correspondence results.

```
#' Extract the feature abundances for all features in all samples
fvals <- featureValues(twins, value = "into", method = "sum")
nrow(fvals)
#> [1] 4981
```

The total number of missing values in this data matrix is:

```
sum(is.na(fvals))
#> [1] 6690736
```

As a last step we therefore perform the gap-filling to *rescue* abundances for features for which in some samples no chromatographic peak was detected (and for which hence a missing value is reported). Here we can again benefit from chunk-wise parallel processing.

```
#' Perform the gap filling
cpap <- ChromPeakAreaParam()

p <- peakRAM(
    twins <- fillChromPeaks(twins, param = cpap, chunkSize = 8)
)
```

```
tbl <- data.frame(
    `Peak RAM [MiB]` = p$Peak_RAM_Used_MiB,
    `Processing time [hours]` = p$Elapsed_Time_sec / 60 / 60,
    check.names = FALSE)
pandoc.table(
    tbl, style = "rmarkdown", split.table = Inf,
```

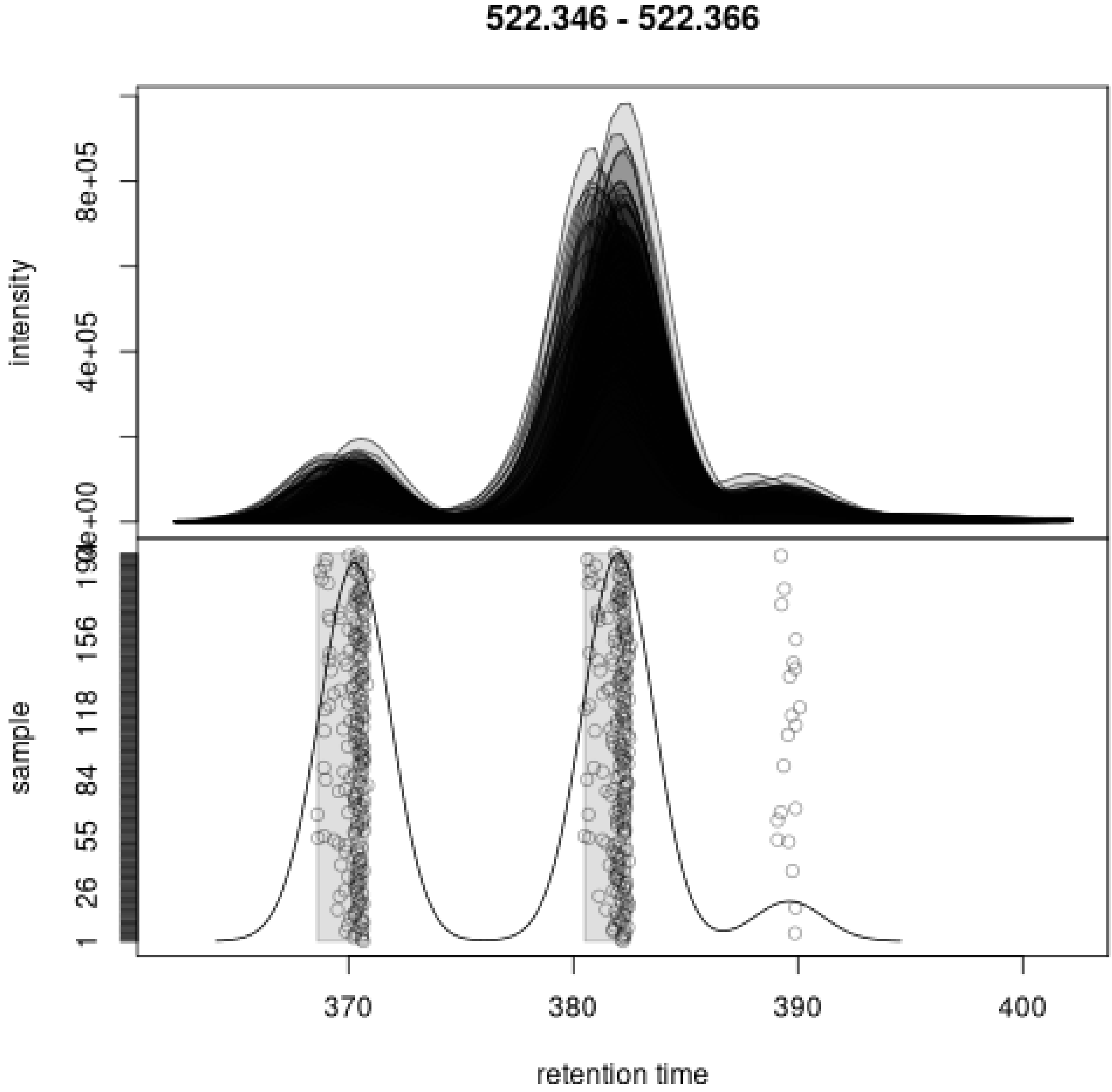

Figure 6: Evaluation of correspondence settings on compound 1.

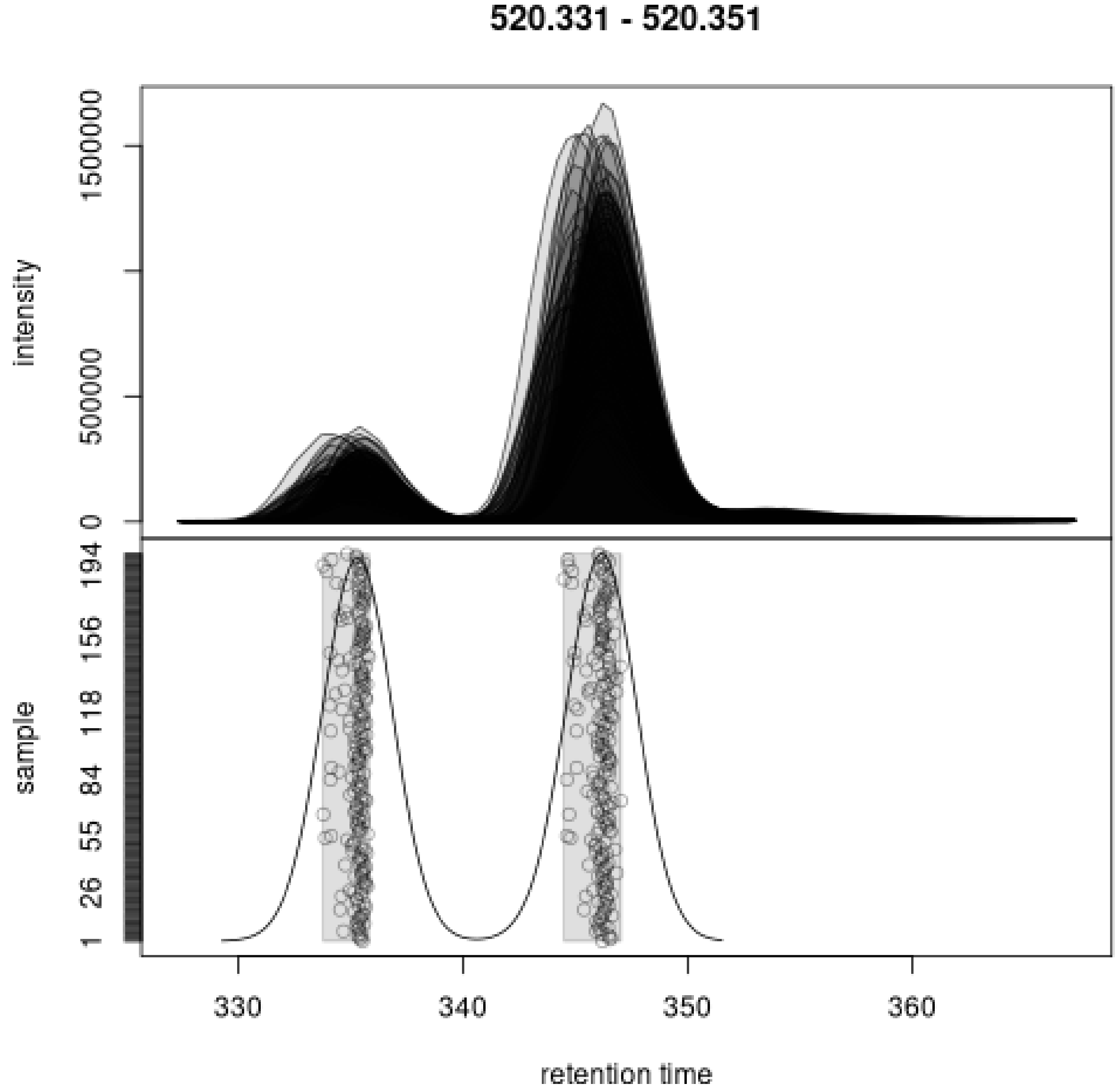

Figure 7: Evaluation of correspondence settings on compound 2.

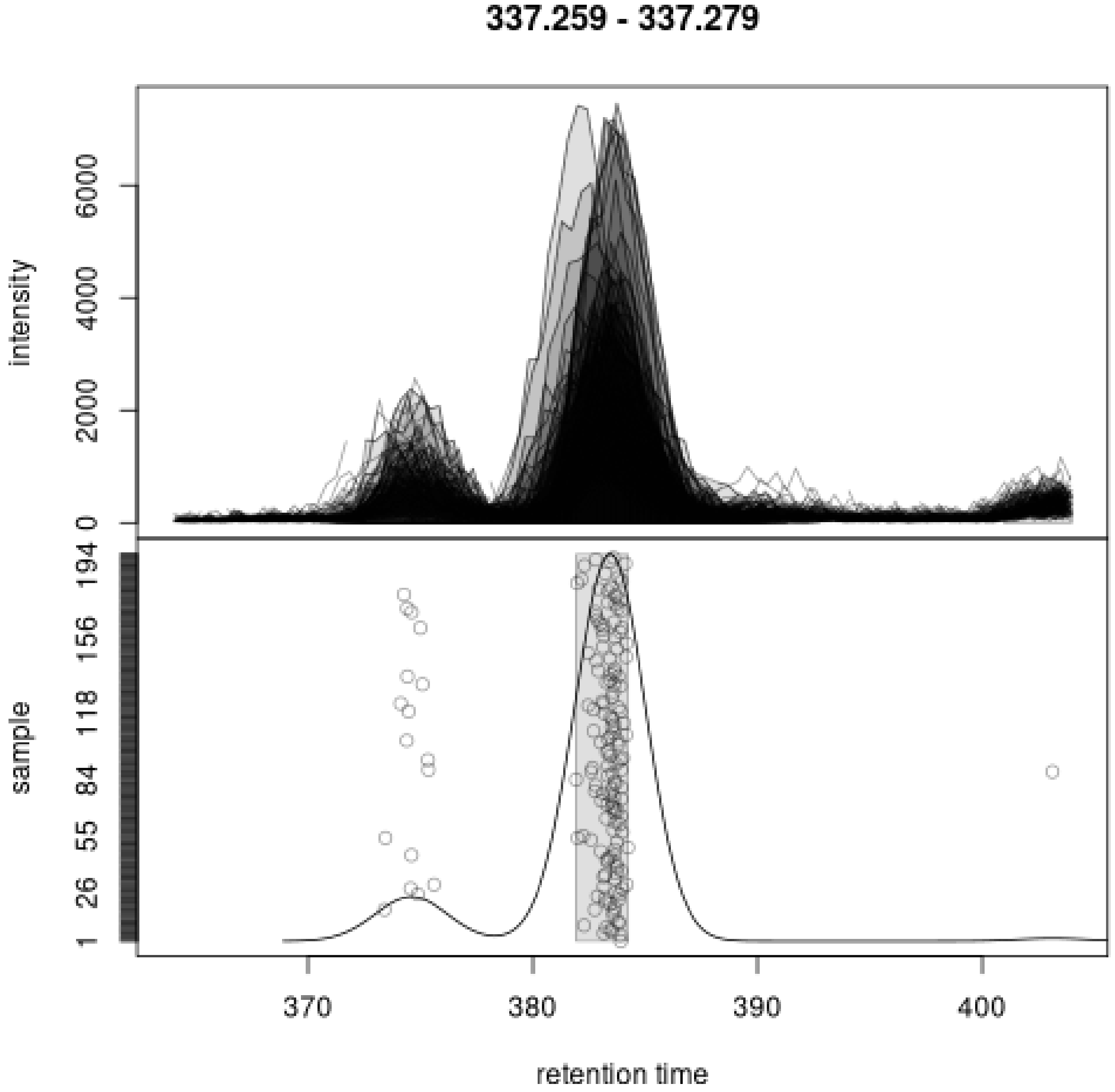

Figure 8: Evaluation of correspondence settings on compound 3.

```
    caption = paste0("Peak RAM memory usage and processing time for ",
                     "gap filling."))
```

Table 8: Peak RAM memory usage and processing time for gap filling.

Peak RAM [MiB]	Processing time [hours]
5645	3.201

The number of missing values after gap filling:

```
sum(is.na(featureValues(twins, value = "into", method = "sum")))
#> [1] 61443
```

Since all preprocessing results are stored in an HDF5 file and hence on-disk, the size of the final results object is not larger than the size of the initial data object representing the MS data of the experiment. Note that this size could be further reduced (and eventually overall performance improved) by storing the MS data in a SQL database and representing/interfacing it with a `Spectra` backend from the *MsBackendSql* package (see the last section in this vignette for more information).

```
print(object.size(twins), unit = "GB")
#> 1.9 Gb
```

The results from the preprocessing could now be converted to a `SummarizedExperiment` object using the `quantify()` function to continue the analysis e.g. by performing further data exploration, normalization or statistical data analysis.

Performance evaluation

In this section we evaluate performance and memory requirements of *xcms* for data preprocessing. R's copy-on-change strategy can be a bottleneck, in particular for very large data sets as data objects (and hence preprocessing results) are copied temporarily during a data analysis. We compare memory usage for the default `XcmsExperiment` object as well as the new `XcmsExperimentHdf5` result object and evaluate scalability of the preprocessing by distributing the load to separate CPUs. In contract to the `XcmsExperiment` object, the `XcmsExperimentHdf5` object keeps all preprocessing results *on-disk* in an HDF5 file reducing thus the memory footprint. Below we create a subset of the data consisting of 100 randomly selected files.

```
set.seed(123)

tsub <- as(twins, "MsExperiment")[sample(seq_along(twins), 100)]
```

We perform chromatographic peak detection on this subset for different configurations:

- Peak detection using the default `XcmsExperiment` result object.
- Peak detection using the `XcmsExperimentHdf5` result object.
- Peak detection using 1, 2, 4 and 8 CPUs for both result objects.

We use a `chunkSize = 8` for all these setups, which will load the MS data for 8 data files into memory. The time and memory usage to access this data is:

```
library(peakRAM)

peakRAM(tmp <- peaksData(spectra(tsub[1:8])))
#>                           Function_Call Elapsed_Time_sec Total_RAM_Used_MiB
#> 1 tmp<-peaksData(spectra(tsub[1:8]))             6.45             1922.9
```

```
#>   Peak_RAM_Used_MiB
#> 1            3865.1
```

And the memory used of the resulting MS data:

```
print(object.size(tmp), units = "GB")
#> 1.9 Gb
rm(tmp)
```

We next execute the performance tests. To use the new `XcmsExperimentHdf5`, we need to specify the path and file name of the HDF5 file where the results should be stored to.

```
#' Settings
cwp <- CentWaveParam(ppm = 25,
                     peakwidth = c(2, 20),
                     prefilter = c(3, 500),
                     snthresh = 8,
                     mzCenterFun = "wMean",
                     integrate = 2)

#' Eventually remove the result HDF5 object if present
if (file.exists("tres.h5")) invisible(file.remove("tres.h5"))

#' Perform the comparison
p <- peakRAM(
    tres <- findChromPeaks(tsub, param = cwp, chunkSize = 8,
                           BPPARAM = SerialParam()),
    findChromPeaks(tsub, param = cwp, chunkSize = 8,
                   BPPARAM = MulticoreParam(2)),
    findChromPeaks(tsub, param = cwp, chunkSize = 8,
                   BPPARAM = MulticoreParam(4)),
    findChromPeaks(tsub, param = cwp, chunkSize = 8,
                   BPPARAM = MulticoreParam(8)),
    tres_h5 <- findChromPeaks(tsub, param = cwp, chunkSize = 8,
                              BPPARAM = SerialParam(), hdf5File = "tres.h5"),
    findChromPeaks(tsub, param = cwp, chunkSize = 8,
                   BPPARAM = MulticoreParam(2), hdf5File = tempfile()),
    findChromPeaks(tsub, param = cwp, chunkSize = 8,
                   BPPARAM = MulticoreParam(4), hdf5File = tempfile()),
    findChromPeaks(tsub, param = cwp, chunkSize = 8,
                   BPPARAM = MulticoreParam(8), hdf5File = tempfile())
)
```

peakRAM can not correctly monitor the memory usage for multi-core processing, thus we evaluate memory usage only for the *serial* processing setup.

Table 9: Peak RAM memory usage and processing time for chromatographic peak detection using different number of CPUs. Data was loaded and processed in chunks of 8 data files.

Result object	CPUs	Peak RAM [MiB]	Processing time [min]
XcmsExperiment	1	5984	38.34
XcmsExperiment	2	5920	24.74
XcmsExperiment	4	5920	18.21
XcmsExperiment	8	5920	16.18

Result object	CPUs	Peak RAM [MiB]	Processing time [min]
XcmsExperimentHdf5	1	5920	38.39
XcmsExperimentHdf5	2	5920	24.91
XcmsExperimentHdf5	4	5920	18.25
XcmsExperimentHdf5	8	5920	16.6

The memory demand is dependent on the `chunkSize` parameter, i.e., the number of files (samples) from which the MS data is loaded and processed at a time. The peak RAM usage for `chunkSize = 8` is, for the present data set, about twice as large as the size of the respective MS data in memory. The processing time is reduced with an increase in the number of CPUs used, but this relationship is not linear. This is most likely due to the overhead in data splitting and distribution to the individual calculation nodes as well as the result collection and their merging/export.

```
plot(tbl[1:4, "CPUs"], tbl[1:4, "Processing time [min]"],
     type = "b", col = "#ff000080", xlab = "CPUs", ylab = "Time [min]")
points(tbl[5:8, "CPUs"], tbl[5:8, "Processing time [min]"],
       type = "b", col = "#0000ff80")
grid()
legend("topright", col = c("#ff000080", "#0000ff80"), pch = 1,
       legend = c("Memory", "HDF5 file"))
```

The largest performance gain is between serial processing and parallel processing with two CPUs. Importantly, the performance of keeping the results in memory (for the `XcmsExperiment` result object) or storing them to a HDF5 file (for the `XcmsExperimentHdf5` file) is comparable.

We next evaluate the performance of the peak refinement step for the different setups re-using the parameter object from the peak refinement on the full data. Processing an `XcmsExperimentHdf5` object will overwrite the results in the associated HDF5 files. To perform the peak refinement on the same initial data we thus need to create copies of the original HDF5 file for each configuration.

```
#' Settings
mnpp <- MergeNeighboringPeaksParam(expandRt = 5)

#' Create copies of the original peak detection results
t1 <- tres_h5
tf <- tempfile()
file.copy(tres_h5@hdf5_file, tf)
#> [1] TRUE
t1@hdf5_file <- tf

t2 <- tres_h5
tf <- tempfile()
file.copy(tres_h5@hdf5_file, tf)
#> [1] TRUE
t2@hdf5_file <- tf

t4 <- tres_h5
tf <- tempfile()
file.copy(tres_h5@hdf5_file, tf)
#> [1] TRUE
t4@hdf5_file <- tf

t8 <- tres_h5
tf <- tempfile()
```

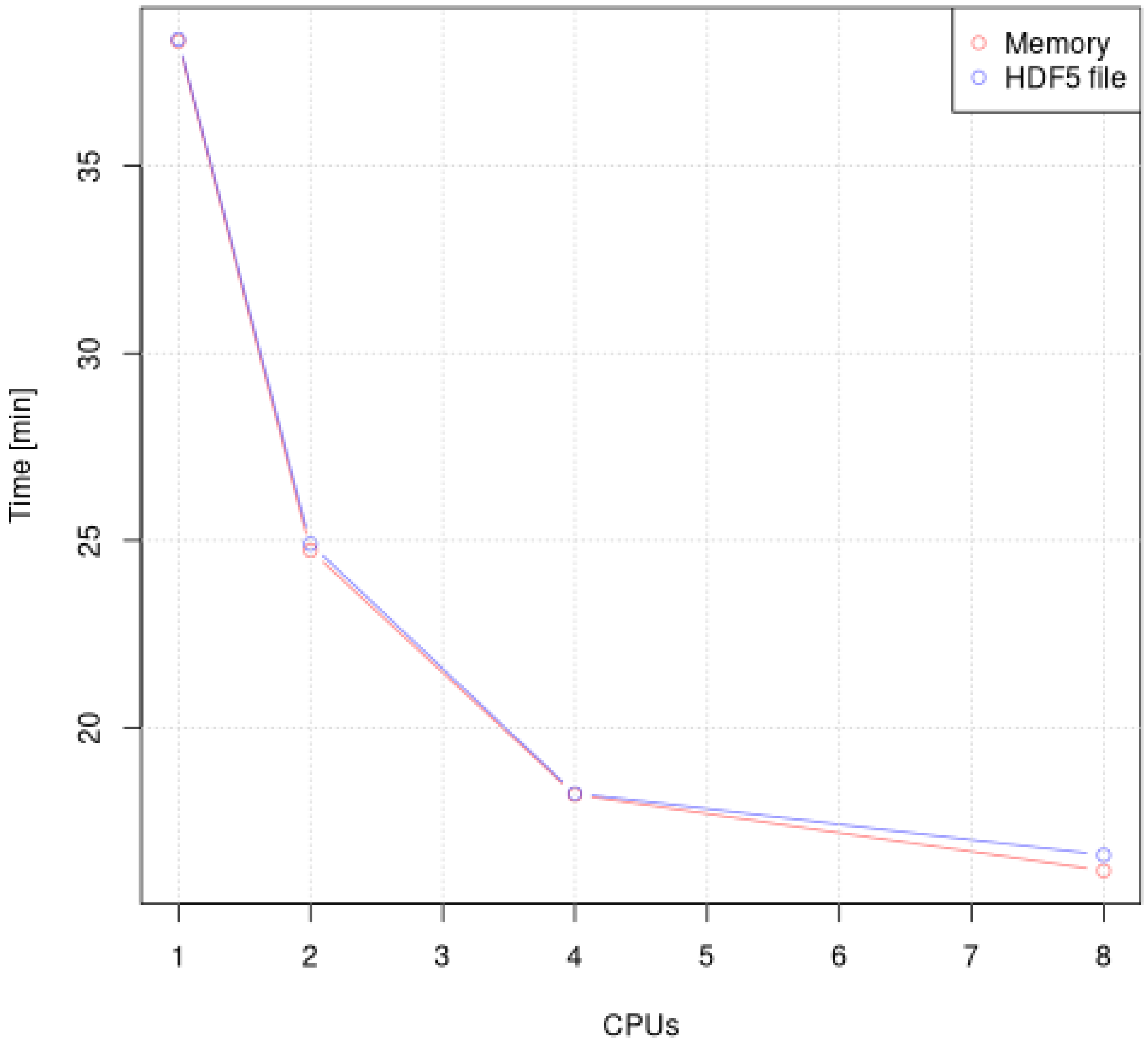

Figure 9: Comparison of computation time depending on the number of CPUs employed for the 2 different xcms result storage modes.

```r
file.copy(tres_h5@hdf5_file, tf)
#> [1] TRUE
t8@hdf5_file <- tf

#' Run peak refinement with different configurations
p <- peakRAM(
    tres_2 <- refineChromPeaks(tres, param = mnpp, chunkSize = 8,
                               BPPARAM = SerialParam()),
    refineChromPeaks(tres, param = mnpp, chunkSize = 8,
                     BPPARAM = MulticoreParam(2)),
    refineChromPeaks(tres, param = mnpp, chunkSize = 8,
                     BPPARAM = MulticoreParam(4)),
    refineChromPeaks(tres, param = mnpp, chunkSize = 8,
                     BPPARAM = MulticoreParam(8)),
    t1 <- refineChromPeaks(t1, param = mnpp, chunkSize = 8,
                           BPPARAM = SerialParam()),
    t2 <- refineChromPeaks(t2, param = mnpp, chunkSize = 8,
                           BPPARAM = MulticoreParam(2)),
    t4 <- refineChromPeaks(t4, param = mnpp, chunkSize = 8,
                           BPPARAM = MulticoreParam(4)),
    t8 <- refineChromPeaks(t8, param = mnpp, chunkSize = 8,
                           BPPARAM = MulticoreParam(8))
)
```

Table 10: Peak RAM memory usage and processing time for chromatographic peak refinement using different number of CPUs. Data was loaded and processed in chunks of 8 data files.

Result object	CPUs	Peak RAM [MiB]	Processing time [min]
XcmsExperiment	1	5920	6.712
XcmsExperiment	2	5863	5.611
XcmsExperiment	4	5863	5.007
XcmsExperiment	8	5864	5.673
XcmsExperimentHdf5	1	5864	6.979
XcmsExperimentHdf5	2	5859	5.752
XcmsExperimentHdf5	4	5859	5.19
XcmsExperimentHdf5	8	5859	7.397

The maximum memory usage is the same for all settings and is again depending on the number of data files from which raw data is imported. The processing time is not reduced considerably by the number of CPUs. This however depends also on the data set and the number of candidate peaks for merging. Processing times are also similar between the in-memory and on-disk results of the `XcmsExperiment` and `XcmsExperimentHdf5` objects.

Next we evaluate the performance of a correspondence analysis with the *peak density* method. This method uses only the `chromPeaks()` matrix for the analysis and does thus not support the `chunkSize` parameter nor does it support parallel processing. We thus only compare the performance of in-memory and on-disk results below.

```r
#' Settings
pdp <- PeakDensityParam(sampleGroups = rep(1, length(tsub)),
                        minFraction = 0.3,
                        binSize = 0.01, ppm = 10,
```

```r
                        bw = 3)

#' Evaluate performance of the correspondence analysis
p <- peakRAM(
    tres_2 <- groupChromPeaks(tres_2, param = pdp),
    t1 <- groupChromPeaks(t1, param = pdp)
)
```

Table 11: Peak RAM memory usage and processing time for correspondence analysis.

Result object	CPUs	Peak RAM [MiB]	Processing time [sec]
XcmsExperiment	1	4166	27.49
XcmsExperimentHdf5	1	3970	27.44

The performance and memory demand is similar for the two result objects.

Next we evaluate the performance of the retention time alignment step using the *peak groups* approach. This method relies on the peak detection and correspondence results and does not support parallel processing.

```r
#' Settings
pgp <- PeakGroupsParam(minFraction = 0.9, extraPeaks = 1000,
                       span = 0.5)

#' Evaluate performance of the correspondence analysis
p <- peakRAM(
    tres_2 <- adjustRtime(tres_2, param = pgp),
    t1 <- adjustRtime(t1, param = pgp)
)
```

```r
tbl <- data.frame(
    `Result object` = rep(c("XcmsExperiment", "XcmsExperimentHdf5")),
    `CPUs` = c(1, 1),
    `Peak RAM [MiB]` = p$Peak_RAM_Used_MiB,
    `Processing time [sec]` = p$Elapsed_Time_sec,
    check.names = FALSE)
pandoc.table(
    tbl, style = "rmarkdown", split.table = Inf,
    caption = paste0("Peak RAM memory usage and processing time for ",
                     "retention time alignment analysis."))
```

Table 12: Peak RAM memory usage and processing time for retention time alignment analysis.

Result object	CPUs	Peak RAM [MiB]	Processing time [sec]
XcmsExperiment	1	4166	27.49
XcmsExperimentHdf5	1	3970	27.44

Processing time is very similar for both result objects, but the memory requirement is much lower for the `XcmsExperimentHdf5` object.

Finally we evaluate the performance of the gap-filling step. This method requires access to the full MS data and can thus again be configured with the `chunkSize` parameter and different parallel processing setups.

```r
#' Settings
cpap <- ChromPeakAreaParam()

p <- peakRAM(
    tres_2 <- fillChromPeaks(tres_2, param = cpap, chunkSize = 8,
                             BPPARAM = SerialParam()),
    fillChromPeaks(tres_2, param = cpap, chunkSize = 8,
                   BPPARAM = MulticoreParam(2)),
    fillChromPeaks(tres_2, param = cpap, chunkSize = 8,
                   BPPARAM = MulticoreParam(4)),
    fillChromPeaks(tres_2, param = cpap, chunkSize = 8,
                   BPPARAM = MulticoreParam(8)),
    t1 <- fillChromPeaks(t1, param = cpap, chunkSize = 8,
                         BPPARAM = SerialParam()),
    t2 <- fillChromPeaks(t2, param = cpap, chunkSize = 8,
                         BPPARAM = MulticoreParam(2)),
    t4 <- fillChromPeaks(t4, param = cpap, chunkSize = 8,
                         BPPARAM = MulticoreParam(4)),
    t8 <- fillChromPeaks(t8, param = cpap, chunkSize = 8,
                         BPPARAM = MulticoreParam(8))
)
```

Table 13: Peak RAM memory usage and processing time for gap filling using different number of CPUs. Data was loaded and processed in chunks of 8 data files.

Result object	CPUs	Peak RAM [MiB]	Processing time [min]
XcmsExperiment	1	5859	5.942
XcmsExperiment	2	5834	2.839
XcmsExperiment	4	5834	2.839
XcmsExperiment	8	5834	2.952
XcmsExperimentHdf5	1	5834	6.127
XcmsExperimentHdf5	2	5834	5.309
XcmsExperimentHdf5	4	5834	4.847
XcmsExperimentHdf5	8	5834	5.395

Details of the software structure and data flow

To reduce memory demand, most processing steps are applied to *chunks* of the data at a time. Thus, only the MS data for the currently processed chunks are realized in memory. By using the *Spectra* package to represent and provide the MS data, *xcms* now also benefits from dedicated `Spectra` data *backends* that e.g. retrieve the MS data on-the-fly from the original MS data files (`MsBackendMzR`) or from an SQL database (using backends from the `r Biocpkg("MsBackendSql")` package). These alternative data representations are seamlessly integrated with *xcms* and increase flexibility while reducing memory demand.

The `XcmsExperiment` result object keeps, similar to the older result objects from *xcms*, all preprocessing results in memory, the chromatographic peak detection results (`chromPeaks()` matrix) in a large numeric matrix and the correspondence results (`featureDefinitions()`) in a data frame. Accessing these data is thus fast, but, depending on the size of the experiment, they can also be very large eventually blocking a large part of the system's main memory. Further, for some additional analysis steps, these tables might need to be further processed, e.g. split by sample, which can result in (at least temporary) additional copies of the data in memory (which can be further complicated by R's copy-on-change strategy). Memory usage can thus, unexpectedly to the user, exceed the available system memory.

The new XcmsExperimentHdf5 was designed to address this issue by storing all preprocessing results in a HDF5 file on disk, keeping thus a very lean memory footprint. Thus, the above described chunk-wise processing of *xcms* will also only load preprocessing results from the currently processed chunk into memory. Importing preprocessing results from the HDF5 file comes with a little overhead, but the functions have been optimized for import of subsets of data at a time.

Summary and guidance

- Use the parameter chunkSize to specify the number of samples/files that should be processed at a time. Ensure to set this value according to the system's main memory. The MS data of samples from one chunk should not exceed the still available (!) system memory.
- Parallel processing helps reduce processing time, but does not scale linearly, since parallel processing involves also distribution of data to, and collection of results from, the individual processing nodes.
- For the present analysis, a big part of computation time was spent on the needed *on-the-fly* import of the MS data from the original data files. CDF files might not be the most efficient data storage container for such on-demand data retrieval. Storing the original MS data in an SQL database (either SQLite or MariaDB/MySQL) can further improve performance. See the *MsbackendSql* for details. The code that could be used to store the full MS data of the current experiment into a SQLite database is shown below:

```
library(RSQLite)

#' Create a SQLite database
con <- dbConnect(SQLite(), "twins.sqlite")

library(MsBackendSql)
twins_db <- twins

#' Save the full MS data into this database
twins_db@spectra <- setBackend(twins_db@spectra, MsBackendSql(), dbcon = con)

dbDisconnect(con)
```

Session information

The R code was run on:

```
date()
#> [1] "Sat Nov 15 15:02:48 2025"
```

Information on the computer system:

```
library(benchmarkme)
get_cpu()
#> $vendor_id
#> [1] "GenuineIntel"
#>
#> $model_name
#> [1] "13th Gen Intel(R) Core(TM) i7-1370P"
#>
#> $no_of_cores
#> [1] 20
get_ram()
#> 65 GB
```

Information on the R session:

```r
sessionInfo()
#> R version 4.5.2 (2025-10-31)
#> Platform: x86_64-pc-linux-gnu
#> Running under: Arch Linux
#>
#> Matrix products: default
#> BLAS:   /home/jo/R/R-4.5.2/lib64/R/lib/libRblas.so
#> LAPACK: /usr/lib/liblapack.so.3.12.0  LAPACK version 3.12.0
#>
#> locale:
#>  [1] LC_CTYPE=en_US.UTF-8       LC_NUMERIC=C
#>  [3] LC_TIME=en_US.UTF-8        LC_COLLATE=en_US.UTF-8
#>  [5] LC_MONETARY=en_US.UTF-8    LC_MESSAGES=en_US.UTF-8
#>  [7] LC_PAPER=en_US.UTF-8       LC_NAME=C
#>  [9] LC_ADDRESS=C               LC_TELEPHONE=C
#> [11] LC_MEASUREMENT=en_US.UTF-8 LC_IDENTIFICATION=C
#>
#> time zone: Europe/Rome
#> tzcode source: system (glibc)
#>
#> attached base packages:
#> [1] stats4    stats     graphics  grDevices utils     datasets  methods
#> [8] base
#>
#> other attached packages:
#>  [1] benchmarkme_1.0.8           pander_0.6.6
#>  [3] peakRAM_1.0.2               xcms_4.8.0
#>  [5] MsIO_0.0.12                 MsExperiment_1.12.0
#>  [7] ProtGenerics_1.42.0         MsBackendMetaboLights_1.4.2
#>  [9] Spectra_1.20.0              BiocParallel_1.44.0
#> [11] S4Vectors_0.48.0            BiocGenerics_0.56.0
#> [13] generics_0.1.4              BiocStyle_2.38.0
#> [15] knitr_1.50
#>
#> loaded via a namespace (and not attached):
#>   [1] DBI_1.2.3                    httr2_1.2.1
#>   [3] rlang_1.1.6                  magrittr_2.0.4
#>   [5] clue_0.3-66                  MassSpecWavelet_1.76.0
#>   [7] matrixStats_1.5.0            compiler_4.5.2
#>   [9] RSQLite_2.4.4                vctrs_0.6.5
#>  [11] reshape2_1.4.5               stringr_1.6.0
#>  [13] pkgconfig_2.0.3              MetaboCoreUtils_1.18.0
#>  [15] crayon_1.5.3                 fastmap_1.2.0
#>  [17] dbplyr_2.5.1                 XVector_0.50.0
#>  [19] rmarkdown_2.30               preprocessCore_1.72.0
#>  [21] purrr_1.2.0                  bit_4.6.0
#>  [23] xfun_0.54                    MultiAssayExperiment_1.36.0
#>  [25] cachem_1.1.0                 jsonlite_2.0.0
#>  [27] progress_1.2.3               blob_1.2.4
#>  [29] rhdf5filters_1.22.0          DelayedArray_0.36.0
#>  [31] Rhdf5lib_1.32.0              parallel_4.5.2
#>  [33] prettyunits_1.2.0            cluster_2.1.8.1
```

```
#>  [35] R6_2.6.1                    stringi_1.8.7
#>  [37] RColorBrewer_1.1-3          limma_3.66.0
#>  [39] GenomicRanges_1.62.0        iterators_1.0.14
#>  [41] Rcpp_1.1.0                  Seqinfo_1.0.0
#>  [43] SummarizedExperiment_1.40.0 IRanges_2.44.0
#>  [45] BiocBaseUtils_1.12.0        Matrix_1.7-4
#>  [47] igraph_2.2.1                tidyselect_1.2.1
#>  [49] dichromat_2.0-0.1           abind_1.4-8
#>  [51] yaml_2.3.10                 doParallel_1.0.17
#>  [53] affy_1.88.0                 codetools_0.2-20
#>  [55] curl_7.0.0                  lattice_0.22-7
#>  [57] tibble_3.3.0                plyr_1.8.9
#>  [59] withr_3.0.2                 Biobase_2.70.0
#>  [61] S7_0.2.0                    benchmarkmeData_1.0.4
#>  [63] evaluate_1.0.5              BiocFileCache_3.0.0
#>  [65] alabaster.schemas_1.10.0    affyio_1.80.0
#>  [67] pillar_1.11.1               BiocManager_1.30.27
#>  [69] filelock_1.0.3              MatrixGenerics_1.22.0
#>  [71] foreach_1.5.2               MALDIquant_1.22.3
#>  [73] MSnbase_2.36.0              ncdf4_1.24
#>  [75] hms_1.1.4                   ggplot2_4.0.1
#>  [77] scales_1.4.0                alabaster.base_1.10.0
#>  [79] glue_1.8.0                  MsFeatures_1.18.0
#>  [81] lazyeval_0.2.2              tools_4.5.2
#>  [83] mzID_1.48.0                 data.table_1.17.8
#>  [85] vsn_3.78.0                  QFeatures_1.20.0
#>  [87] mzR_2.44.0                  XML_3.99-0.20
#>  [89] fs_1.6.6                    rhdf5_2.54.0
#>  [91] grid_4.5.2                  impute_1.84.0
#>  [93] tidyr_1.3.1                 MsCoreUtils_1.21.0
#>  [95] PSMatch_1.14.0              cli_3.6.5
#>  [97] rappdirs_0.3.3              S4Arrays_1.10.0
#>  [99] dplyr_1.1.4                 AnnotationFilter_1.34.0
#> [101] pcaMethods_2.2.0            gtable_0.3.6
#> [103] digest_0.6.38               SparseArray_1.10.1
#> [105] farver_2.1.2                memoise_2.0.1
#> [107] htmltools_0.5.8.1           lifecycle_1.0.4
#> [109] httr_1.4.7                  statmod_1.5.1
#> [111] bit64_4.6.0-1               MASS_7.3-65
```
