# Supplementary material for: xcms in Peak Form: Now Anchoring a Complete Metabolomics Data Preprocessing and Analysis Software Ecosystem
Source: Anal Chem. 2025 Dec 8;97(50):27639–45. doi: 10.1021/acs.analchem.5c04338 (PMC12750407; doi:10.1021/acs.analchem.5c04338)
Supplement: Supplementary file 5 [file ac5c04338_si_005.pdf]

Packages surrounding the *xcms* ecosystem and their respective information

Supporting Information for: xcms in peak form: *Now anchoring a complete metabolomics data preprocessing and analysis software ecosystem*

Philippine Louail[1,2], Carl Brunius[3], Mar Garcia-Aloy[4], William Kumler[5], Norman Storz[6], Jan Stanstrup[7], Hendrik Treutler[6], Pablo Vangeenderhuysen[8], Michael Witting[9,10], Steffen Neumann*[6,11], Johannes Rainer[1].

[1]Institute for Biomedicine, Eurac Research, 39100, Bolzano, Italy.

[2]Chair for Bioinformatics, Friedrich-Schiller-University Jena, 07743, Jena, Germany.

[3]Department of Life Sciences, Food and Nutrition Science, Chalmers University of Technology, SE-412 96 Göteborg, Sweden.

[4]Metabolomics Unit, Research and Innovation Centre, Fondazione Edmund Mach, 38098, San Michele all'Adige (TN), Italy.

[5]School of Oceanography, University of Washington, Seattle, WA, 98195, USA.

[6]Leibniz Institute of Plant Biochemistry, MetaCom, Weinberg 3, 06120 Halle, Germany.

[7]Department of Nutrition, Exercise and Sports, University of Copenhagen, Rolighedsvej 26, 1958 Frederiksberg C, Denmark.

[8]Laboratory of Integrative Metabolomics (LIMET), Ghent University, 9820 Merelbeke, Belgium.

[9]Metabolomics and Proteomics Core, Helmholtz Zentrum München, Ingolstädter Landstraße 1, 85764 Neuherberg, Germany.

[10]Chair of Analytical Food Chemistry, TUM School of Life Sciences, Technical University of Munich, Maximus-von-Imhof-Forum 2, 85354 Freising-Weihenstephan, Germany.

[11]German Centre for Integrative Biodiversity Research (iDiv) Halle-Jena-Leipzig, Puschstraße 4, 04103 Leipzig, Germany.

Abstract

This document provides a comprehensive description of the software packages comprising the xcms ecosystem, detailing the landscape visualized in Figure 2 of the main manuscript. The listing (Table S1) encompasses essential infrastructure for Liquid Chromatography-Mass Spectrometry (LC-MS) data handling, annotation, and downstream analysis. Furthermore, it categorizes available tools for cross-platform interoperability and lists educational resources and tutorials designed to assist users.

Category	Package Name	Description	Development Status	Repository Link
**Data Import**	mzR	Enables direct import of mass spectrometry data from mzML and CDF files into an MsExperiment object.	Active, Bioconductor	mzR
	MsBackendMetabolights	Provides an MsBackend to retrieve MS data from the MetaboLights repository.	Active, Bioconductor	MsBackendMetabolights
	MsBackendSql	Supports storage and access of large-scale mass spectrometry data using SQL databases via the Spectra R package.	Active, Bioconductor	MsBackendSql
**Data Import & Infrastructure**	Spectra	Provides low-level infrastructure for handling mass spectrometry spectra.	Active, Bioconductor	Spectra
**Infrastructure**	MsExperiment	Facilitates management and processing of mass spectrometry experiments.	Active, Bioconductor	MsExperiment
	Chromatograms	Supports low-level handling of chromatographic data.	Active	Chromatograms
**Compounding**	MsFeatures	Provides functionality for analyzing mass spectrometry features.	Active	MsFeatures
**Compounding & Annotation**	MetaboCoreUtils	Core utilities for metabolomics data processing.	Active, Bioconductor	MetaboCoreUtils
**Annotation**	MetaboAnnotation	High-level functionality for supporting and simplifying metabolomics data annotation.	Active, Bioconductor	MetaboAnnotation
	CompoundDb	Enables creation and management of chemical compound databases.	Active, Bioconductor	CompoundDb
	AnnotationHub	Provides access to the Bioconductor AnnotationHub web resource for annotation retrieval.	Active, Bioconductor	AnnotationHub
	MsBackendMsp	Mass spectrometry data backend for MSP files.	Active, Bioconductor	MsBackendMsp
	MsBackendMgf	Mass spectrometry data backend for Mascot Generic Format (MGF) files.	Active, Bioconductor	MsBackendMgf

	MsBackendMassbank	Backend for parsing and integrating MassBank records into the Spectra package.	Active, Bioconductor	MsBackendMassbank
**Downstream Data Analysis**	Bioconductor & R Ecosystem	Supports comprehensive quantitative and qualitative analysis of mass spectrometry data.	N/A	N/A
**Interoperability**	RuSirius	Provides an interface between the RforMassSpectrometry packages and the Sirius software suite for metabolite identification.	In Development	RuSirius
	MsIO	Supports serialization and import of mass spectrometry data objects, with potential for language-agnostic formats.	Active	MsIO
	SpectriPy	Bridges R's Spectra package with Python for cross-platform compatibility.	Active	SpectriPy
**Education & Tutorials**	Metabonaut	Provides workflows for end-to-end LC-MS/MS data analysis.	Active	Metabonaut
	xcmsTutorials	Tutorials and workshops covering LC-MS(/MS) data pre-processing and analysis with the xcms Bioconductor package.	Active	xcmsTutorials
	SpectraTutorials	Demonstrates use cases and best practices for mass spectrometry data handling and analysis with the Spectra Bioconductor package.	Active	SpectraTutorials
Table S1. Packages surrounding the xcms ecosystem and their respective information				
